# Supplementary material for: Semisynthetic Derivatives of Selected Amaryllidaceae Alkaloids as a New Class of Antimycobacterial Agents
Source: Molecules. 2021 Oct 4;26(19):6023. doi: 10.3390/molecules26196023 (PMC8512562; doi:10.3390/molecules26196023)

Supplementary Material for:

Semisynthetic derivatives of selected Amaryllidaceae alkaloids as a new class of antimycobacterial agents

Negar Maafi[1], Abdullah Al Mamun[1], Ondřej Janďourek[2], Jana Maříková[1,3], Kateřina Breiterová[1], Adéla Diepoltová[2], Klára Konečná[2] Anna Hošťálková[1], Daniela Hulcová[1,4], Jiří Kuneš[3], Eliška Kohelová[1] Darja Koutová[5], Marcela Šafratová[1,4], Lucie Nováková[6], and Lucie Cahlíková[1,*]

1 ADINACO Research Group, Department of Pharmaceutical Botany, Faculty of Pharmacy, Charles University, Heyrovskeho 1203, 500 05 Hradec Kralove, Czech Republic; negarm@faf.cuni.cz (N.M.); breiterk@faf.cuni.cz (K.B.); almamuna@faf.cuni.cz (A.A.M.); HOSTA4AA@faf.cuni.cz (A.H.); kohelove@faf.cuni.cz (E.K.)

2 Department of Biological and Medical Sciences, Faculty of Pharmacy, Charles University, Heyrovskeho 1203, 500 05 Hradec Kralove, Czech Republic; JANDO6AA@faf.cuni.cz (O.J.); konecna@faf.cuni.cz (K.K.); diepolta@faf.cuni.cz (A.D.)

3 Department of Bioorganic and Organic Chemistry, Faculty of Pharmacy, Charles University, Heyrovskeho 1203, 500 05 Hradec Kralove, Czech Republic; marikoj2@faf.cuni.cz (J.M.); kunes@faf.cuni.cz (J.K.)

4 Department of Pharmacognosy, Faculty of Pharmacy, Charles University, Heyrovskeho 1203, 500 05 Hradec Kralove, Czech Republic; hulcovd@faf.cuni.cz (D.H.), safratom@faf.cuni.cz (M.S.)

5 Department of Medical Biochemistry, Faculty of Medicine, Charles University, Simkova 870, 500 03 Hradec Kralove, Czech Republic; koutova.darja@lfhk.cuni.cz (D.J.)

6 Department of Analytical Chemistry, Faculty of Pharmacy, Charles University, Heyrovskeho 1203, 500 05 Hradec Kralove, Czech Republic; novakoval@faf.cuni.cz (L.N.)

*Corresponding author: Prof. Lucie Cahlikova; Department of Pharmaceutical Botany, Faculty of Pharmacy, Charles University, Heyrovskeho 1203, 500 05 Hradec Kralove, Czech Republic
Tel.: + 420 495 067 311; fax: + 420 495 067 162; E-mail address: cahlikova@faf.cuni.cz; ORCID ID: 0000-0002-1555-8870

Table of Contents

Figure S1. ESI-HRMS spectrum of 6-*O*-benzoylgalanthamine (**1a**)........ 5
Figure S2. ^{1}H NMR spectrum of 6-*O*-benzoylgalanthamine (**1a**) in $CDCl_3$........ 5
Figure S3. ^{13}C NMR spectrum of 6-*O*-benzoylgalanthamine (**1a**) in $CDCl_3$........ 5
Figure S4. ESI-HRMS spectrum of 6-*O*-(2-methylbenzoyl)galanthamine (**1b**)........ 6
Figure S5. ^{1}H NMR spectrum of 6-*O*-(2-methylbenzoyl)galanthamine (**1b**) in $CDCl_3$........ 6
Figure S6. ^{13}C NMR spectrum of 6-*O*-(2-methylbenzoyl)galanthamine (**1b**) in $CDCl_3$........ 6
Figure S7. ESI-HRMS spectrum of 6-*O*-(3-methylbenzoyl)galanthamine (**1c**)........ 7
Figure S8. ^{1}H NMR spectrum of 6-*O*-(3-methylbenzoyl)galanthamine (**1c**) in $CDCl_3$........ 7
Figure S9. ^{13}C NMR spectrum of 6-*O*-(3-methylbenzoyl)galanthamine (**1c**) in $CDCl_3$........ 7
Figure S10. ESI-HRMS spectrum of 6-*O*-(4-methylbenzoyl)galanthamine (**1d**)........ 8
Figure S11. ^{1}H NMR spectrum of 6-*O*-(4-methylbenzoyl)galanthamine (**1d**) in $CDCl_3$........ 8
Figure S12. ^{13}C NMR spectrum of 6-*O*-(4-methylbenzoyl)galanthamine (**1d**) in $CDCl_3$........ 8
Figure S13. ESI-HRMS spectrum of 6-*O*-(2,3-dimethylbenzoyl)galanthamine (**1e**)........ 9
Figure S14. ^{1}H NMR spectrum of 6-*O*-(2,3-dimethylbenzoyl)galanthamine (**1e**) in $CDCl_3$........ 9
Figure S15. ^{13}C NMR spectrum of 6-*O*-(2,3-dimethylbenzoyl)galanthamine (**1e**) in $CDCl_3$........ 9
Figure S16. ESI-HRMS spectrum of 6-*O*-(3,5-dimethylbenzoyl)galanthamine (**1f**)........ 10
Figure S17. ^{1}H NMR spectrum of 6-*O*-(3,5-dimethylbenzoyl)galanthamine (**1f**) in $CDCl_3$........ 10
Figure S18. ^{13}C NMR spectrum of 6-*O*-(3,5-dimethylbenzoyl)galanthamine (**1f**) in $CDCl_3$........ 10
Figure S19. ESI-HRMS spectrum of 6-*O*-(2,4,6-trimethylbenzoyl)galanthamine (**1g**)........ 11
Figure S20. ^{1}H NMR spectrum of 6-*O*-(2,4,6-trimethylbenzoyl)galanthamine (**1g**) in $CDCl_3$........ 11
Figure S21. ^{13}C NMR spectrum of 6-*O*-(2,4,6-trimethylbenzoyl)galanthamine (**1g**) in $CDCl_3$........ 11
Figure S22. ESI-HRMS spectrum of 6-*O*-(4-tert-butylbenzoyl)galanthamine (**1h**)........ 12
Figure S23. ^{1}H NMR spectrum of 6-*O*-(4-tert-butylbenzoyl)galanthamine (**1h**) in $CDCl_3$........ 12
Figure S24. ^{13}C NMR spectrum of 6-*O*-(4-tert-butylbenzoyl)galanthamine (**1h**) in $CDCl_3$........ 12
Figure S25. ESI-HRMS spectrum of 6-*O*-(4-butylbenzoyl)galanthamine (**1i**)........ 13
Figure S26. ^{1}H NMR spectrum of 6-*O*-(4-butylbenzoyl)galanthamine (**1i**) in $CDCl_3$........ 13
Figure S27. ^{13}C NMR spectrum of 6-*O*-(4-butylbenzoyl)galanthamine (**1i**) in $CDCl_3$........ 13
Figure S28. ESI-HRMS spectrum of 6-*O*-(2-methoxybenzoyl)galanthamine (**1j**)........ 14
Figure S29. ^{1}H NMR spectrum of 6-*O*-(2-methoxybenzoyl)galanthamine (**1j**) in $CDCl_3$........ 14
Figure S30. ^{13}C NMR spectrum of 6-*O*-(2-methoxybenzoyl)galanthamine (**1j**) in $CDCl_3$........ 14
Figure S31. ESI-HRMS spectrum of 6-*O*-(3-methoxybenzoyl)galanthamine (**1k**)........ 15
Figure S32. ^{1}H NMR spectrum of 6-*O*-(3-methoxybenzoyl)galanthamine (**1k**) in $CDCl_3$........ 15
Figure S33. ^{13}C NMR spectrum of 6-*O*-(3-methoxybenzoyl)galanthamine (**1k**) in $CDCl_3$........ 15
Figure S34. ESI-HRMS spectrum of 6-*O*-(4-methoxybenzoyl)galanthamine (**1l**)........ 16
Figure S35. ^{1}H NMR spectrum of 6-*O*-(4-methoxybenzoyl)galanthamine (**1l**) in $CDCl_3$........ 16
Figure S36. ^{13}C NMR spectrum of 6-*O*-(4-methoxybenzoyl)galanthamine (**1l**) in $CDCl_3$........ 16

Figure S37. ESI-HRMS spectrum of 6-*O*-(3,4-dimethoxybenzoyl)galanthamine (**1m**) 17
Figure S38. ^{1}H NMR spectrum of 6-*O*-(3,4-dimethoxybenzoyl)galanthamine (**1m**) in $CDCl_3$....... 17
Figure S39. ^{13}C NMR spectrum of 6-*O*-(3,4-dimethoxybenzoyl)galanthamine (**1m**) in $CDCl_3$...... 17
Figure S40. ESI-HRMS spectrum of 6-*O*-(3,5-dimethoxybenzoyl)galanthamine (**1n**) 18
Figure S41. ^{1}H NMR spectrum of 6-*O*-(3,5-dimethoxybenzoyl)galanthamine (**1n**) in $CDCl_3$........ 18
Figure S42. ^{13}C NMR spectrum of 6-*O*-(3,5-dimethoxybenzoyl)galanthamine (**1n**) in $CDCl_3$...... 18
Figure S43. ESI-HRMS spectrum of 6-*O*-(3,4,5-trimethoxybenzoyl)galanthamine (**1o**) 19
Figure S44. ^{1}H NMR spectrum of 6-*O*-(3,4,5-trimethoxybenzoyl)galanthamine (**1o**) in $CDCl_3$..... 19
Figure S45. ^{13}C NMR spectrum of 6-*O*-(3,4,5-trimethoxybenzoyl)galanthamine (**1o**) in $CDCl_3$.... 19
Figure S46. ESI-HRMS spectrum of 6-*O*-(3,5-diethoxybenzoyl)galanthamine (**1p**) 20
Figure S47. ^{1}H NMR spectrum of 6-*O*-(3,5-diethoxybenzoyl)galanthamine (**1p**) in $CDCl_3$........... 20
Figure S48. ^{13}C NMR spectrum of 6-*O*-(3,5-diethoxybenzoyl)galanthamine (**1p**) in $CDCl_3$.......... 20
Figure S49. ESI-HRMS spectrum of 6-*O*-(1-naphtoyl)galanthamine (**1q**)..................................... 21
Figure S50. ^{1}H NMR spectrum of 6-*O*-(1-naphtoyl)galanthamine (**1q**) in $CDCl_3$.......................... 21
Figure S51. ^{13}C NMR spectrum of 6-*O*-(1-naphtoyl)galanthamine (**1q**) in $CDCl_3$......................... 21
Figure S52. ESI-HRMS spectrum of 6-*O*-(2-naphtoyl)galanthamine (**1r**)...................................... 22
Figure S53. ^{1}H NMR spectrum of 6-*O*-(2-naphtoyl)galanthamine (**1r**) in $CDCl_3$.......................... 22
Figure S54. ^{13}C NMR spectrum of 6-*O*-(2-naphtoyl)galanthamine (**1r**) in $CDCl_3$......................... 22
Figure S55. ESI-HRMS spectrum of 6-*O*-(2-furoyl)galanthamine (**1s**).. 23
Figure S56. ^{1}H NMR spectrum of 6-*O*-(2-furoyl)galanthamine (**1s**) in $CDCl_3$.............................. 23
Figure S57. ^{13}C NMR spectrum of 6-*O*-(2-furoyl)galanthamine (**1s**) in $CDCl_3$............................. 23
Figure S58. ESI-HRMS spectrum of 3-*O*-methylpancracine (**3**) 24
Figure S59. ^{1}H NMR spectrum of 3-*O*-methylpancracine (**3**) in $CDCl_3$... 24
Figure S60. ^{13}C NMR spectrum of 3-*O*-methylpancracine (**3**) in $CDCl_3$.. 25
Figure S61. ESI-HRMS spectrum of 2-*O*-(3,5-dimethylbenzoyl)-3-*O*-methylpancracine (**3a**)....... 25
Figure S62. ^{1}H NMR spectrum of 2-*O*-(3,5-dimethylbenzoyl)-3-*O*-methylpancracine (**3a**) in $CDCl_3$ 25
Figure S63. ^{13}C NMR spectrum of 2-*O*-(3,5-dimethylbenzoyl)-3-*O*-methylpancracine (**3a**) in $CDCl_3$ 26
Figure S64. ESI-HRMS spectrum of 2-*O*-(2,4,6-trimethylbenzoyl)-3-*O*-methylpancracine (**3b**)... 26
Figure S65. ^{1}H NMR spectrum of 2-*O*-(2,4,6-trimethylbenzoyl)-3-*O*-methylpancracine (**3b**) in $CDCl_3$ 26
Figure S66. ^{13}C NMR spectrum of 2-*O*-(2,4,6-trimethylbenzoyl)-3-*O*-methylpancracine (**3b**) in $CDCl_3$ 27
Figure S67. ESI-HRMS spectrum of 2-*O*-(4-tert-butylbenzoyl)-3-*O*-methylpancracine (**3c**) 27
Figure S68. ^{1}H NMR spectrum of 2-*O*-(4-tert-butylbenzoyl)-3-*O*-methylpancracine (**3c**) in $CDCl_3$ 27
Figure S69. ^{13}C NMR spectrum of 2-*O*-(4-tert-butylbenzoyl)-3-*O*-methylpancracine (**3c**) in $CDCl_3$ 28

Figure S70. ESI-HRMS spectrum of 2-*O*-(4-butylbenzoyl)-3-*O*-methylpancracine (**3d**)................ 28
Figure S71. ^{1}H NMR spectrum of 2-*O*-(4-butylbenzoyl)-3-*O*-methylpancracine (**3d**) in $CDCl_3$ 29
Figure S72. ^{13}C NMR spectrum of 2-*O*-(4-butylbenzoyl)-3-*O*-methylpancracine (**3d**) in $CDCl_3$ ... 29
Figure S73. ESI-HRMS spectrum of 2-*O*-(3,5-diethoxybenzoyl)-3-*O*-methylpancracine (**3e**) 29
Figure S74. ^{1}H NMR spectrum of 2-*O*-(3,5-diethoxybenzoyl)-3-*O*-methylpancracine (**3e**) in $CDCl_3$ 30
Figure S75. ^{13}C NMR spectrum of 2-*O*-(3,5-diethoxybenzoyl)-3-*O*-methylpancracine (**3e**) in $CDCl_3$ 30
Figure S76. ESI-HRMS spectrum of 2-*O*-(1-naphtoyl)-3-*O*-methylpancracine (**3f**) 31
Figure S77. ^{1}H NMR spectrum of 2-*O*-(1-naphtoyl)-3-*O*-methylpancracine (**3f**) in $CDCl_3$............ 31
Figure S78. ^{13}C NMR spectrum of 2-*O*-(1-naphtoyl)-3-*O*-methylpancracine (**3f**) in $CDCl_3$........... 31
Figure S79. ESI-HRMS spectrum of 2-*O*-(2-naphtoyl)-3-*O*-methylpancracine (**3g**)....................... 32
Figure S80. ^{1}H NMR spectrum of 2-*O*-(2-naphtoyl)-3-*O*-methylpancracine (**3g**) in $CDCl_3$ 32
Figure S81. ^{13}C NMR spectrum of 2-*O*-(2-naphtoyl)-3-*O*-methylpancracine (**3g**) in $CDCl_3$ 32
Figure S82. ESI-HRMS spectrum of 3-*O*-(1-naphtoyl)vittatine (**4a**)... 33
Figure S83. ^{1}H NMR spectrum of 3-*O*-(1-naphtoyl)vittatine (**4a**) in $CDCl_3$ 33
Figure S84. ^{13}C NMR spectrum of 3-*O*-(1-naphtoyl)vittatine (**4a**) in $CDCl_3$ 33
Figure S85. ESI-HRMS spectrum of 3-*O*-(2-naphtoyl)vittatine (**4b**)... 34
Figure S86. ^{1}H NMR spectrum of 3-*O*-(2-naphtoyl)vittatine (**4b**) in $CDCl_3$ 34
Figure S87. ^{13}C NMR spectrum of 3-*O*-(2-naphtoyl)vittatine (**4b**) in $CDCl_3$ 34
Figure S88. ESI-HRMS spectrum of 3-*O*-(3,5-dimethylbenzoyl)maritidine (**5a**)........................... 35
Figure S89. ^{1}H NMR spectrum of 3-*O*-(3,5-dimethylbenzoyl)maritidine (**5a**) in $CDCl_3$ 35
Figure S90. ^{13}C NMR spectrum of 3-*O*-(3,5-dimethylbenzoyl)maritidine (**5a**) in $CDCl_3$ 35
Figure S91. ESI-HRMS spectrum of 3-*O*-(1-naphtoyl)maritidine (**5b**) 36
Figure S92. ^{1}H NMR spectrum of 3-*O*-(1-naphtoyl)maritidine (**5b**) in $CDCl_3$............................... 36
Figure S93. ^{13}C NMR spectrum of 3-*O*-(1-naphtoyl)maritidine (**5b**) in $CDCl_3$.............................. 36

Figure S1. ESI-HRMS spectrum of 6-*O*-benzoylgalanthamine (**1a**)

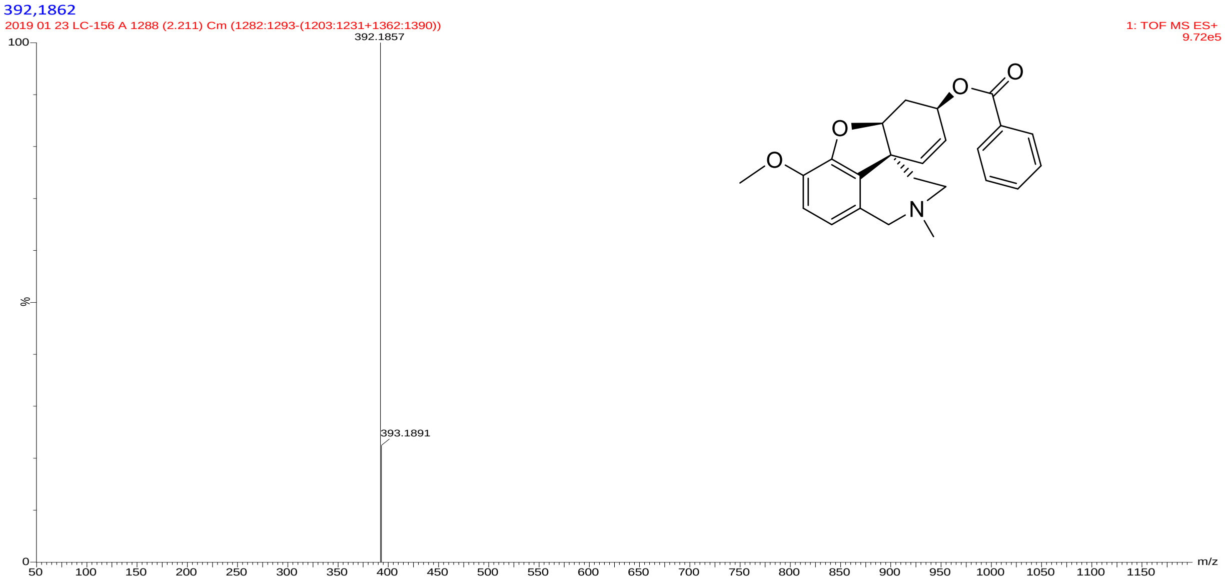

Figure S2. ^{1}H NMR spectrum of 6-*O*-benzoylgalanthamine (**1a**) in $CDCl_3$

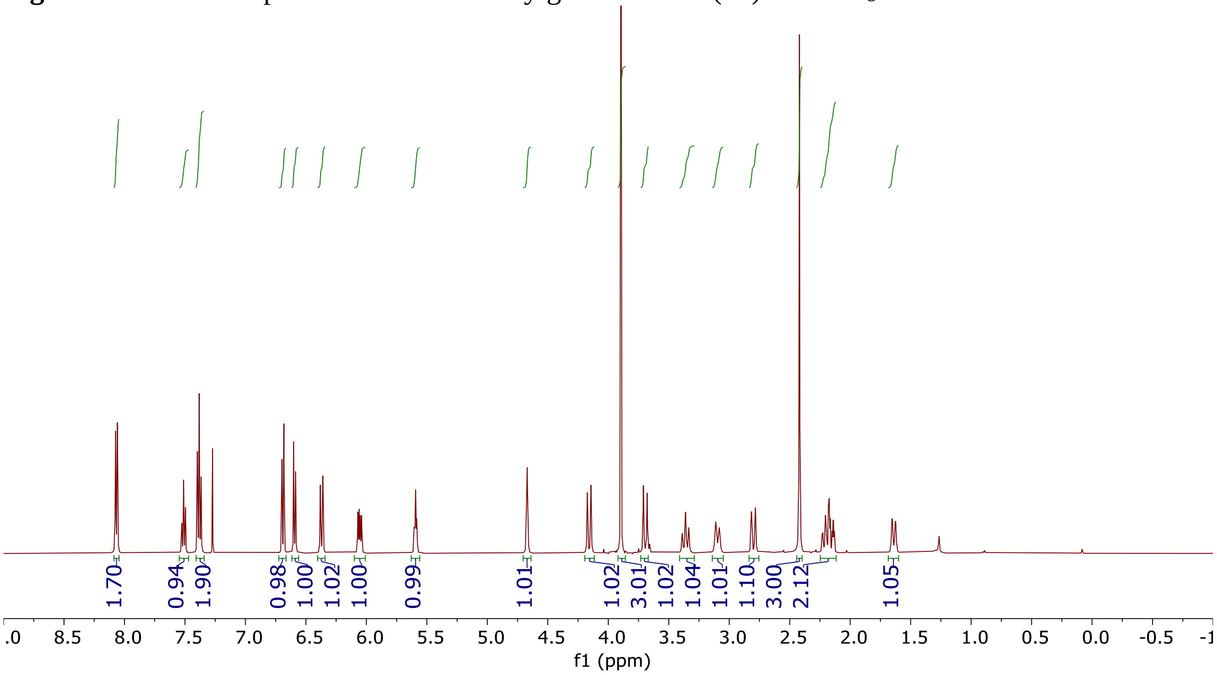

Figure S3. ^{13}C NMR spectrum of 6-*O*-benzoylgalanthamine (**1a**) in $CDCl_3$

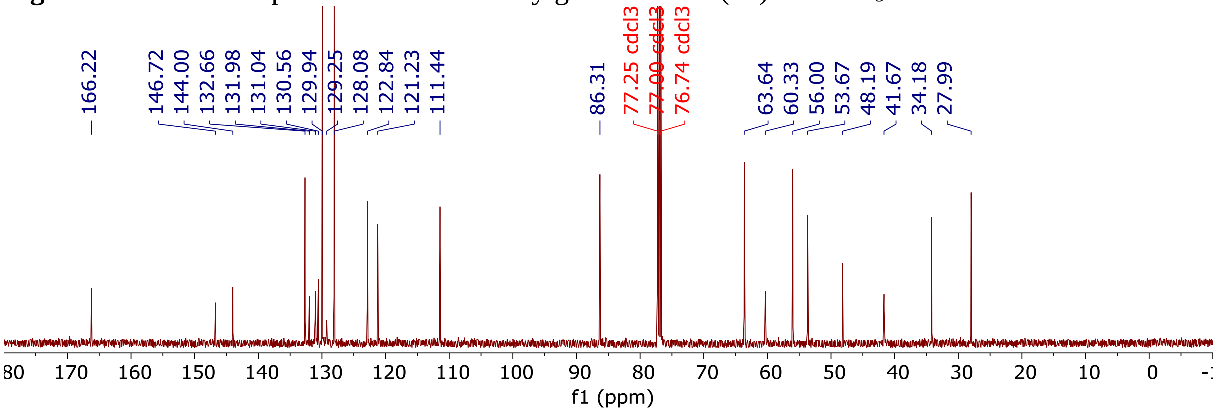

Figure S4. ESI-HRMS spectrum of 6-*O*-(2-methylbenzoyl)galanthamine (**1b**)

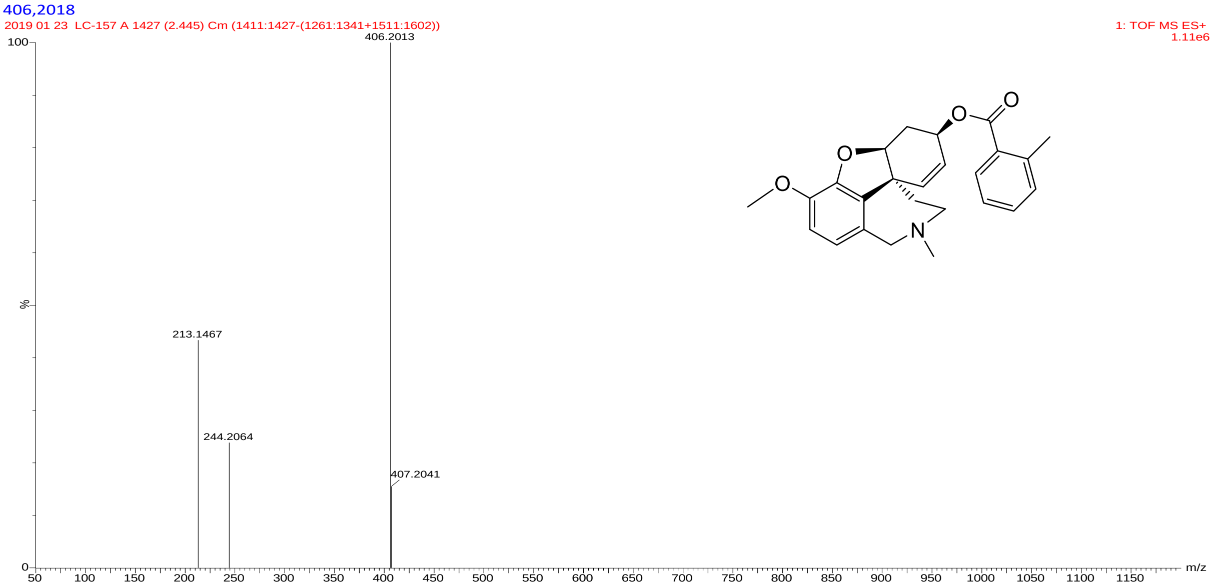

Figure S5. ^{1}H NMR spectrum of 6-*O*-(2-methylbenzoyl)galanthamine (**1b**) in $CDCl_3$

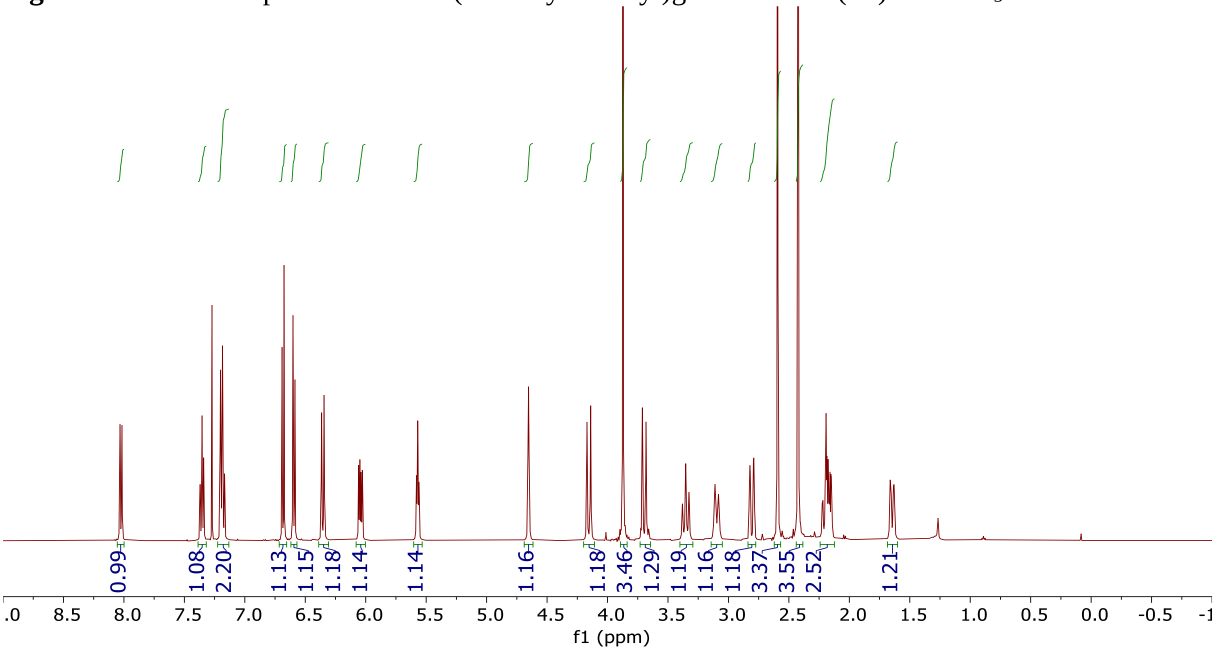

Figure S6. ^{13}C NMR spectrum of 6-*O*-(2-methylbenzoyl)galanthamine (**1b**) in $CDCl_3$

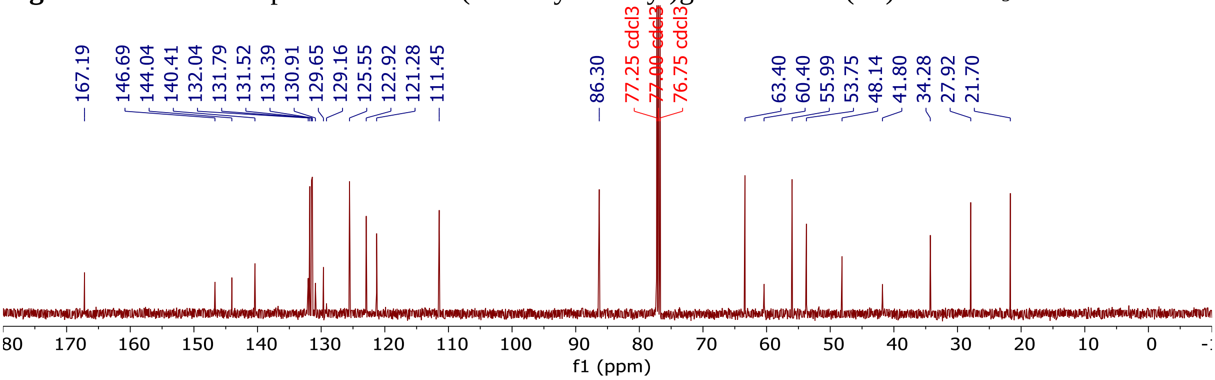

Figure S7. ESI-HRMS spectrum of 6-*O*-(3-methylbenzoyl)galanthamine (**1c**)

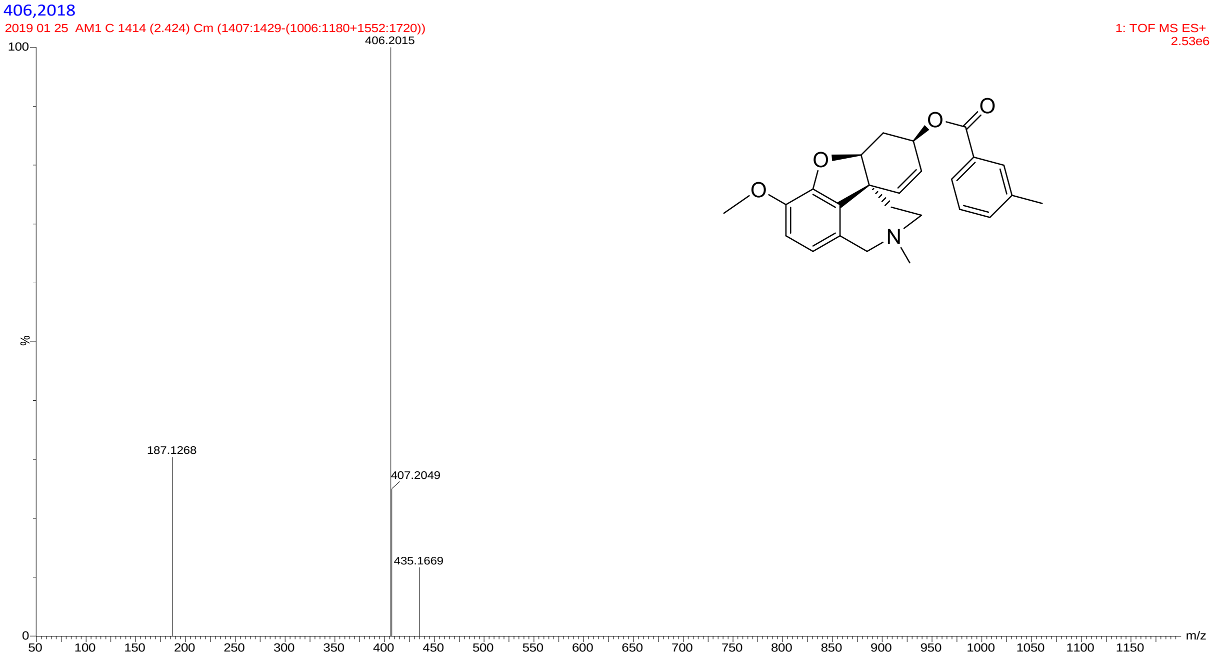

Figure S8. ^{1}H NMR spectrum of 6-*O*-(3-methylbenzoyl)galanthamine (**1c**) in $CDCl_3$

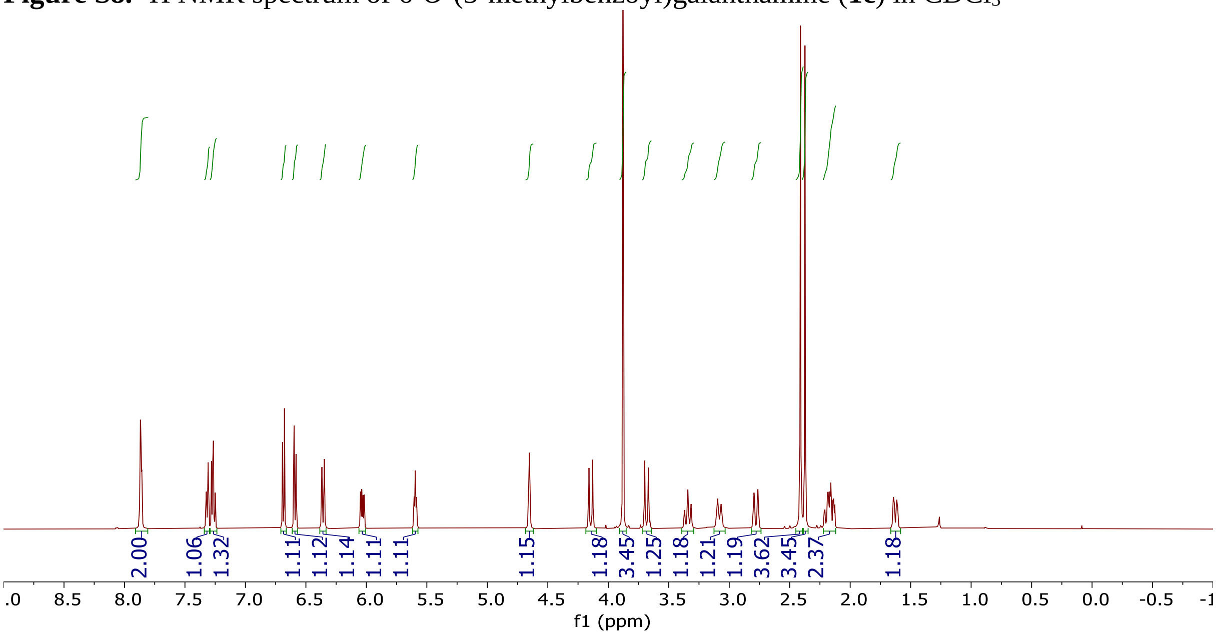

Figure S9. ^{13}C NMR spectrum of 6-*O*-(3-methylbenzoyl)galanthamine (**1c**) in $CDCl_3$

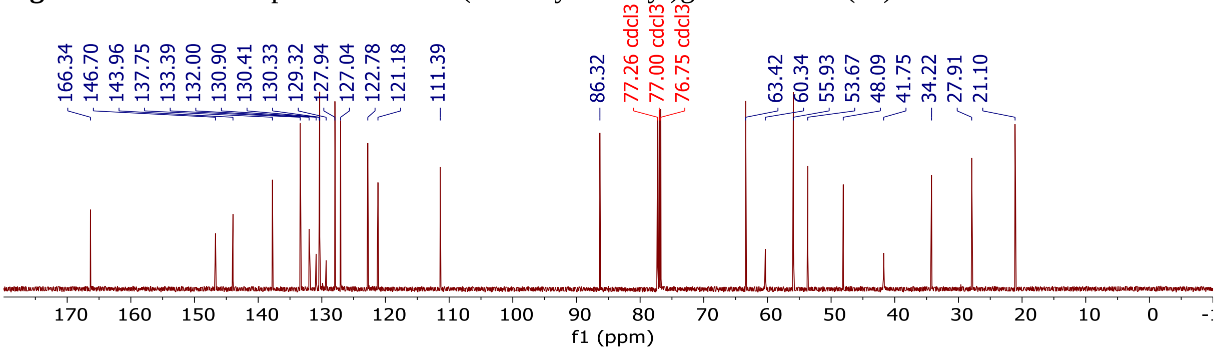

Figure S10. ESI-HRMS spectrum of 6-*O*-(4-methylbenzoyl)galanthamine (**1d**)

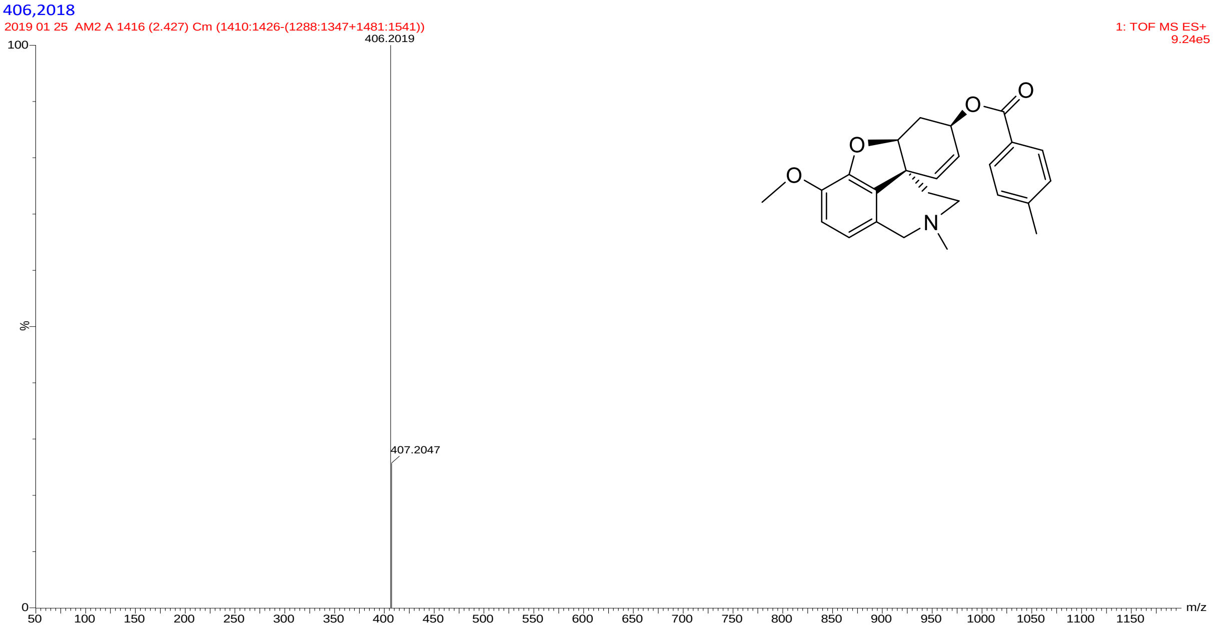

Figure S11. ^{1}H NMR spectrum of 6-*O*-(4-methylbenzoyl)galanthamine (**1d**) in $CDCl_3$

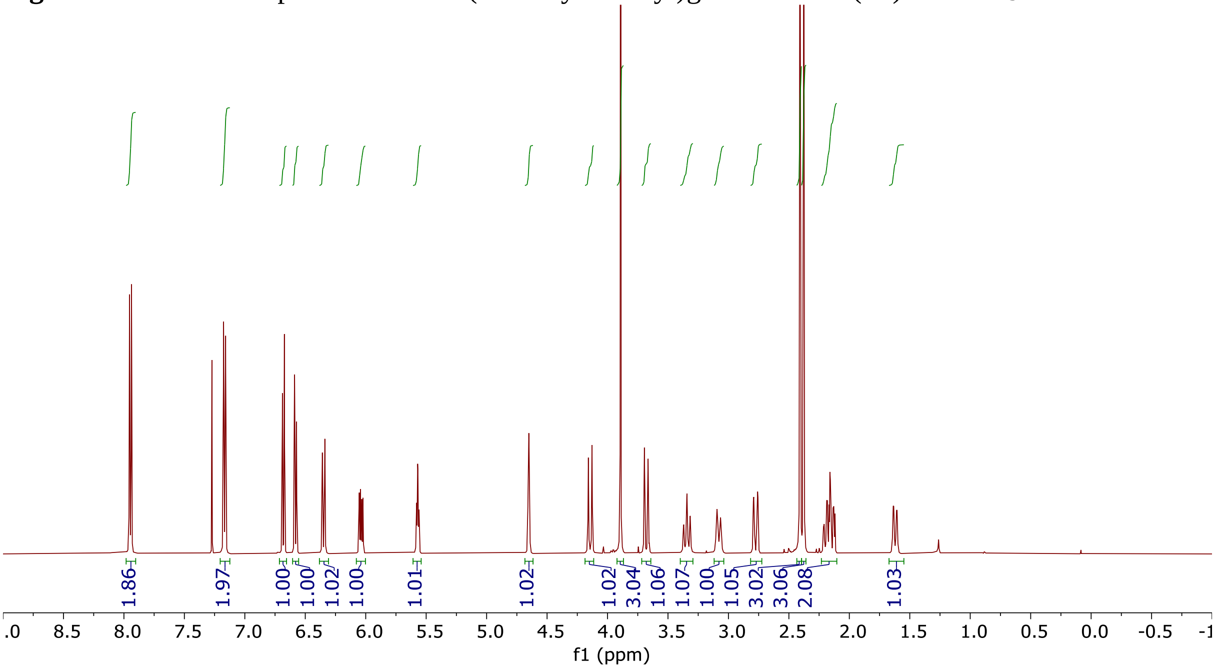

Figure S12. ^{13}C NMR spectrum of 6-*O*-(4-methylbenzoyl)galanthamine (**1d**) in $CDCl_3$

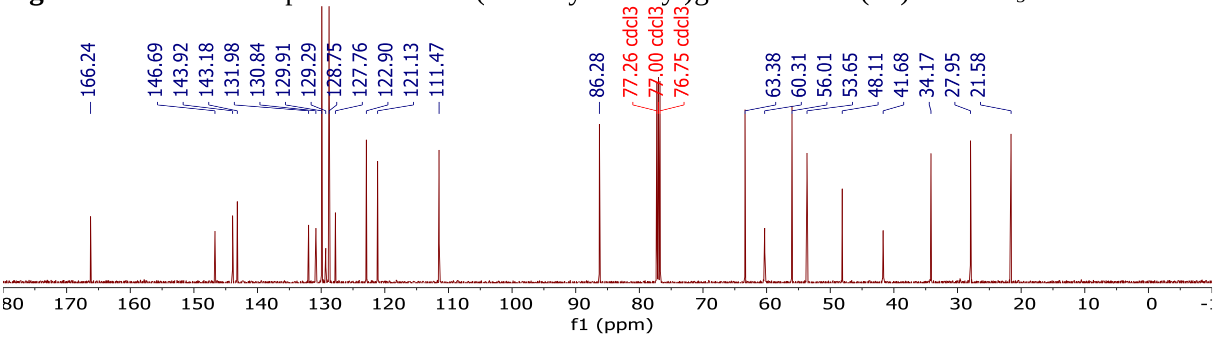

Figure S13. ESI-HRMS spectrum of 6-*O*-(2,3-dimethylbenzoyl)galanthamine (**1e**)

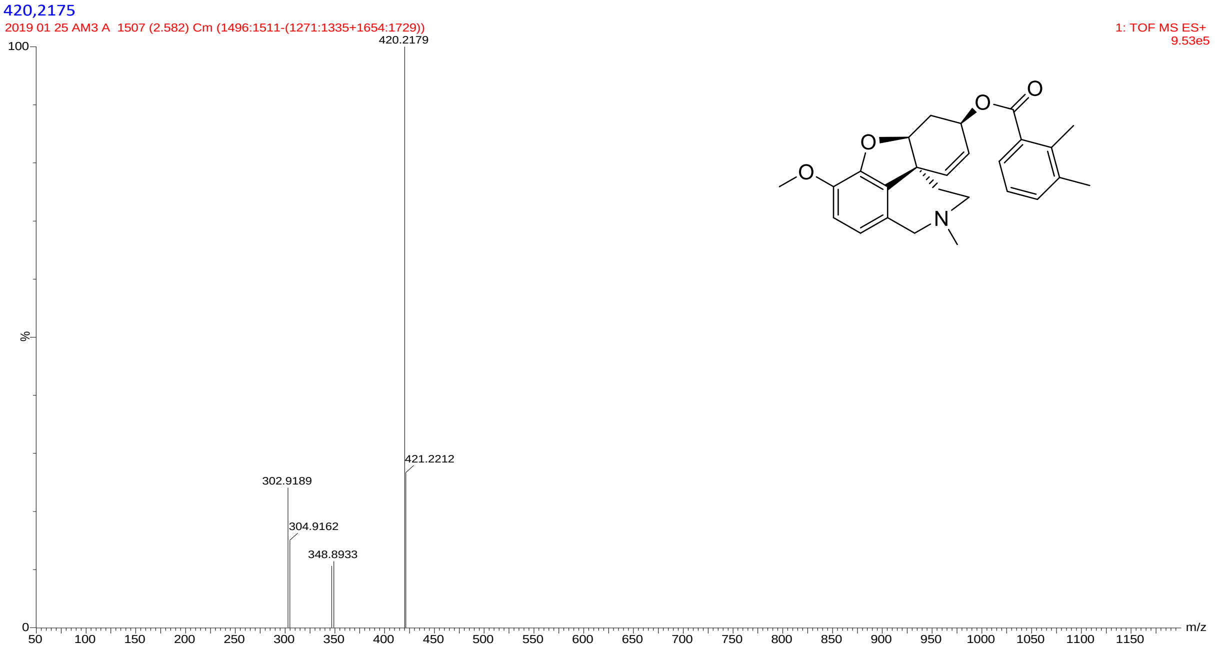

Figure S14. ^{1}H NMR spectrum of 6-*O*-(2,3-dimethylbenzoyl)galanthamine (**1e**) in $CDCl_3$

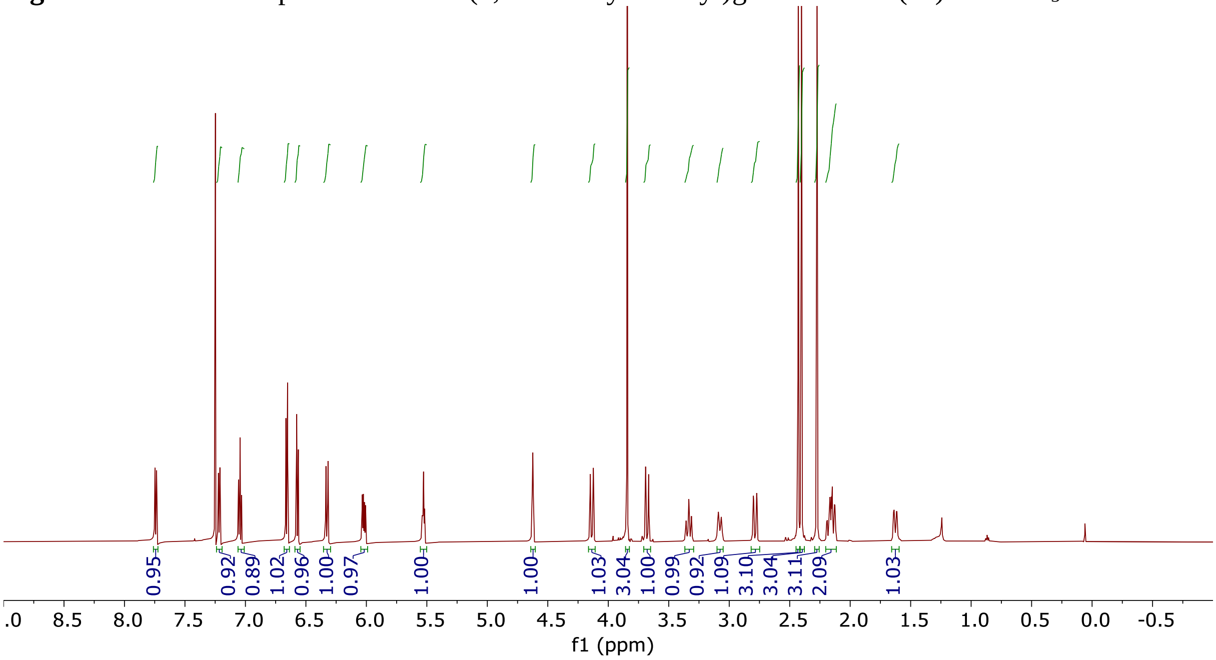

Figure S15. ^{13}C NMR spectrum of 6-*O*-(2,3-dimethylbenzoyl)galanthamine (**1e**) in $CDCl_3$

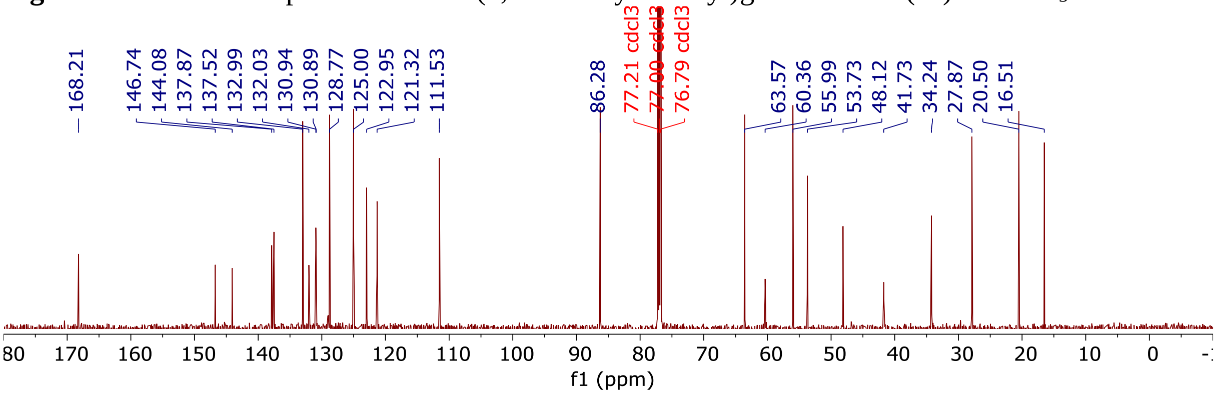

Figure S16. ESI-HRMS spectrum of 6-*O*-(3,5-dimethylbenzoyl)galanthamine (**1f**)

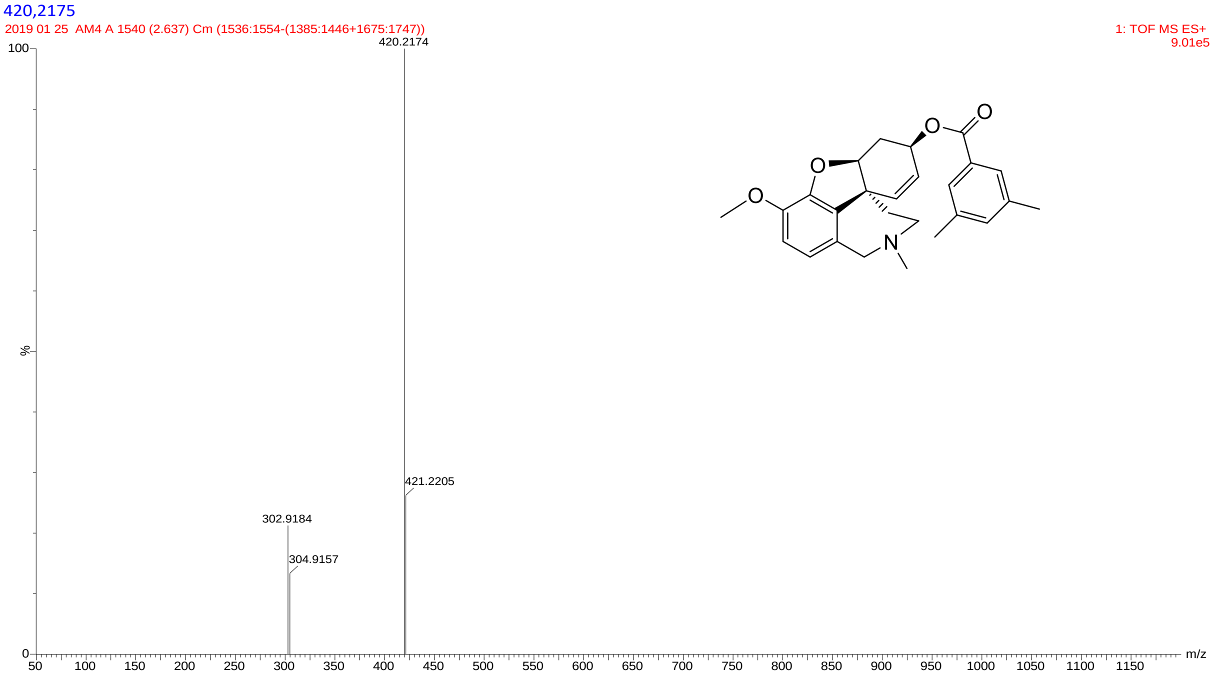

Figure S17. ^{1}H NMR spectrum of 6-*O*-(3,5-dimethylbenzoyl)galanthamine (**1f**) in $CDCl_3$

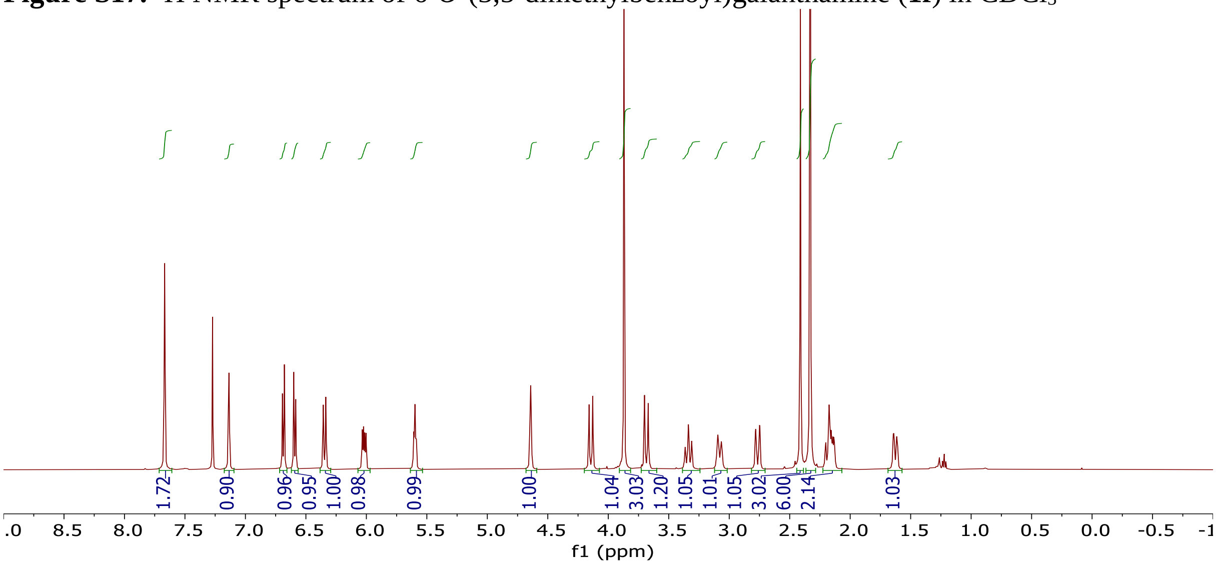

Figure S18. ^{13}C NMR spectrum of 6-*O*-(3,5-dimethylbenzoyl)galanthamine (**1f**) in $CDCl_3$

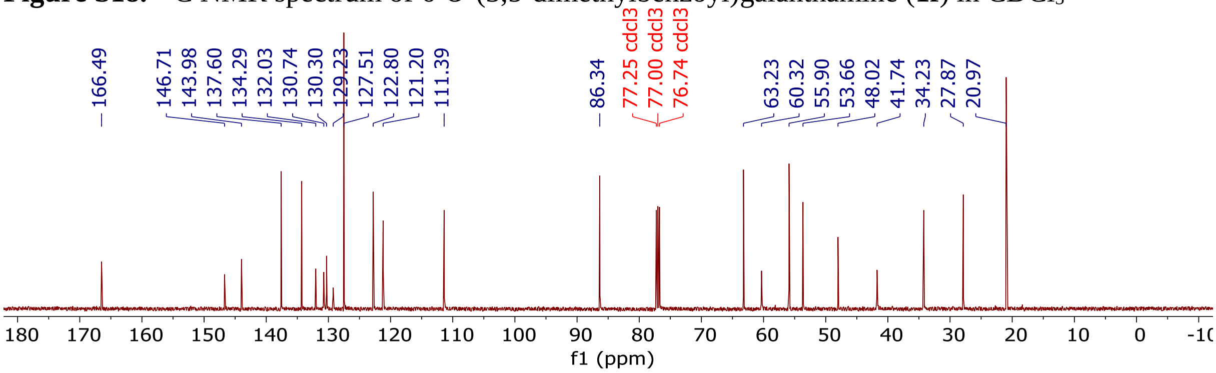

Figure S19. ESI-HRMS spectrum of 6-*O*-(2,4,6-trimethylbenzoyl)galanthamine (**1g**)

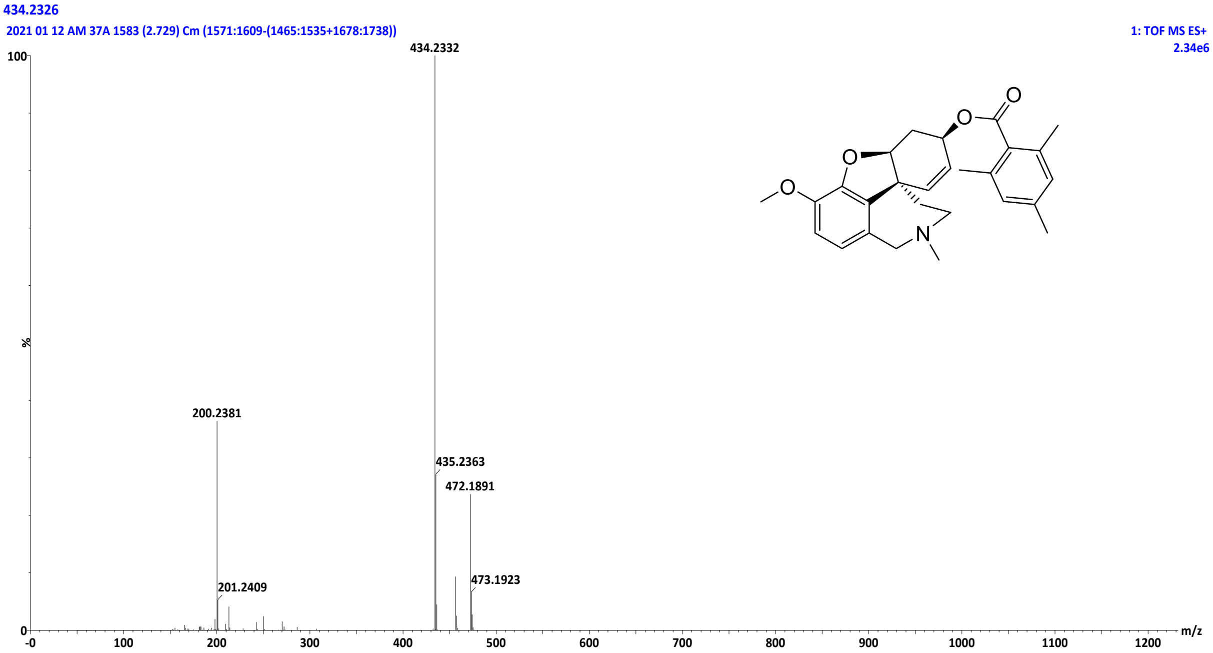

Figure S20. ^{1}H NMR spectrum of 6-*O*-(2,4,6-trimethylbenzoyl)galanthamine (**1g**) in $CDCl_3$

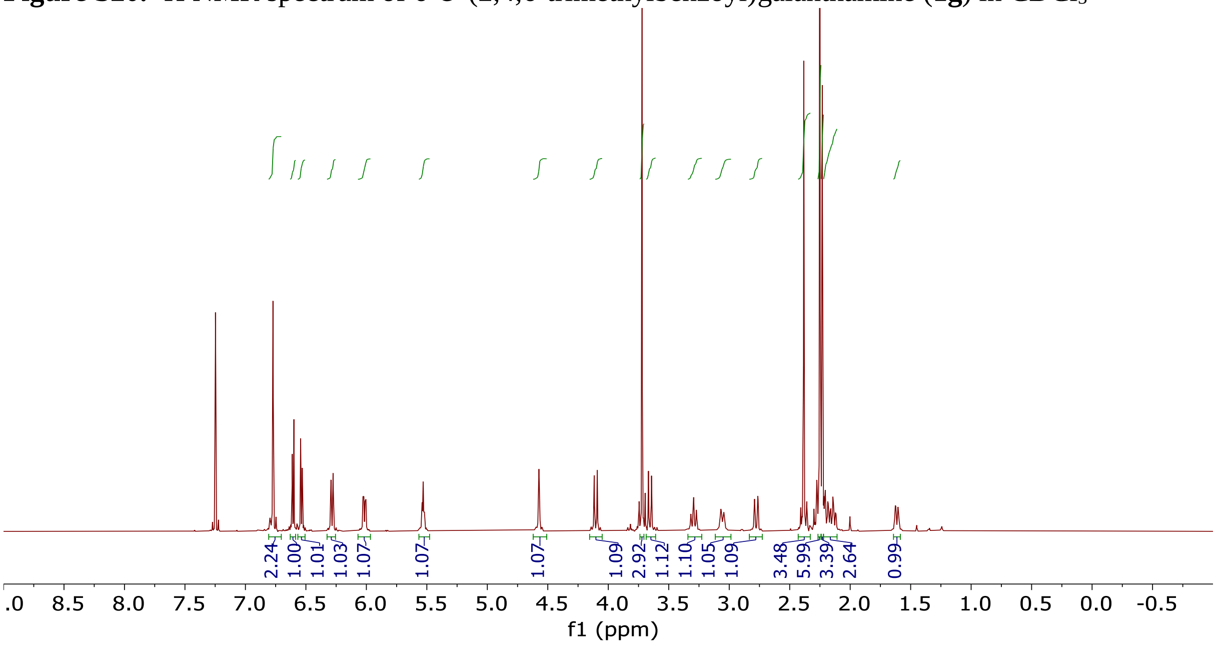

Figure S21. ^{13}C NMR spectrum of 6-*O*-(2,4,6-trimethylbenzoyl)galanthamine (**1g**) in $CDCl_3$

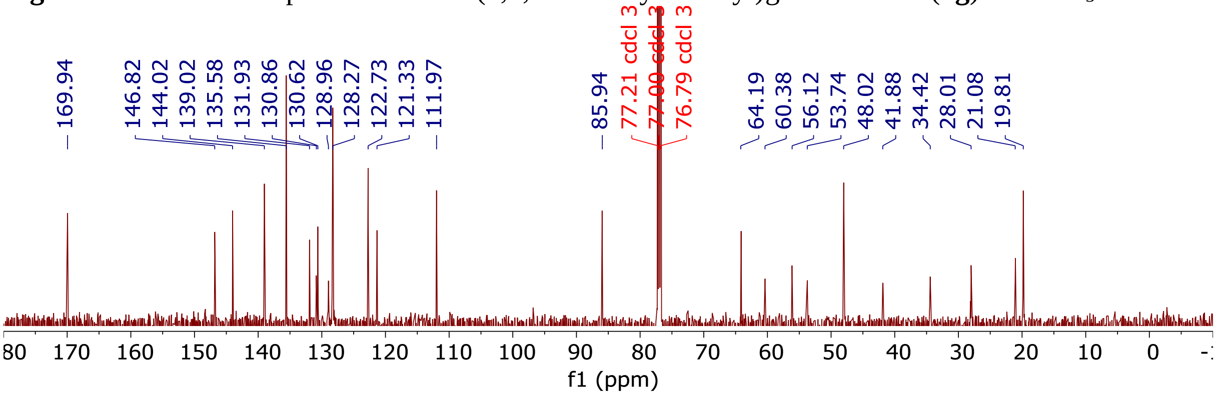

Figure S22. ESI-HRMS spectrum of 6-*O*-(4-tert-butylbenzoyl)galanthamine (**1h**)

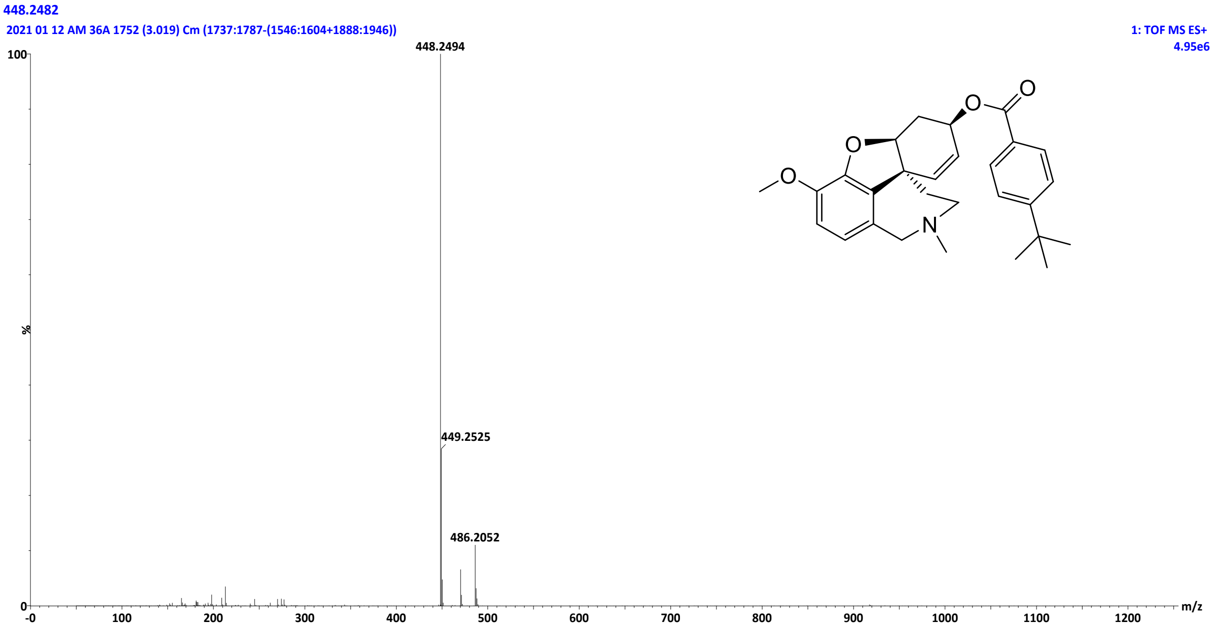

Figure S23. ^{1}H NMR spectrum of 6-*O*-(4-tert-butylbenzoyl)galanthamine (**1h**) in $CDCl_3$

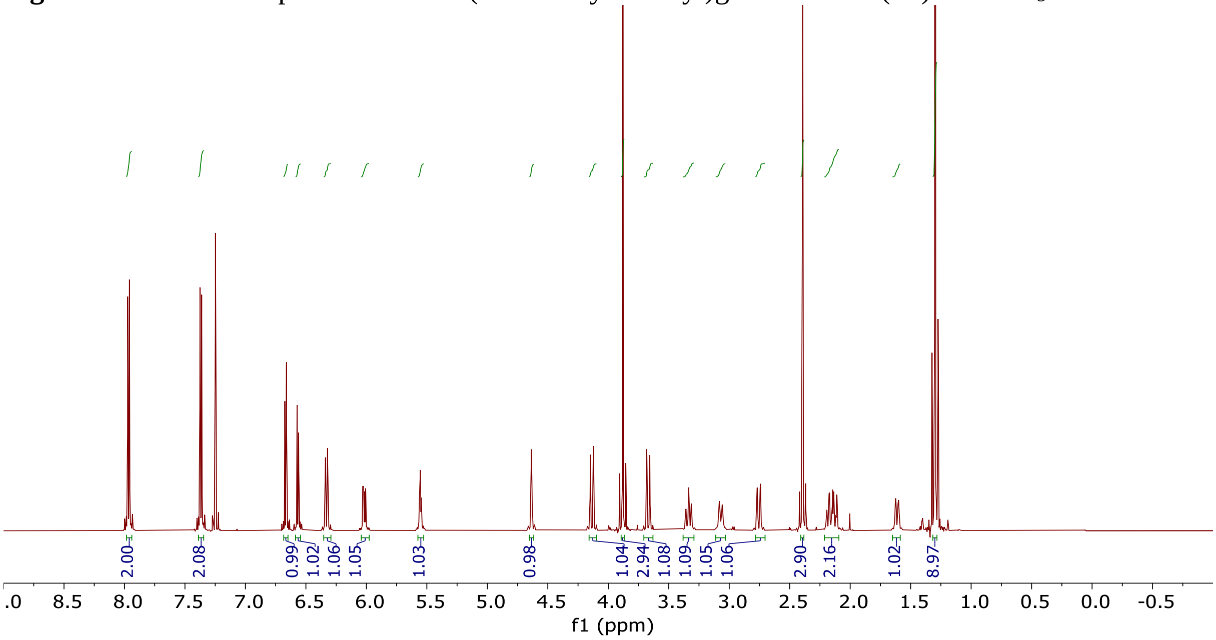

Figure S24. ^{13}C NMR spectrum of 6-*O*-(4-tert-butylbenzoyl)galanthamine (**1h**) in $CDCl_3$

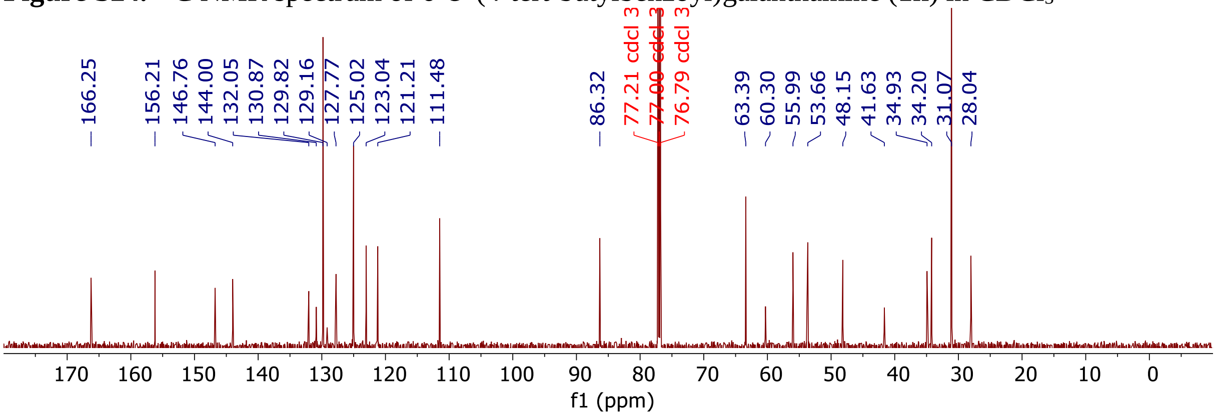

Figure S25. ESI-HRMS spectrum of 6-*O*-(4-butylbenzoyl)galanthamine (**1i**)

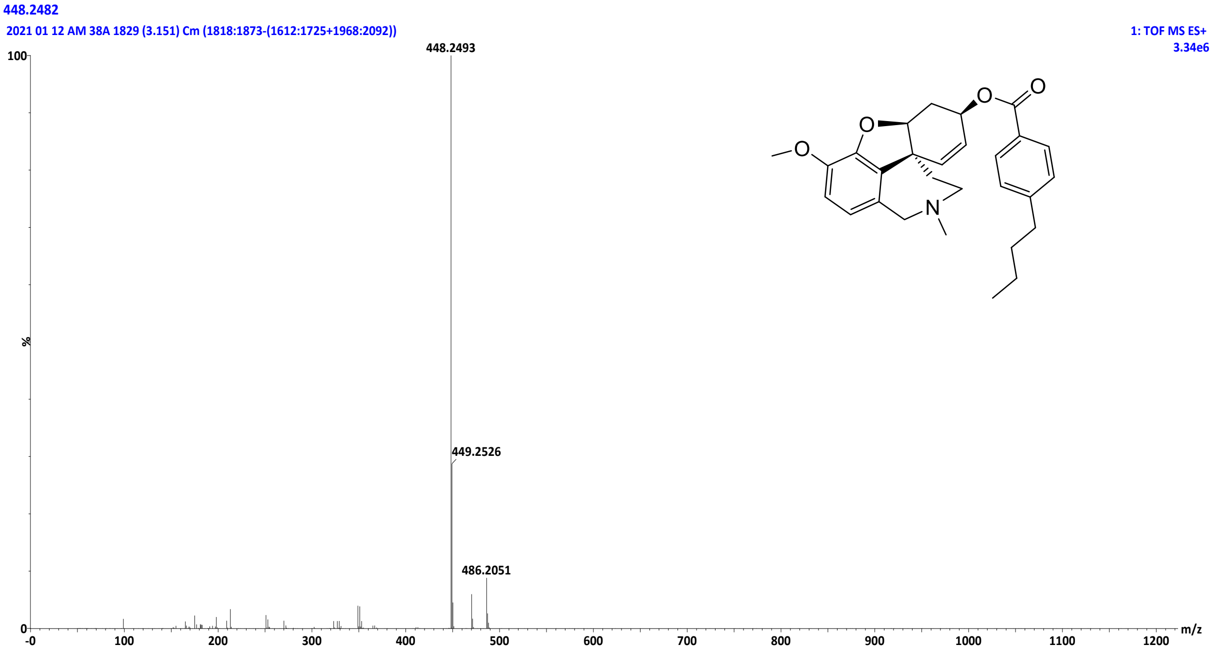

Figure S26. ^{1}H NMR spectrum of 6-*O*-(4-butylbenzoyl)galanthamine (**1i**) in $CDCl_3$

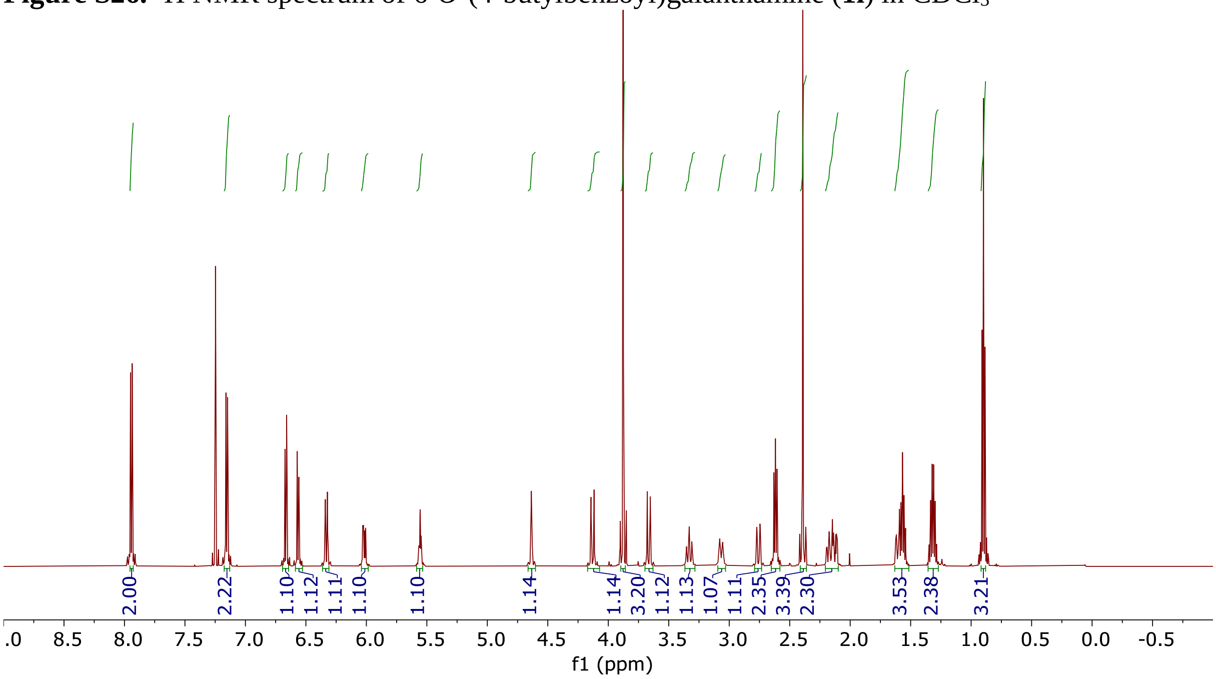

Figure S27. ^{13}C NMR spectrum of 6-*O*-(4-butylbenzoyl)galanthamine (**1i**) in $CDCl_3$

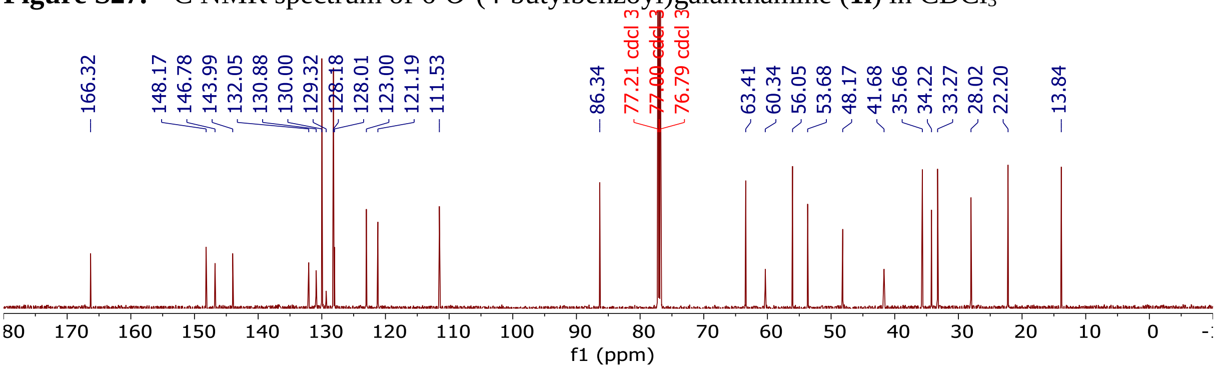

Figure S28. ESI-HRMS spectrum of 6-*O*-(2-methoxybenzoyl)galanthamine (**1j**)

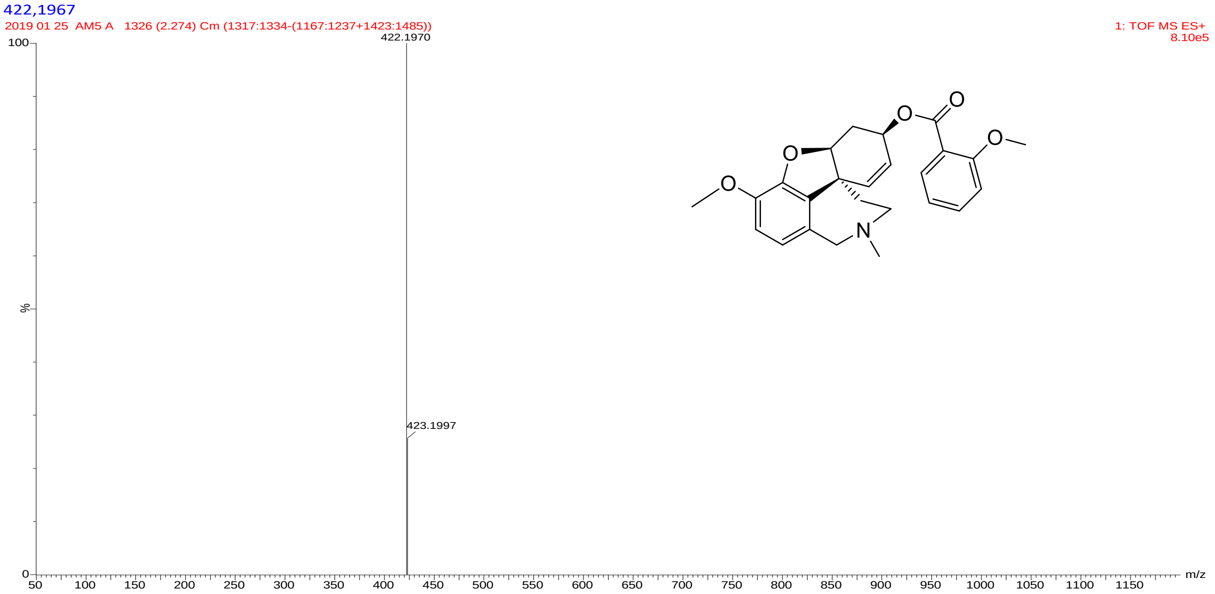

Figure S29. ^{1}H NMR spectrum of 6-*O*-(2-methoxybenzoyl)galanthamine (**1j**) in $CDCl_3$

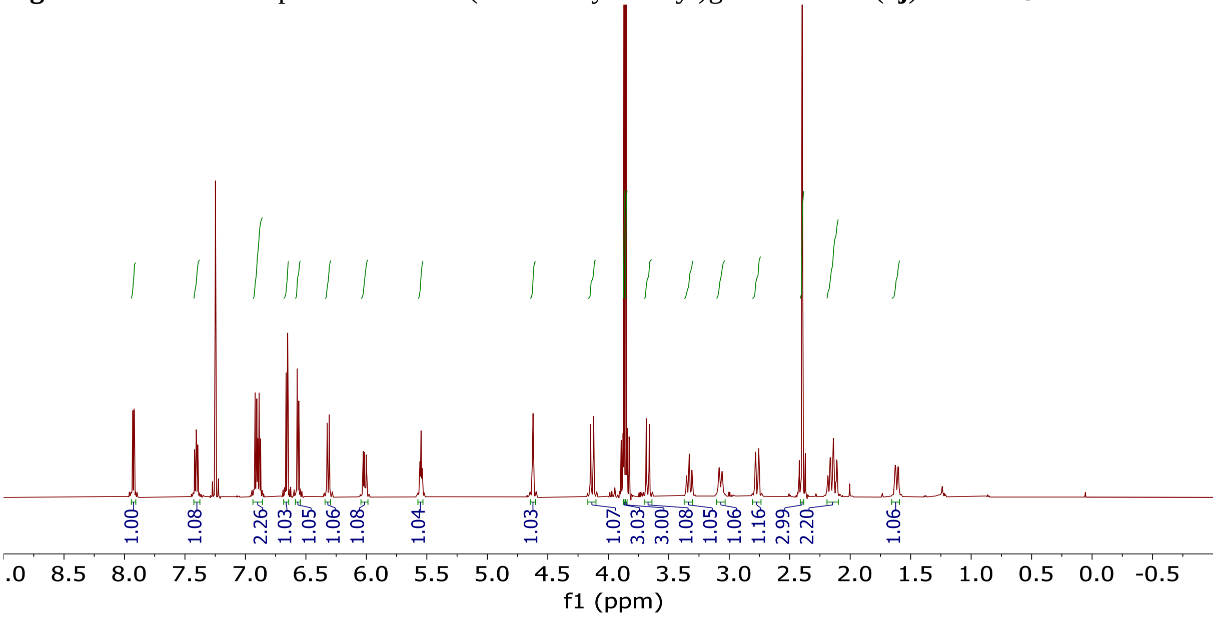

Figure S30. ^{13}C NMR spectrum of 6-*O*-(2-methoxybenzoyl)galanthamine (**1j**) in $CDCl_3$

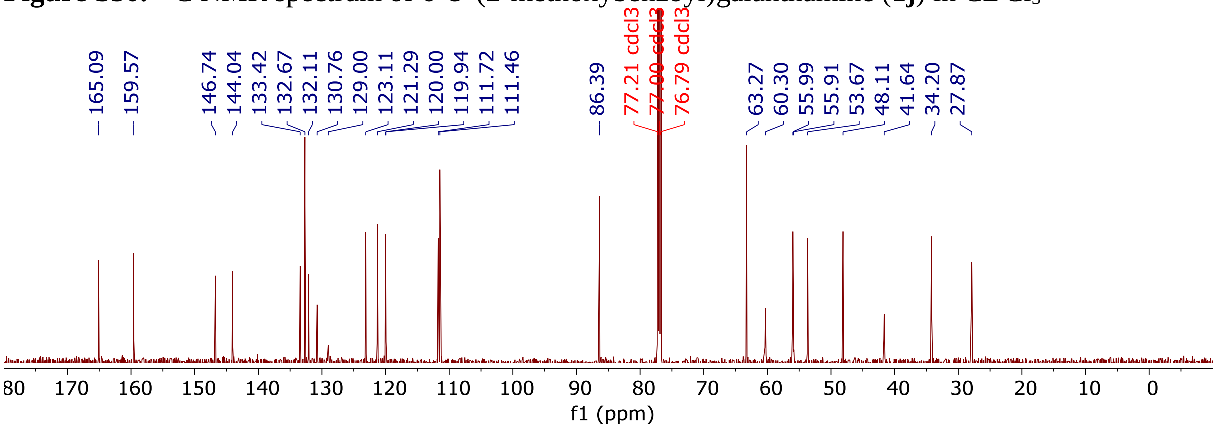

Figure S31. ESI-HRMS spectrum of 6-*O*-(3-methoxybenzoyl)galanthamine (**1k**)

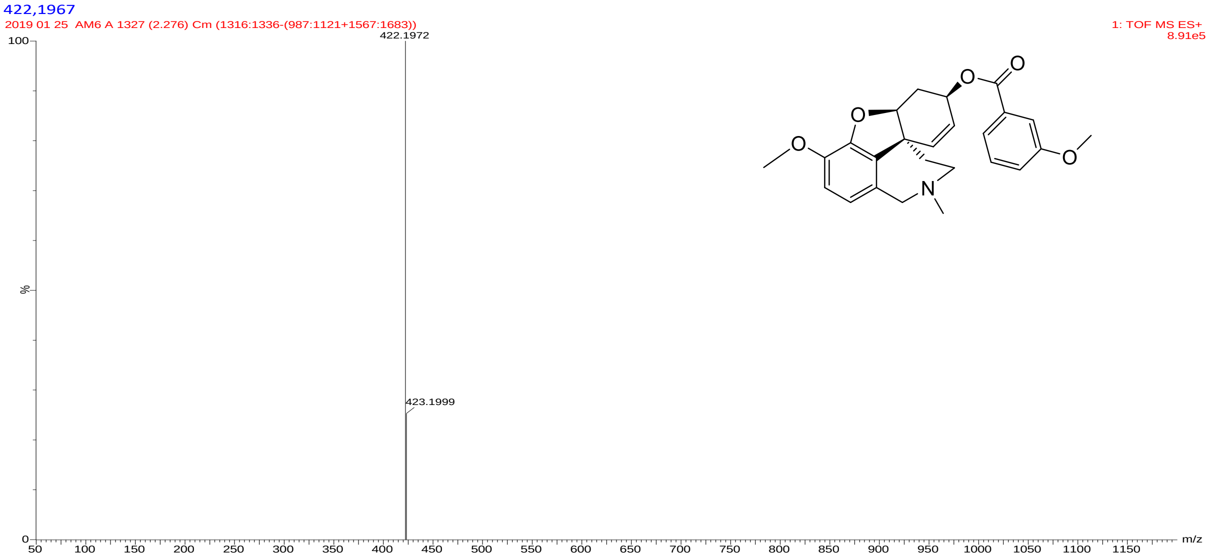

Figure S32. ^{1}H NMR spectrum of 6-*O*-(3-methoxybenzoyl)galanthamine (**1k**) in $CDCl_3$

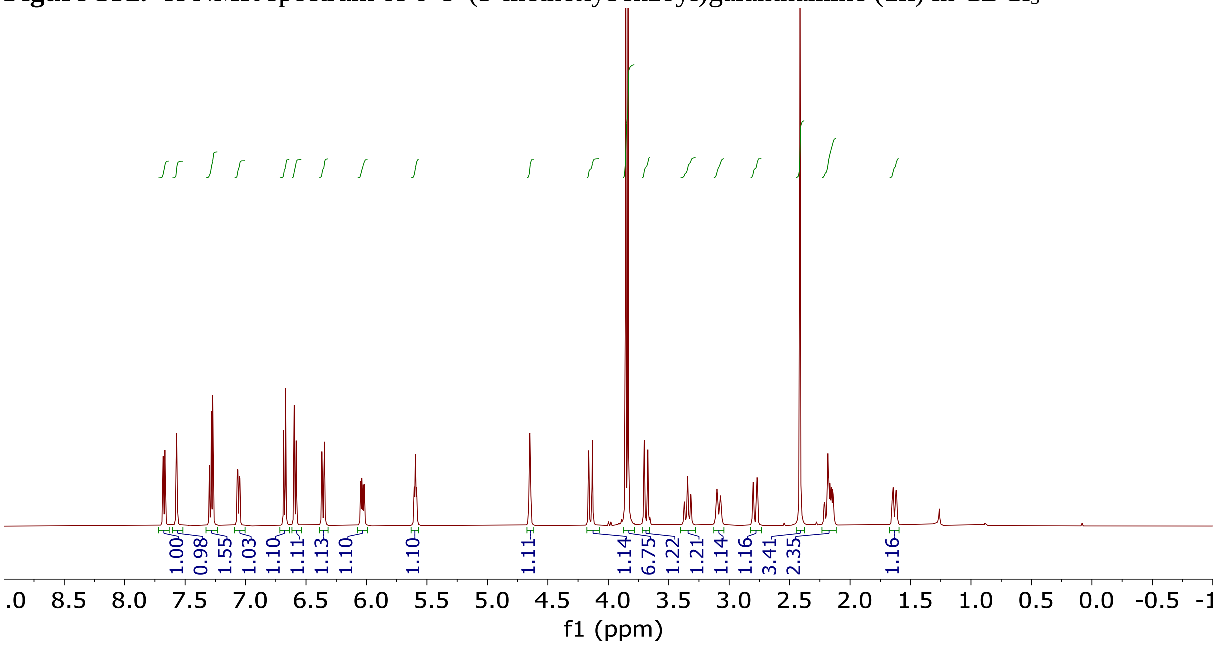

Figure S33. ^{13}C NMR spectrum of 6-*O*-(3-methoxybenzoyl)galanthamine (**1k**) in $CDCl_3$

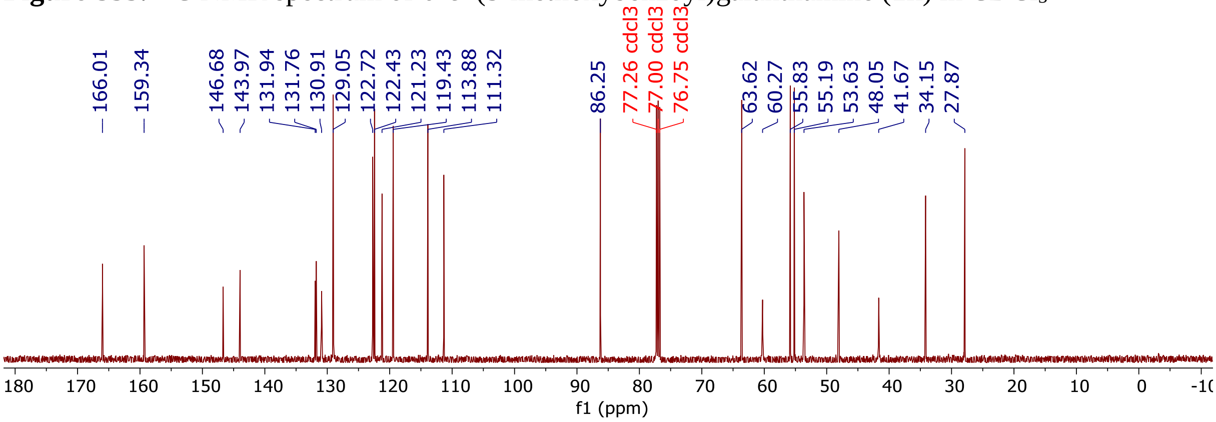

Figure S34. ESI-HRMS spectrum of 6-*O*-(4-methoxybenzoyl)galanthamine (**1l**)

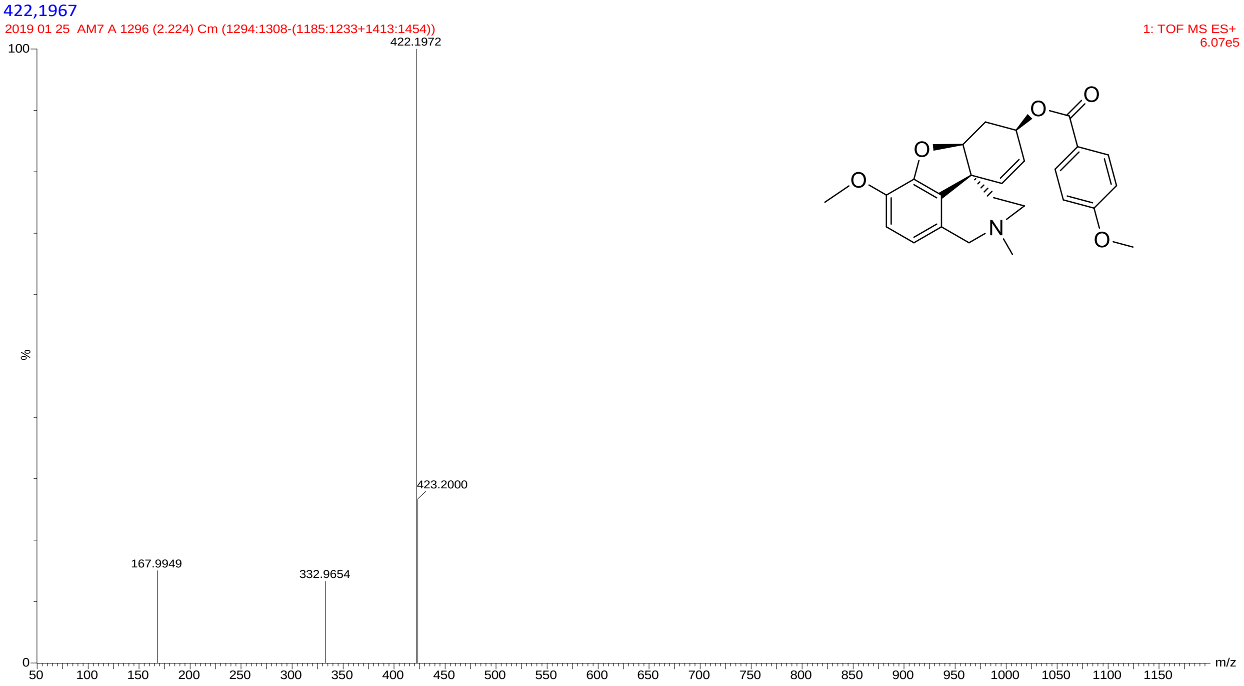

Figure S35. ^{1}H NMR spectrum of 6-*O*-(4-methoxybenzoyl)galanthamine (**1l**) in $CDCl_3$

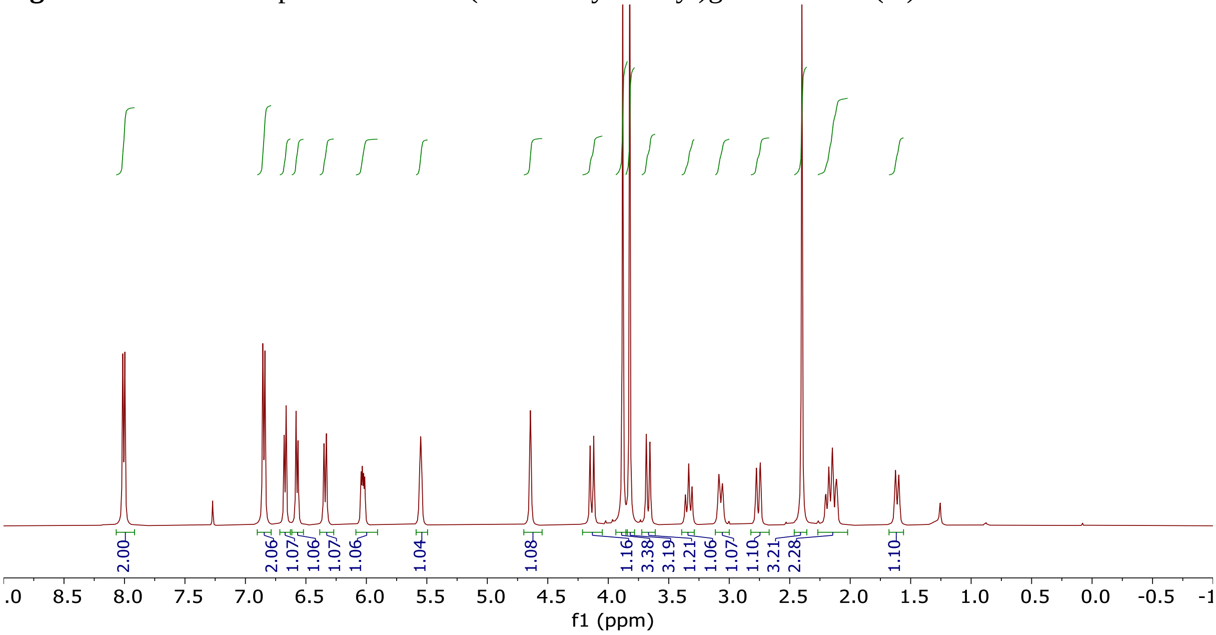

Figure S36. ^{13}C NMR spectrum of 6-*O*-(4-methoxybenzoyl)galanthamine (**1l**) in $CDCl_3$

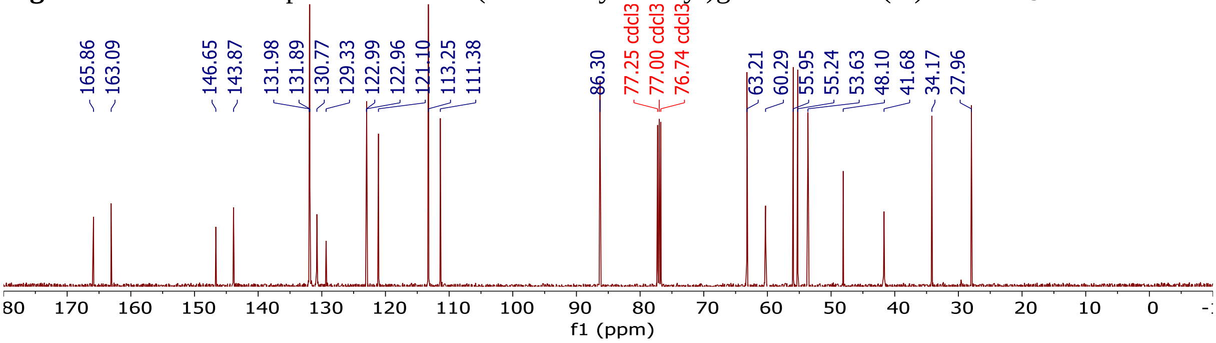

Figure S37. ESI-HRMS spectrum of 6-*O*-(3,4-dimethoxybenzoyl)galanthamine (**1m**)

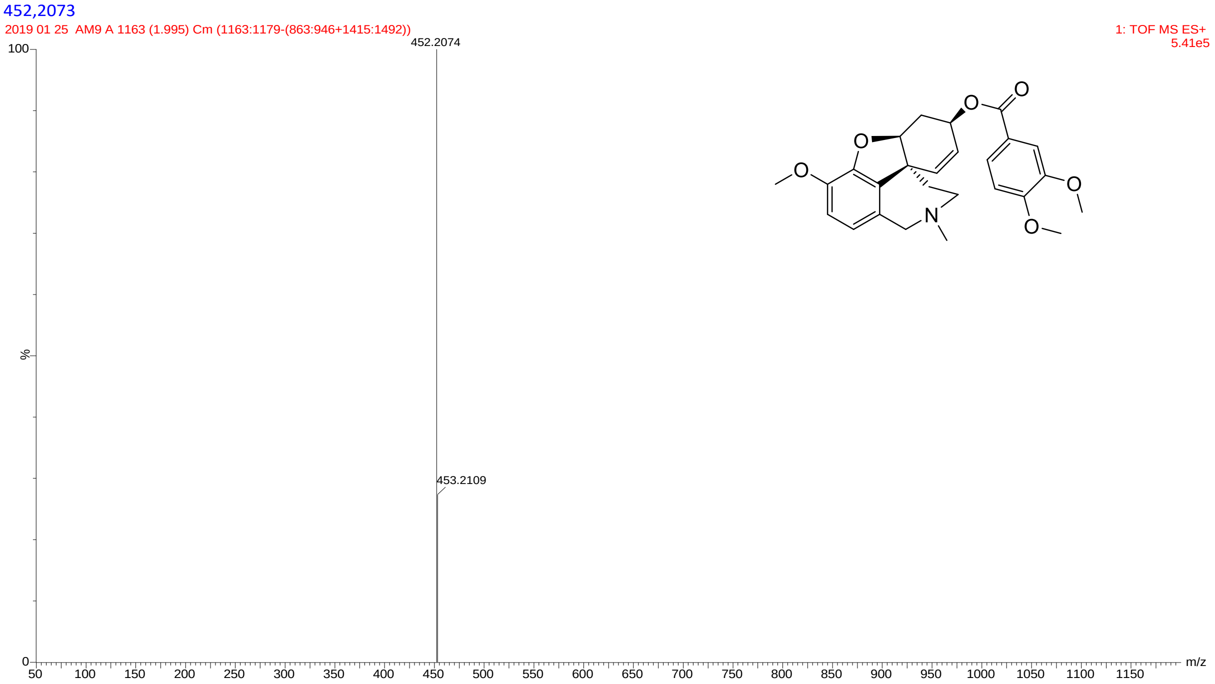

Figure S38. ^{1}H NMR spectrum of 6-*O*-(3,4-dimethoxybenzoyl)galanthamine (**1m**) in $CDCl_3$

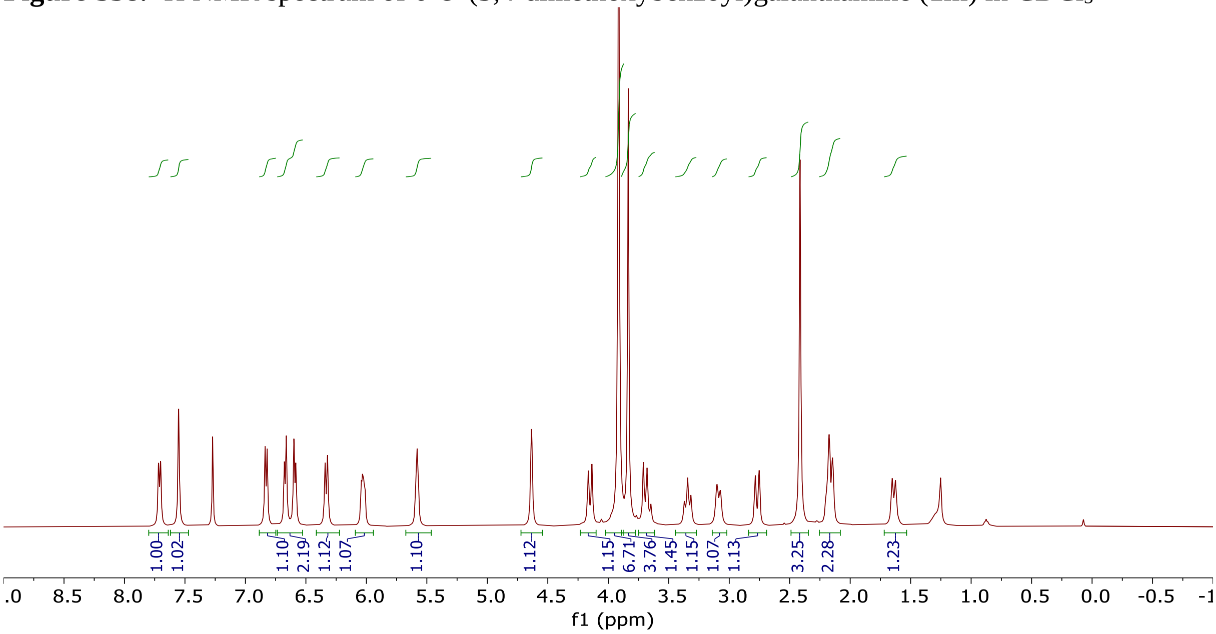

Figure S39. ^{13}C NMR spectrum of 6-*O*-(3,4-dimethoxybenzoyl)galanthamine (**1m**) in $CDCl_3$

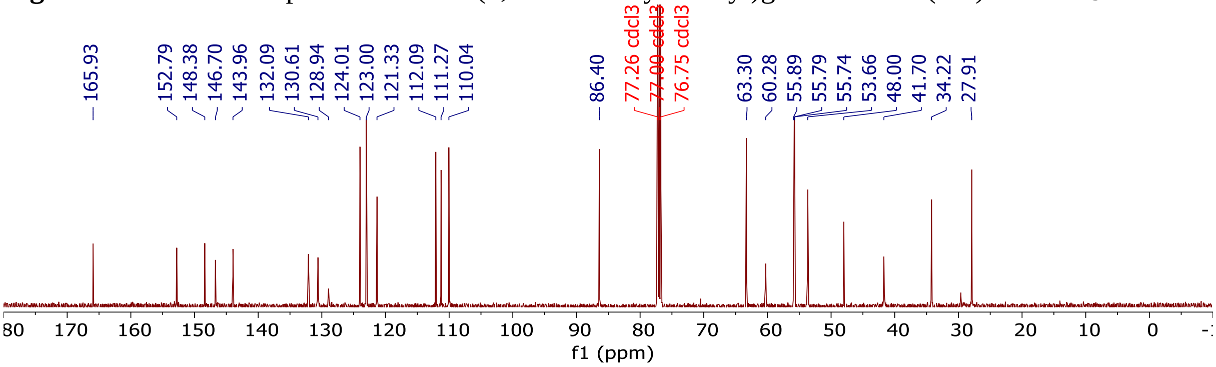

Figure S40. ESI-HRMS spectrum of 6-*O*-(3,5-dimethoxybenzoyl)galanthamine (**1n**)

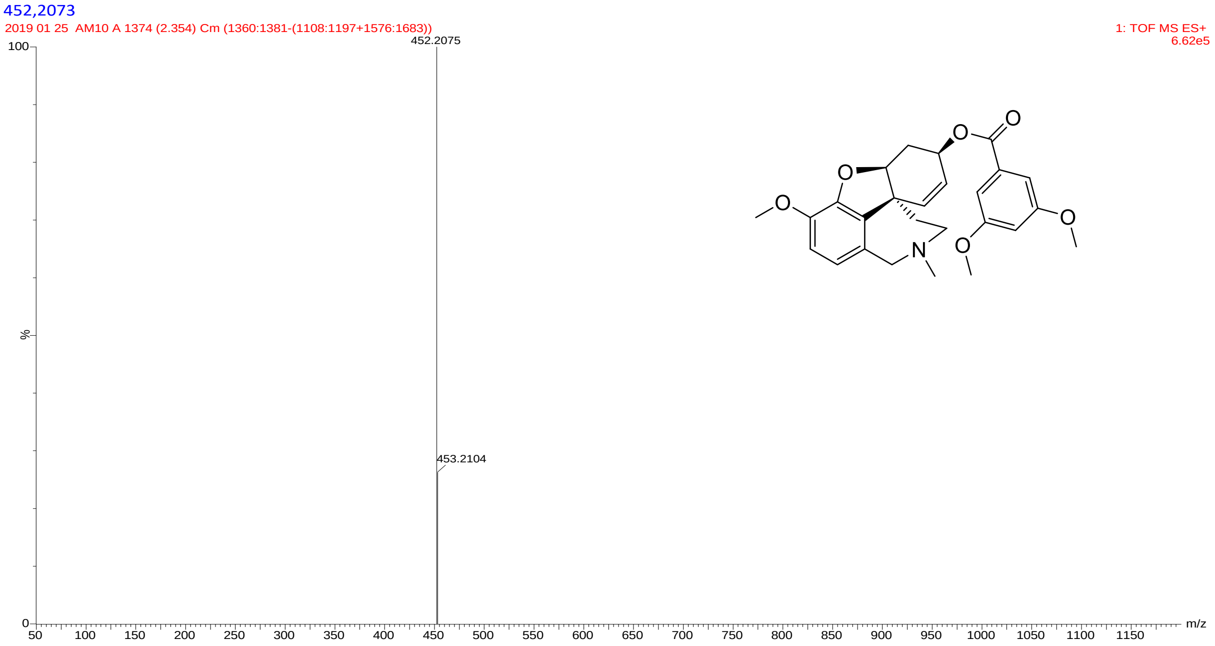

Figure S41. ^{1}H NMR spectrum of 6-*O*-(3,5-dimethoxybenzoyl)galanthamine (**1n**) in $CDCl_3$

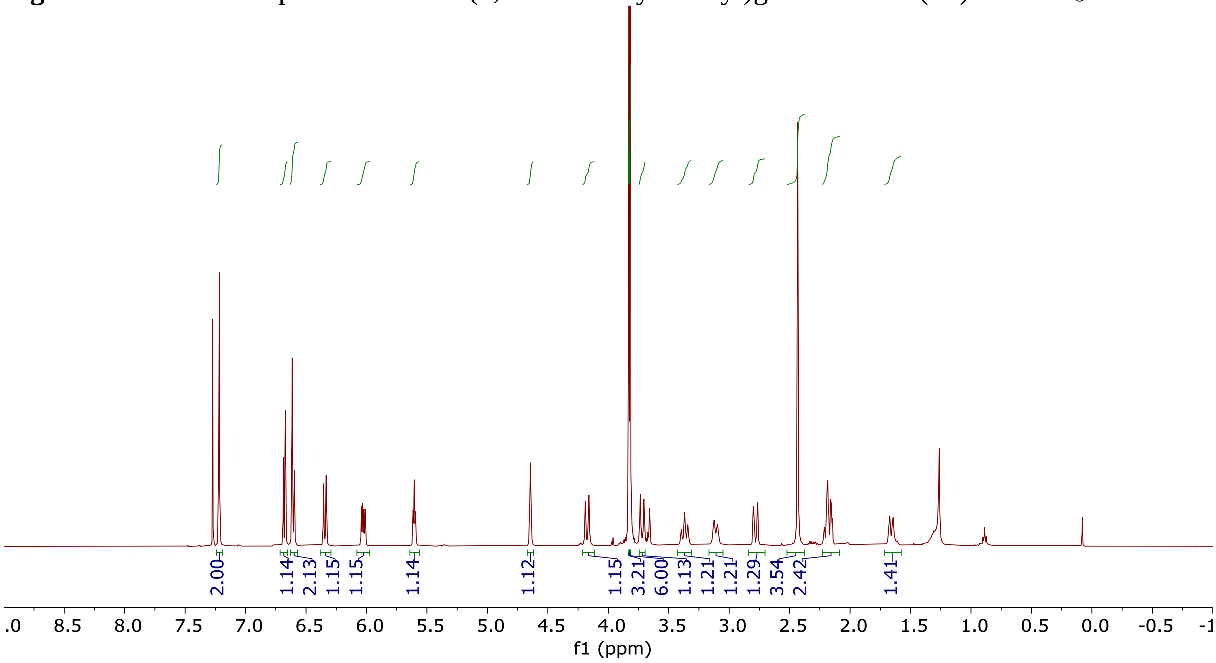

Figure S42. ^{13}C NMR spectrum of 6-*O*-(3,5-dimethoxybenzoyl)galanthamine (**1n**) in $CDCl_3$

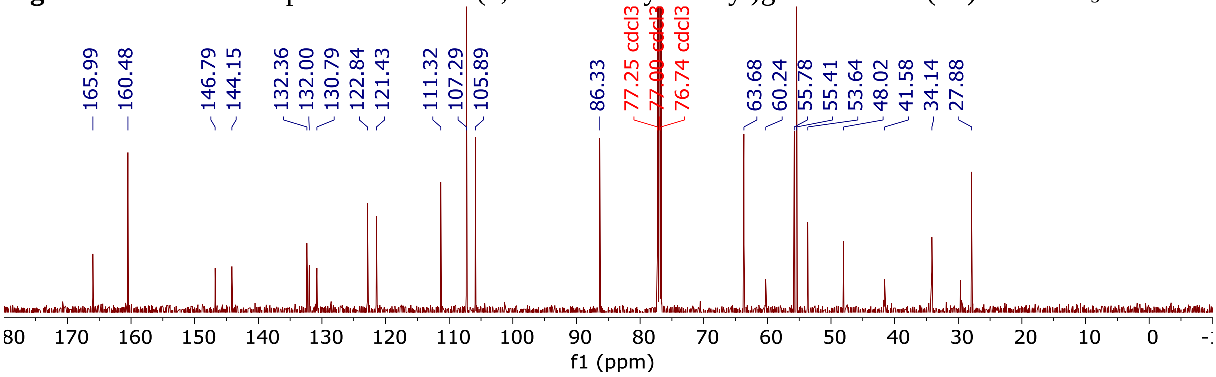

Figure S43. ESI-HRMS spectrum of 6-*O*-(3,4,5-trimethoxybenzoyl)galanthamine (**1o**)

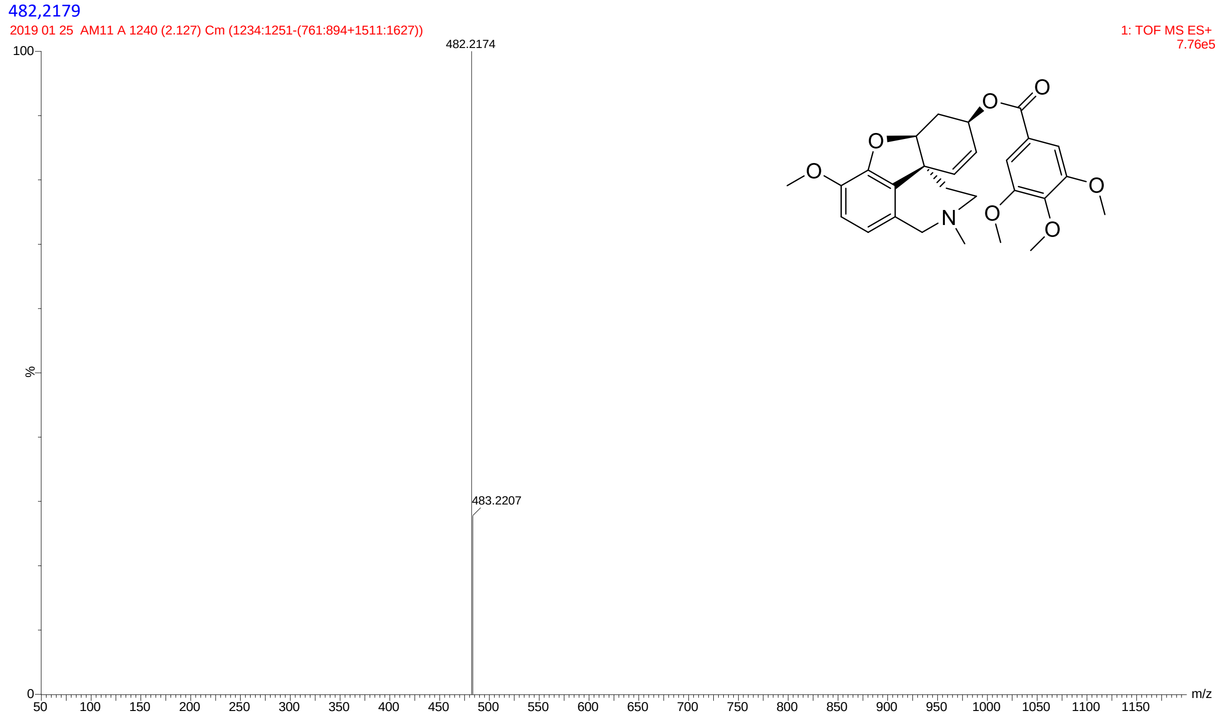

Figure S44. ^{1}H NMR spectrum of 6-*O*-(3,4,5-trimethoxybenzoyl)galanthamine (**1o**) in $CDCl_3$

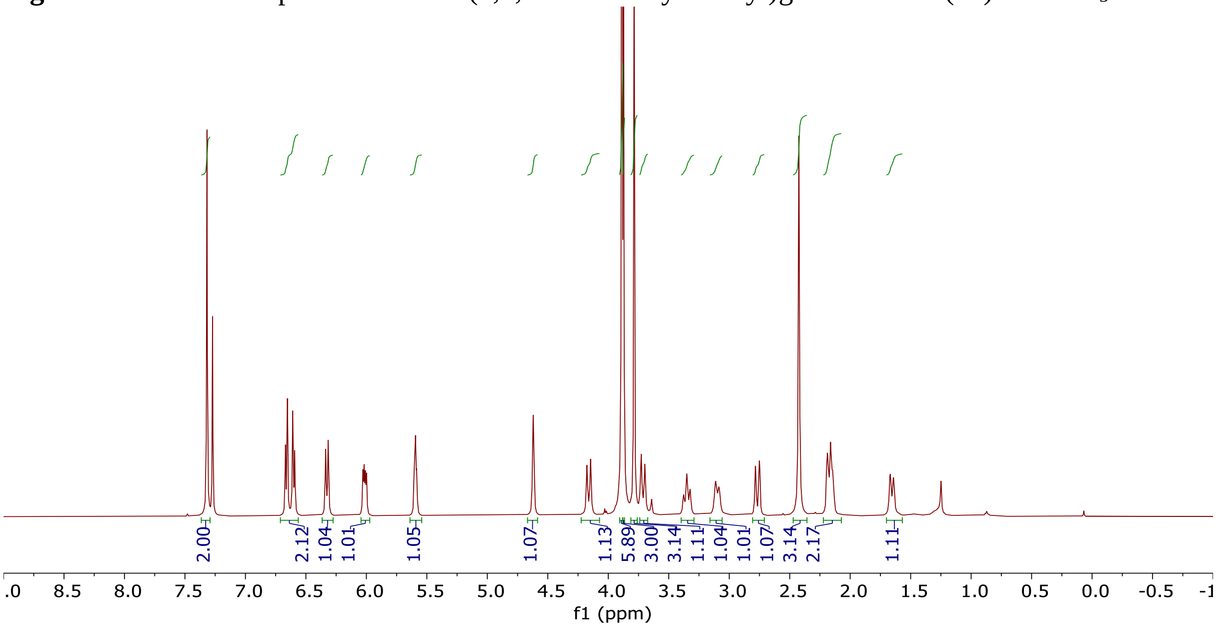

Figure S45. ^{13}C NMR spectrum of 6-*O*-(3,4,5-trimethoxybenzoyl)galanthamine (**1o**) in $CDCl_3$

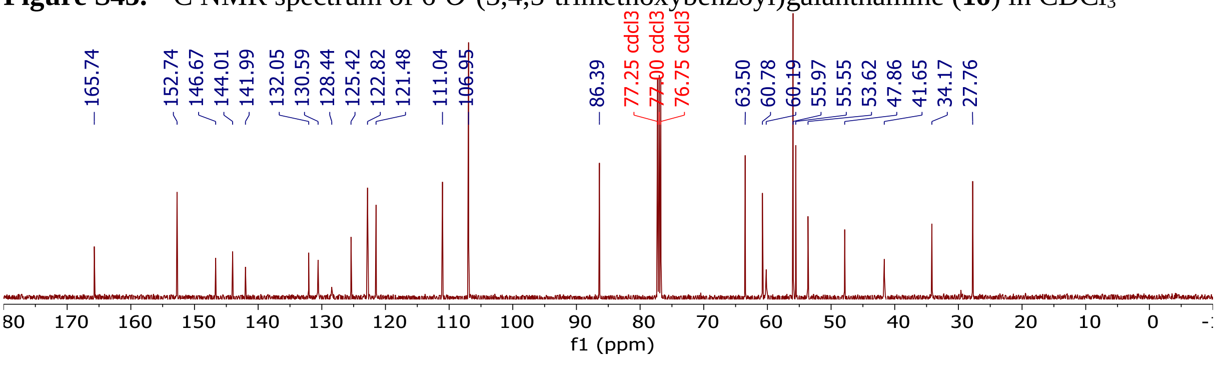

Figure S46. ESI-HRMS spectrum of 6-*O*-(3,5-diethoxybenzoyl)galanthamine (**1p**)

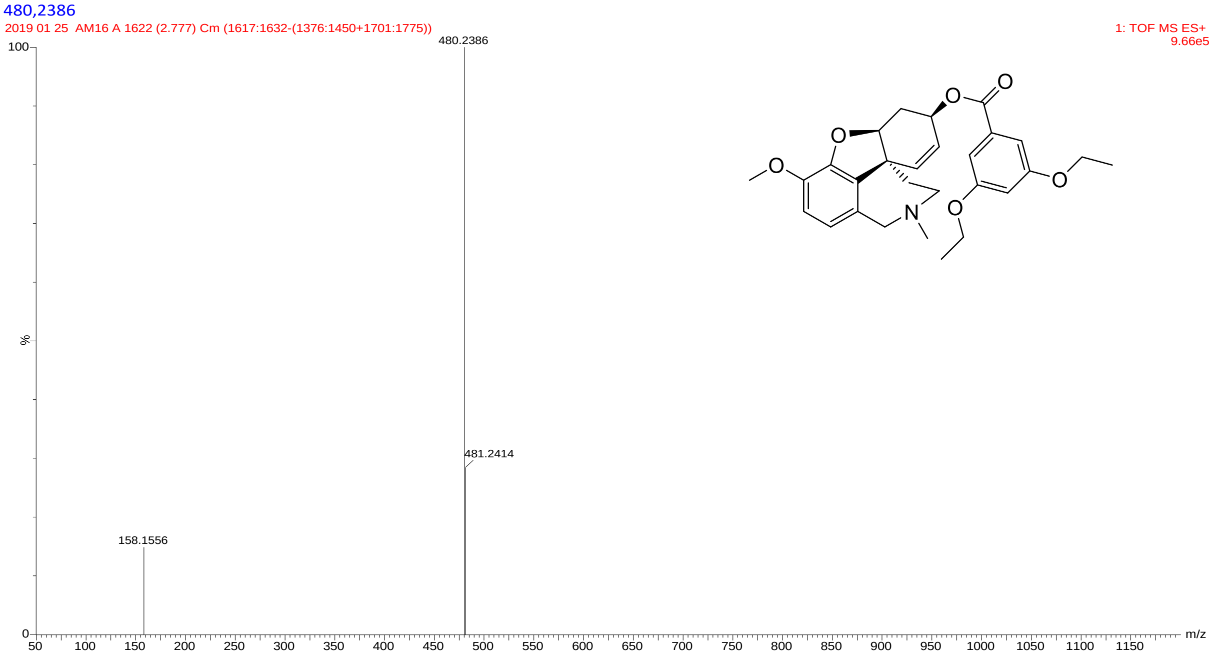

Figure S47. ^{1}H NMR spectrum of 6-*O*-(3,5-diethoxybenzoyl)galanthamine (**1p**) in $CDCl_3$

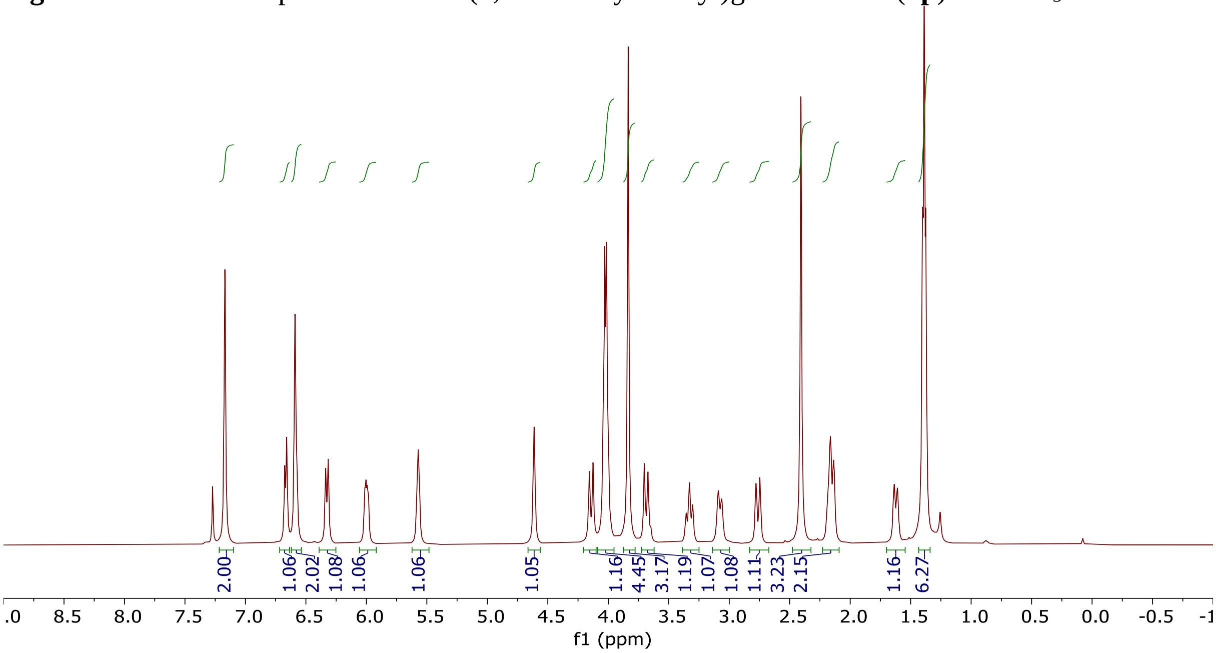

Figure S48. ^{13}C NMR spectrum of 6-*O*-(3,5-diethoxybenzoyl)galanthamine (**1p**) in $CDCl_3$

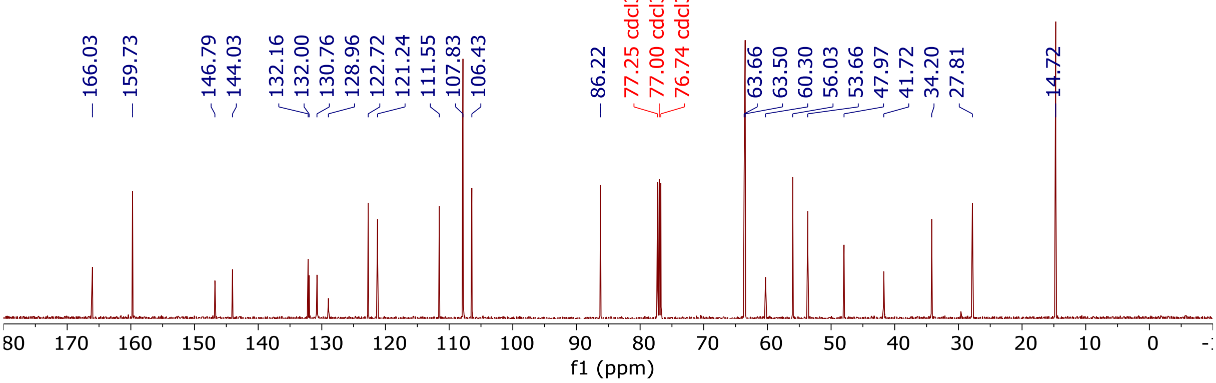

Figure S49. ESI-HRMS spectrum of 6-*O*-(1-naphtoyl)galanthamine (**1q**)

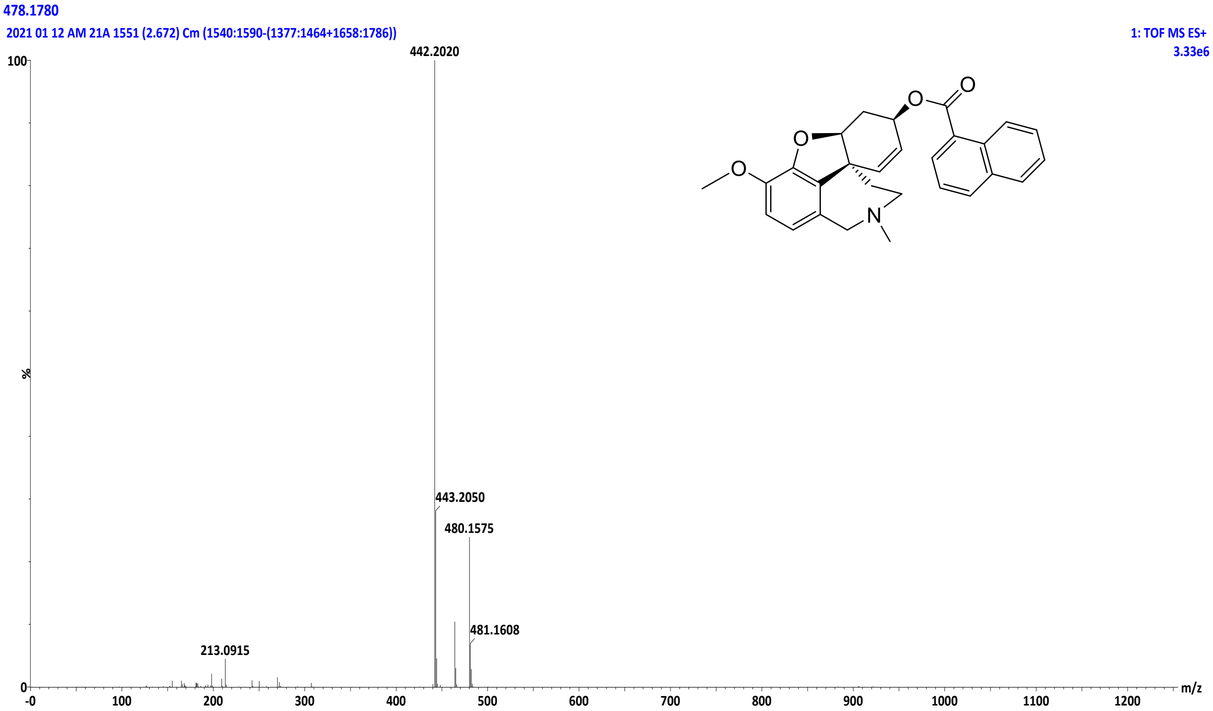

Figure S50. ^{1}H NMR spectrum of 6-*O*-(1-naphtoyl)galanthamine (**1q**) in $CDCl_3$

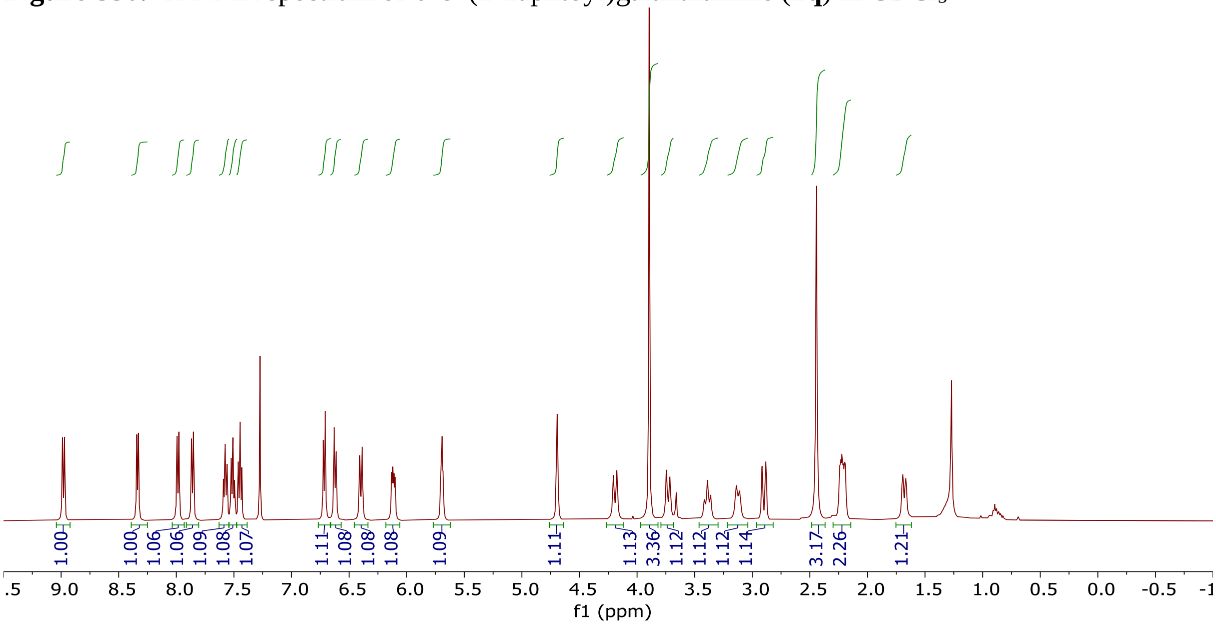

Figure S51. ^{13}C NMR spectrum of 6-*O*-(1-naphtoyl)galanthamine (**1q**) in $CDCl_3$

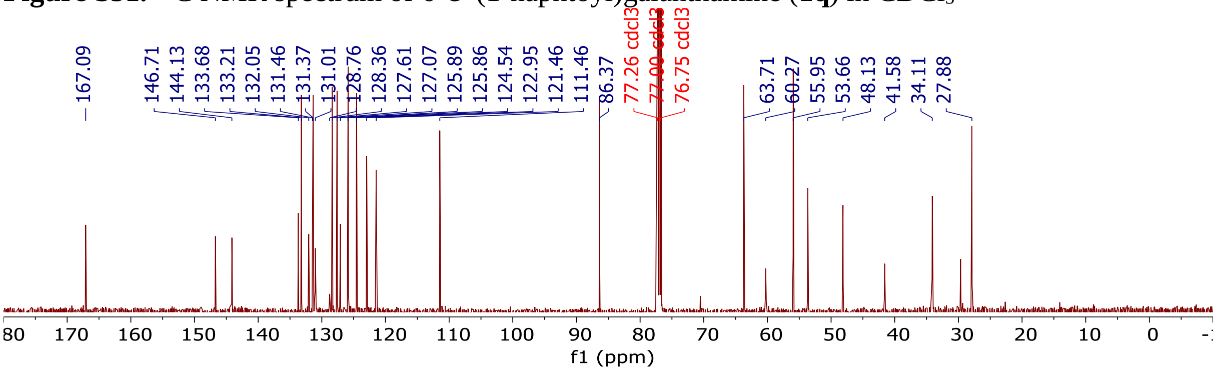

Figure S52. ESI-HRMS spectrum of 6-*O*-(2-naphtoyl)galanthamine (**1r**)

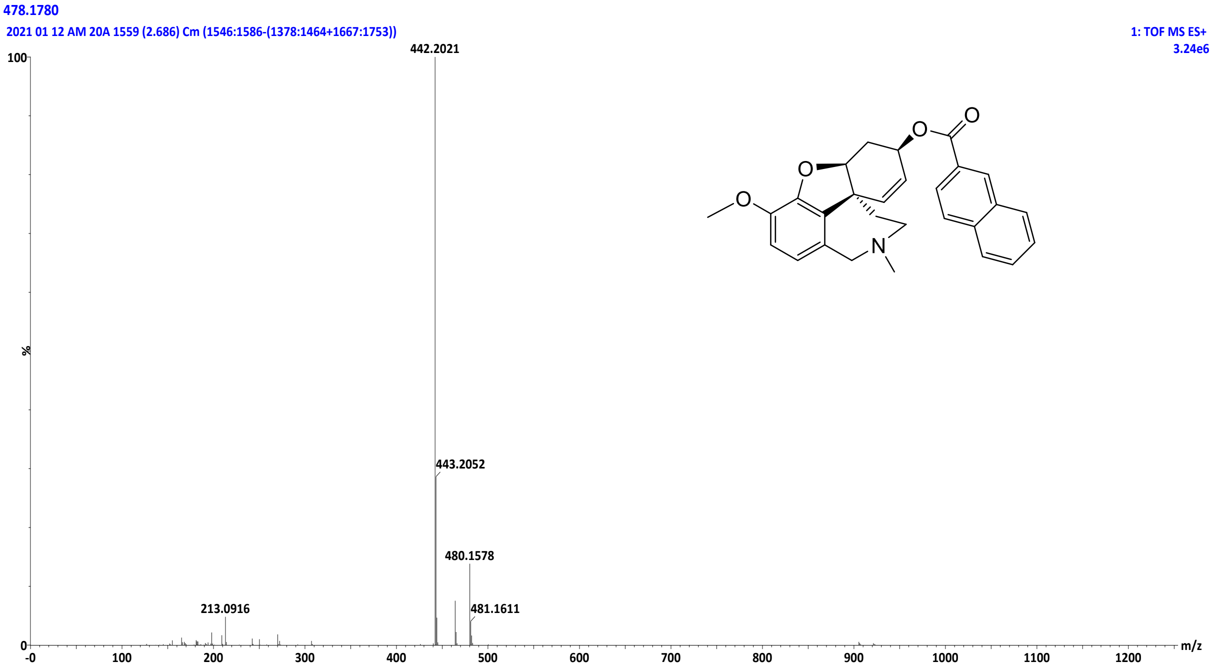

Figure S53. ^{1}H NMR spectrum of 6-*O*-(2-naphtoyl)galanthamine (**1r**) in $CDCl_3$

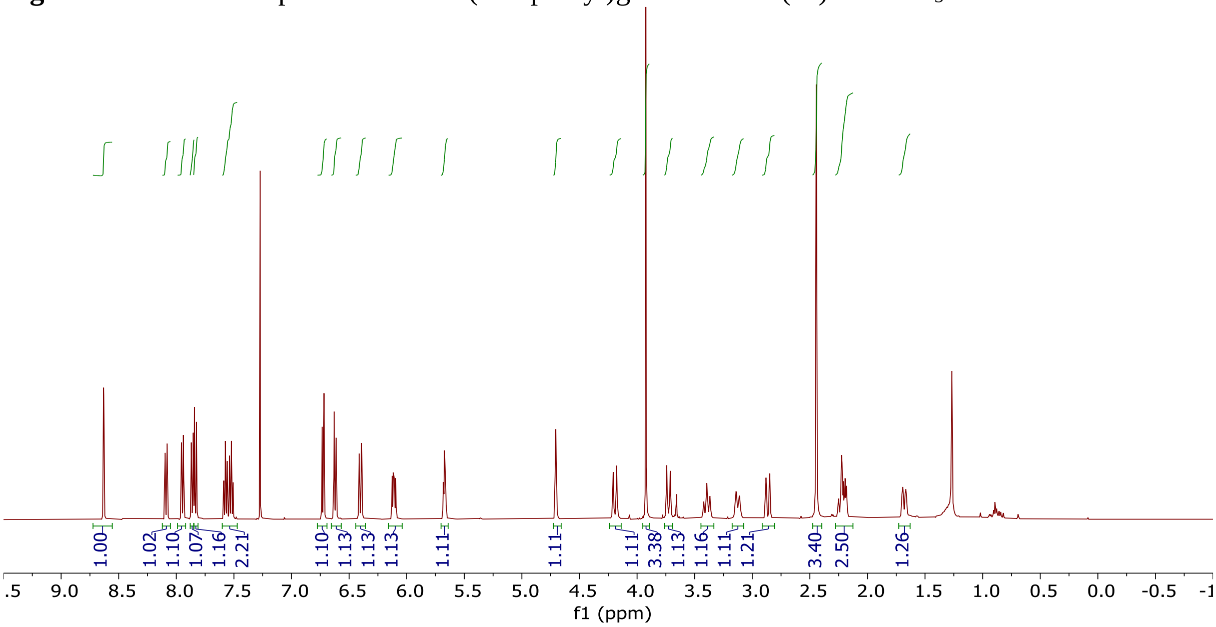

Figure S54. ^{13}C NMR spectrum of 6-*O*-(2-naphtoyl)galanthamine (**1r**) in $CDCl_3$

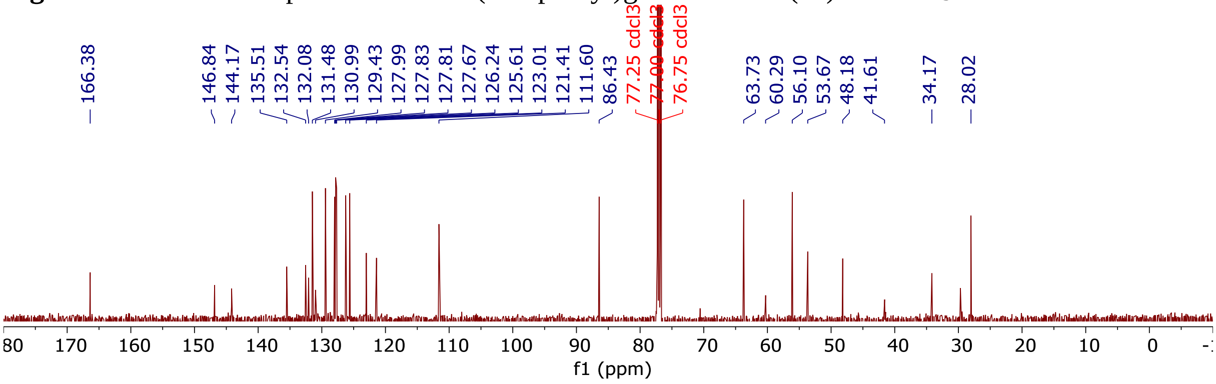

Figure S55. ESI-HRMS spectrum of 6-*O*-(2-furoyl)galanthamine (**1s**)

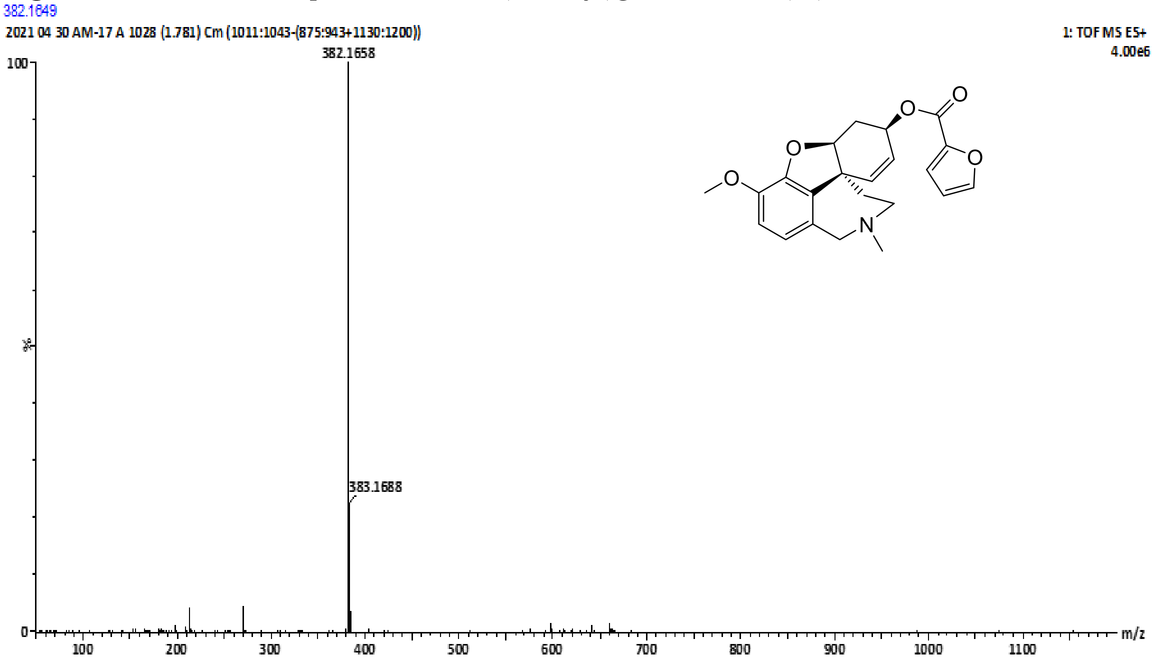

Figure S56. ^{1}H NMR spectrum of 6-*O*-(2-furoyl)galanthamine (**1s**) in $CDCl_3$

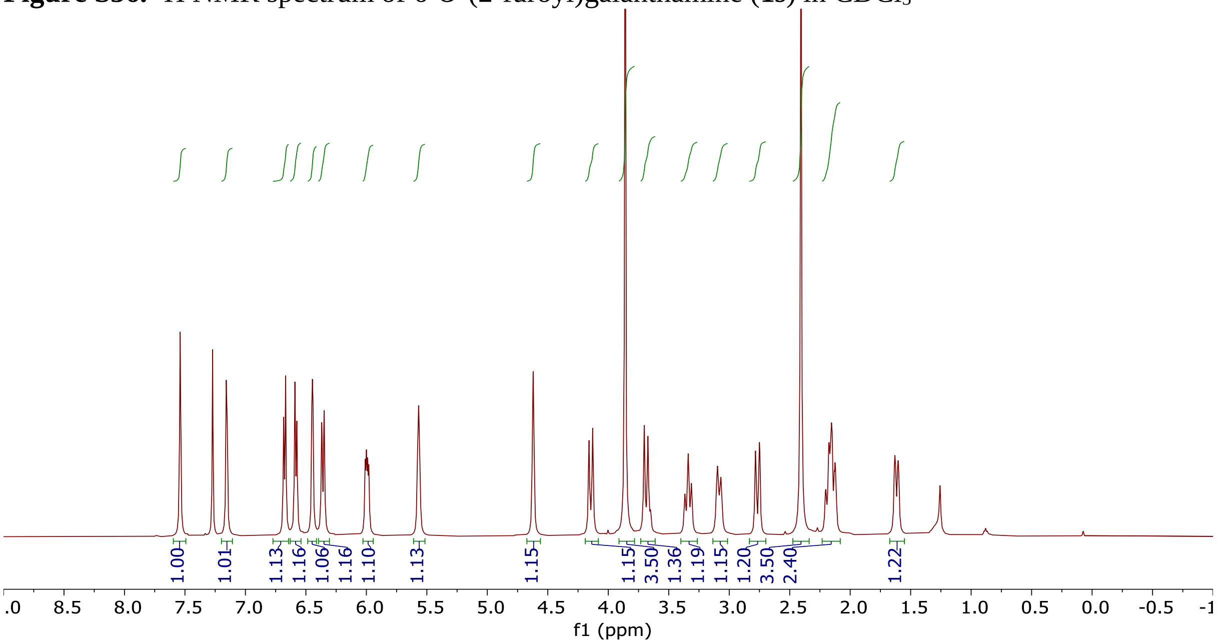

Figure S57. ^{13}C NMR spectrum of 6-*O*-(2-furoyl)galanthamine (**1s**) in $CDCl_3$

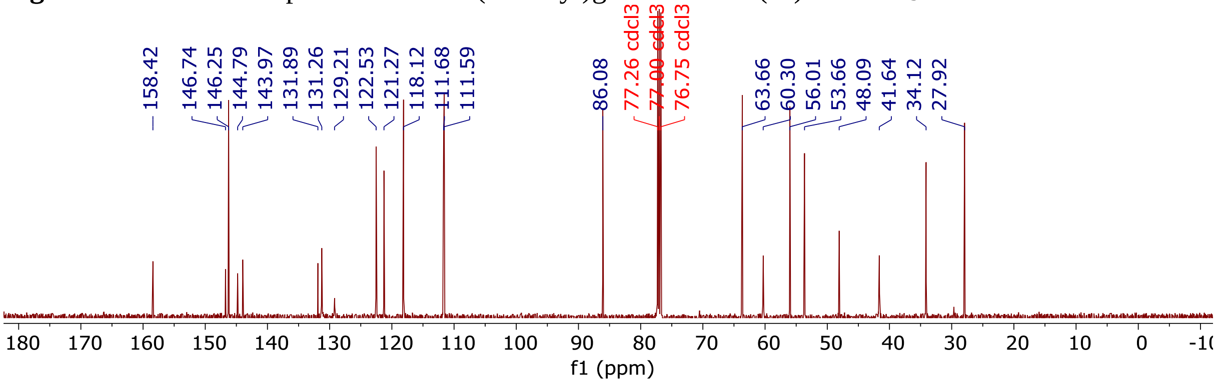

Figure S58. ESI-HRMS spectrum of 3-*O*-methylpancracine (**3**)

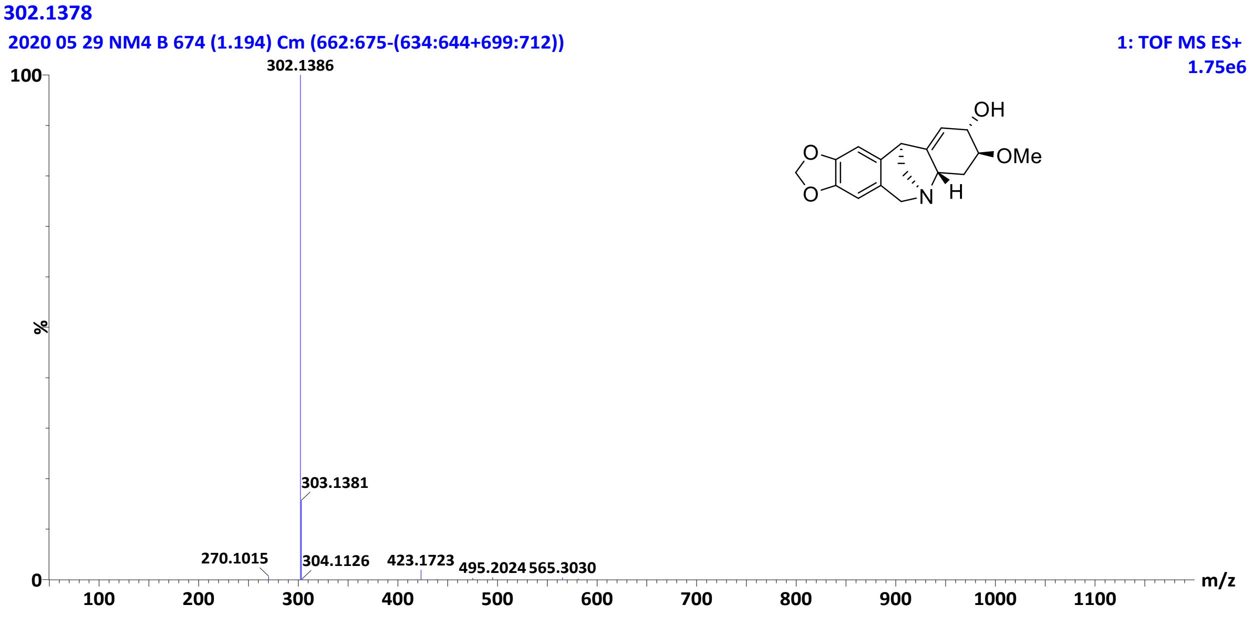

Figure S59. ^{1}H NMR spectrum of 3-*O*-methylpancracine (**3**) in $CDCl_3$

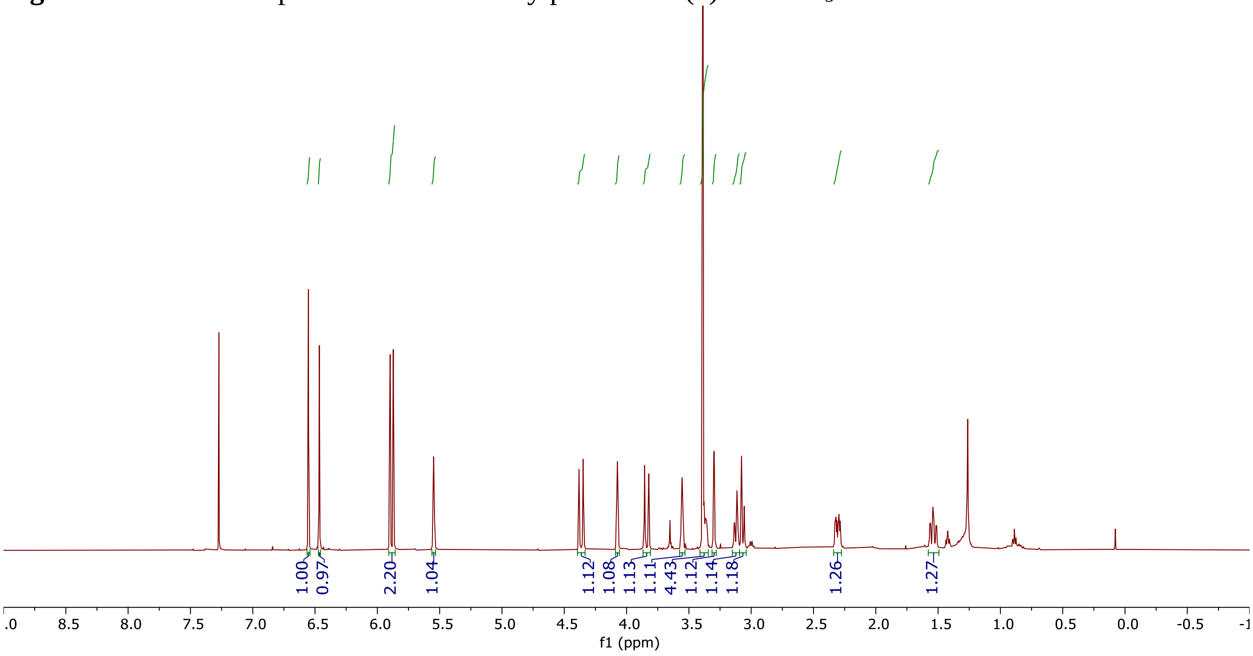

Figure S60. ^{13}C NMR spectrum of 3-*O*-methylpancracine (**3**) in $CDCl_3$

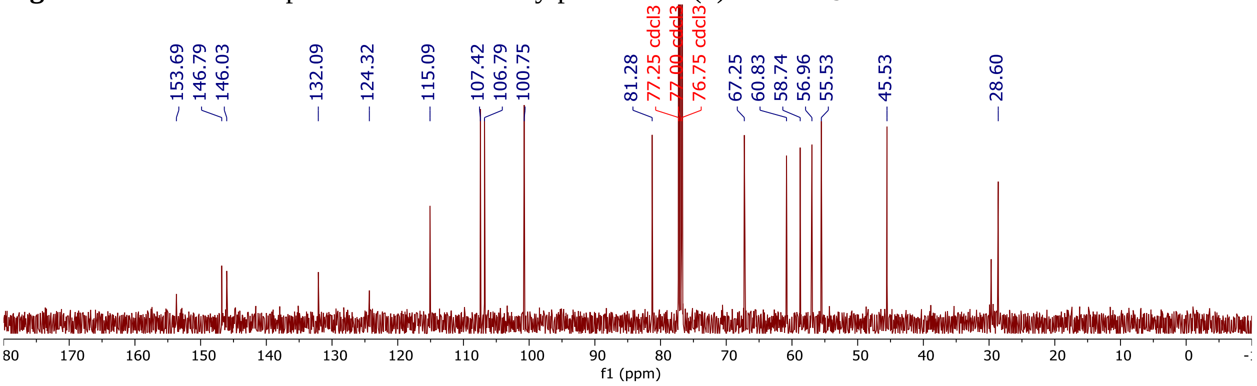

Figure S61. ESI-HRMS spectrum of 2-*O*-(3,5-dimethylbenzoyl)-3-*O*-methylpancracine (**3a**)

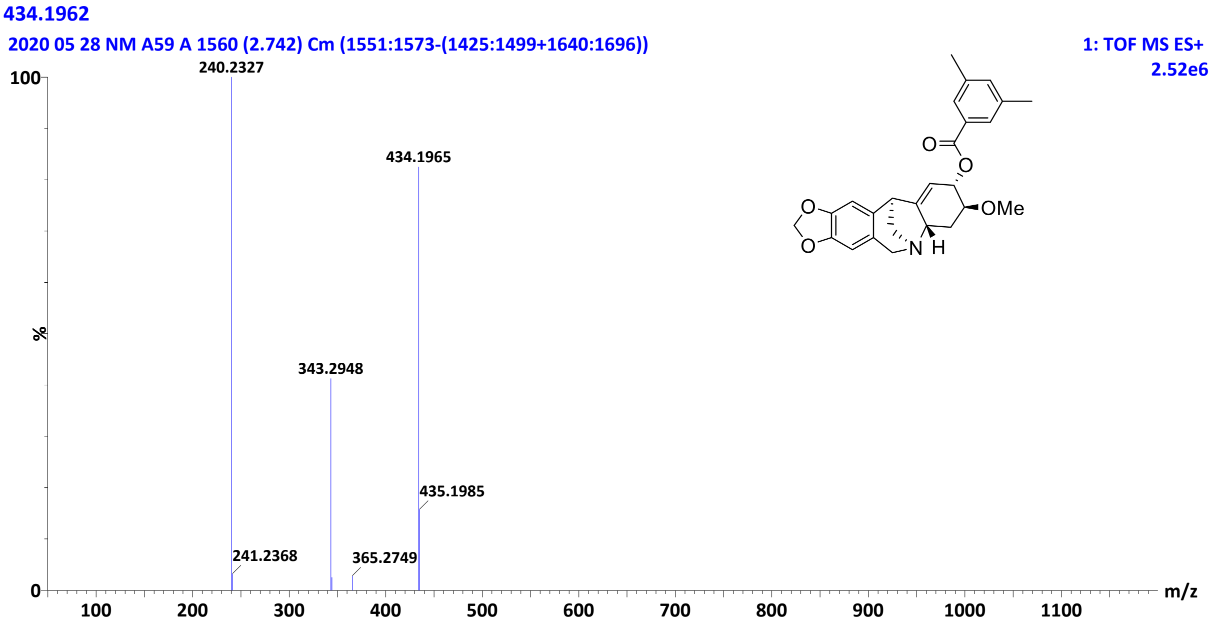

Figure S62. ^{1}H NMR spectrum of 2-*O*-(3,5-dimethylbenzoyl)-3-*O*-methylpancracine (**3a**) in $CDCl_3$

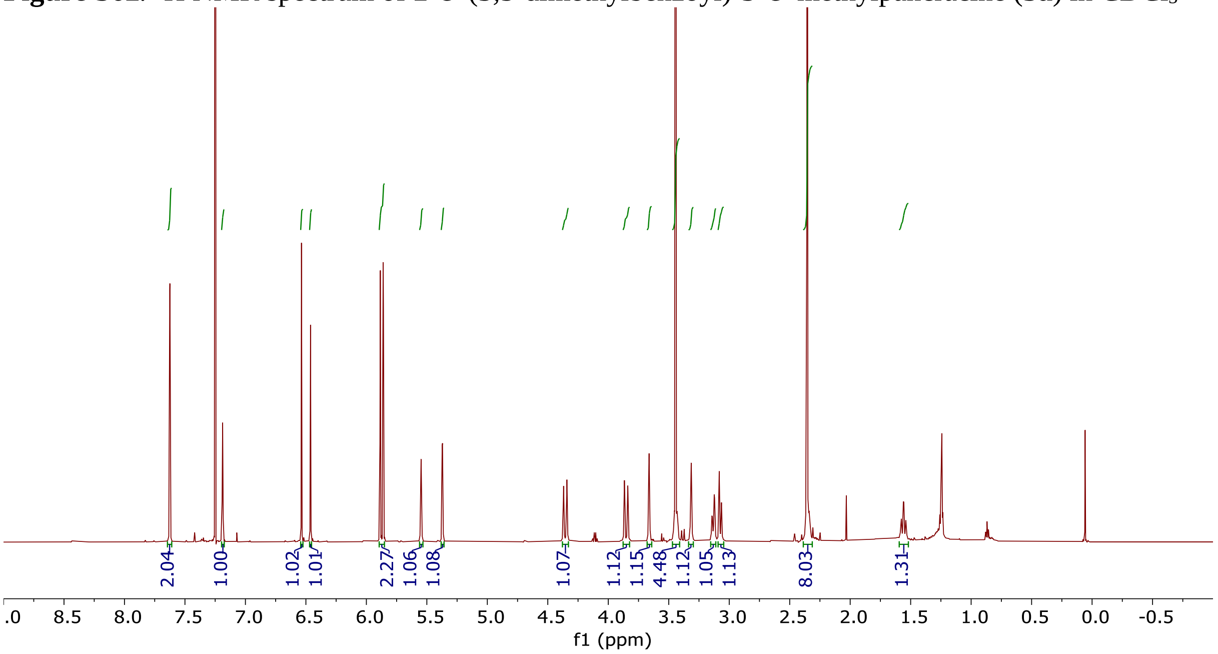

Figure S63. ^{13}C NMR spectrum of 2-*O*-(3,5-dimethylbenzoyl)-3-*O*-methylpancracine (**3a**) in $CDCl_3$

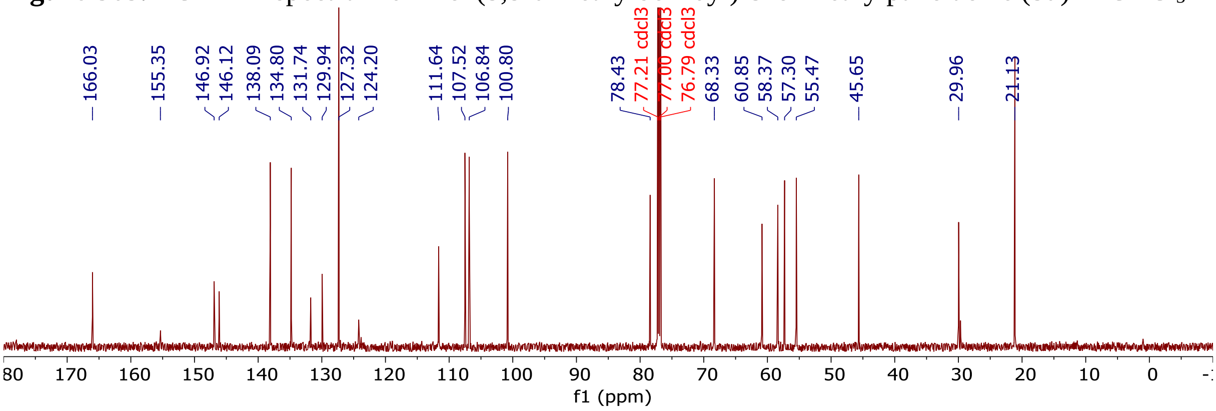

Figure S64. ESI-HRMS spectrum of 2-*O*-(2,4,6-trimethylbenzoyl)-3-*O*-methylpancracine (**3b**)

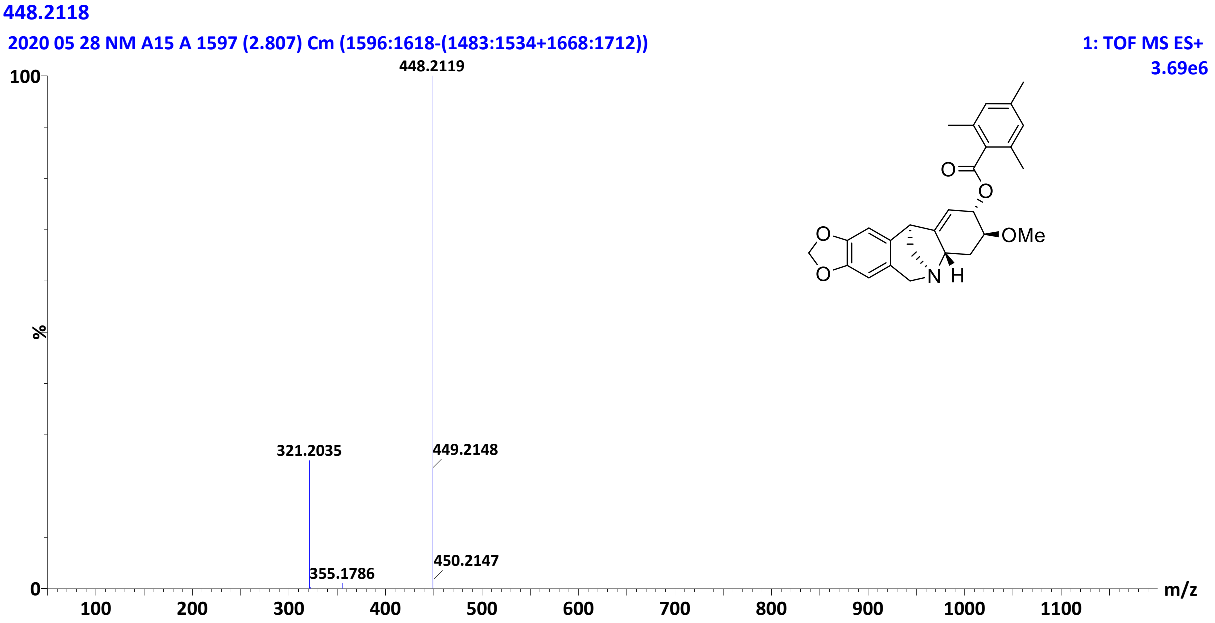

Figure S65. ^{1}H NMR spectrum of 2-*O*-(2,4,6-trimethylbenzoyl)-3-*O*-methylpancracine (**3b**) in $CDCl_3$

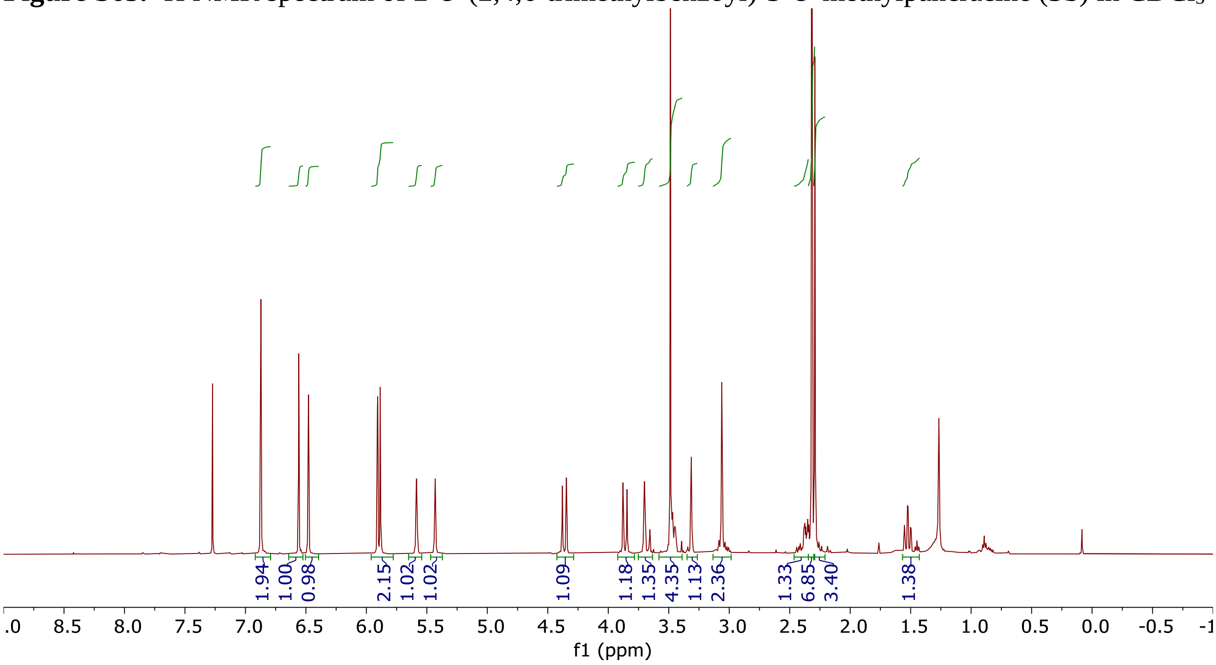

Figure S66. ^{13}C NMR spectrum of 2-*O*-(2,4,6-trimethylbenzoyl)-3-*O*-methylpancracine (**3b**) in $CDCl_3$

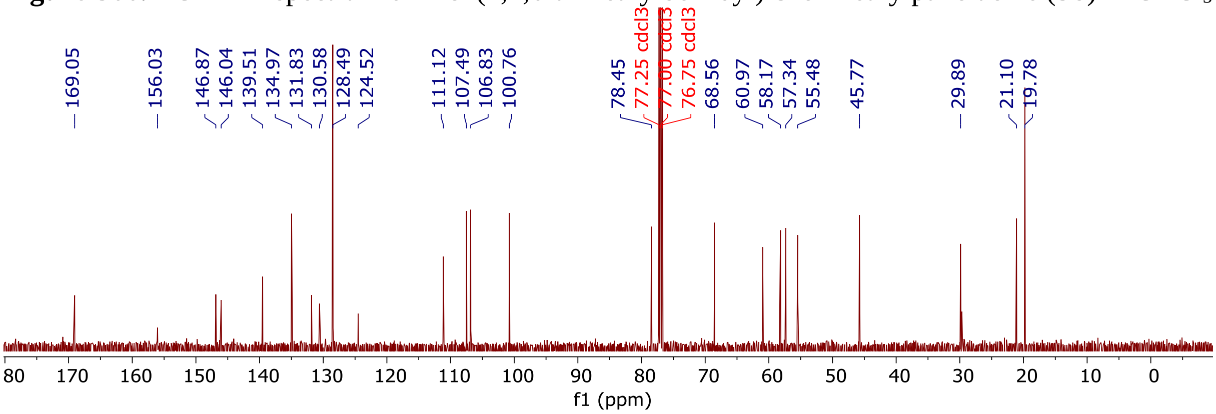

Figure S67. ESI-HRMS spectrum of 2-*O*-(4-tert-butylbenzoyl)-3-*O*-methylpancracine (**3c**)

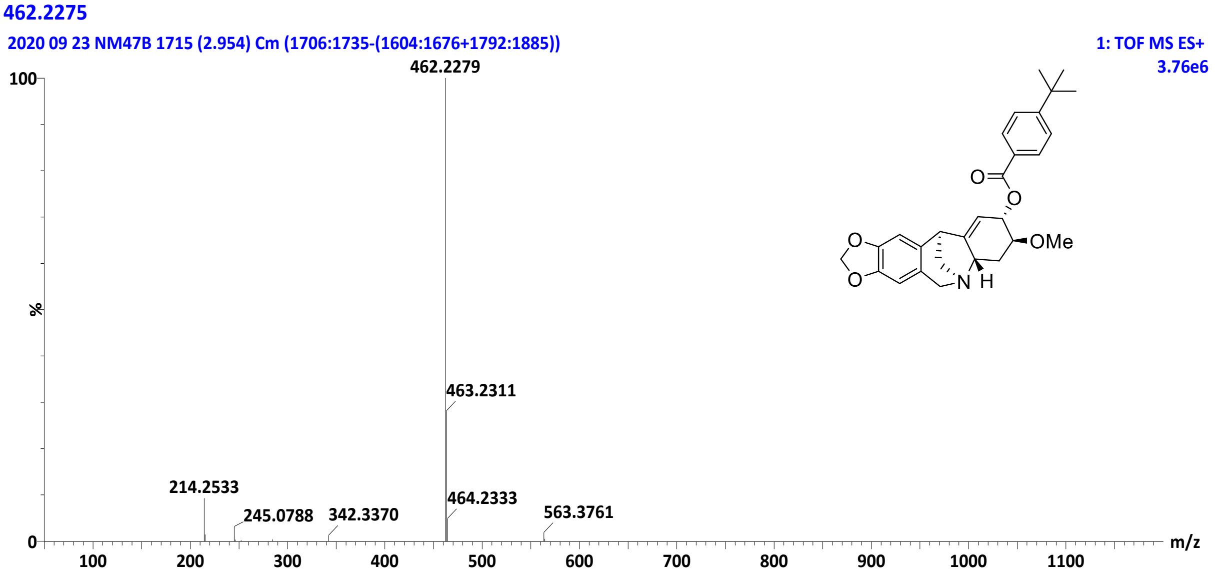

Figure S68. ^{1}H NMR spectrum of 2-*O*-(4-tert-butylbenzoyl)-3-*O*-methylpancracine (**3c**) in $CDCl_3$

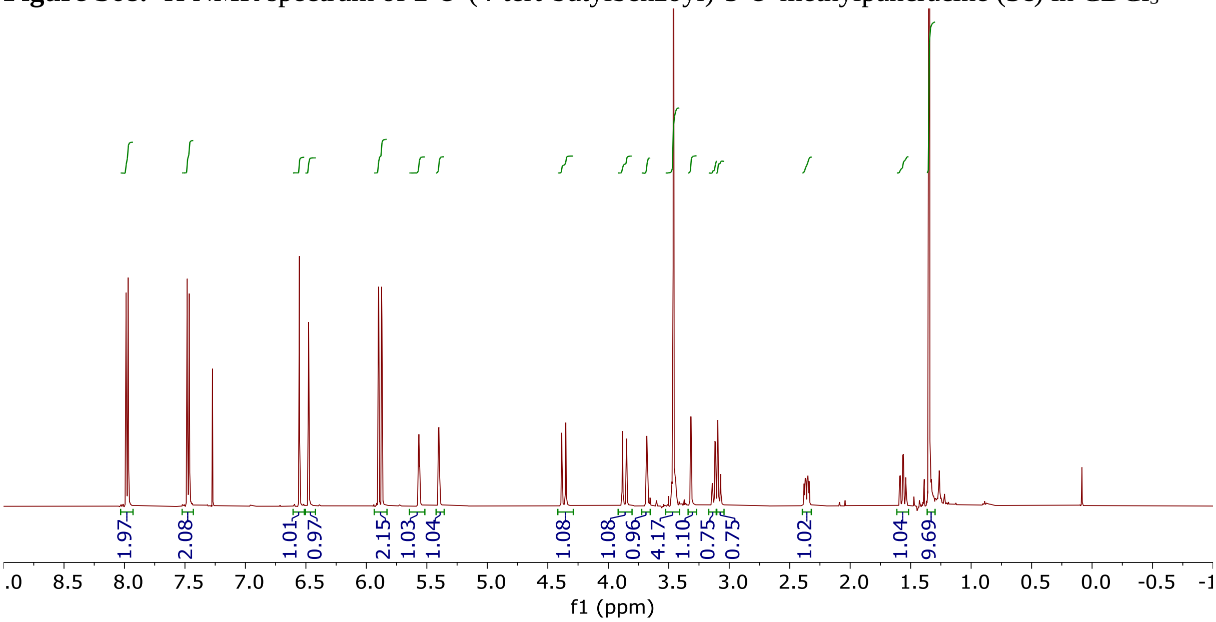

Figure S69. ^{13}C NMR spectrum of 2-*O*-(4-tert-butylbenzoyl)-3-*O*-methylpancracine (**3c**) in $CDCl_3$

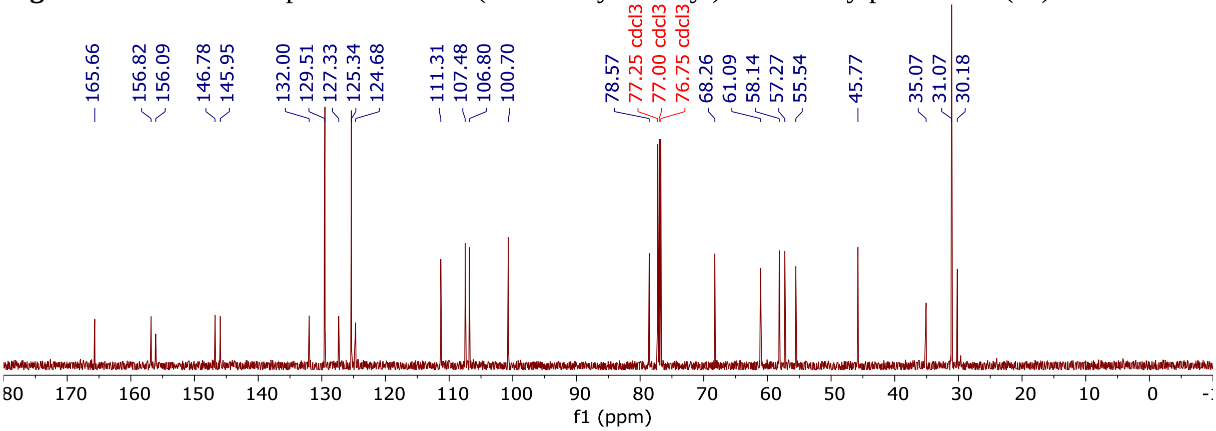

Figure S70. ESI-HRMS spectrum of 2-*O*-(4-butylbenzoyl)-3-*O*-methylpancracine (**3d**)

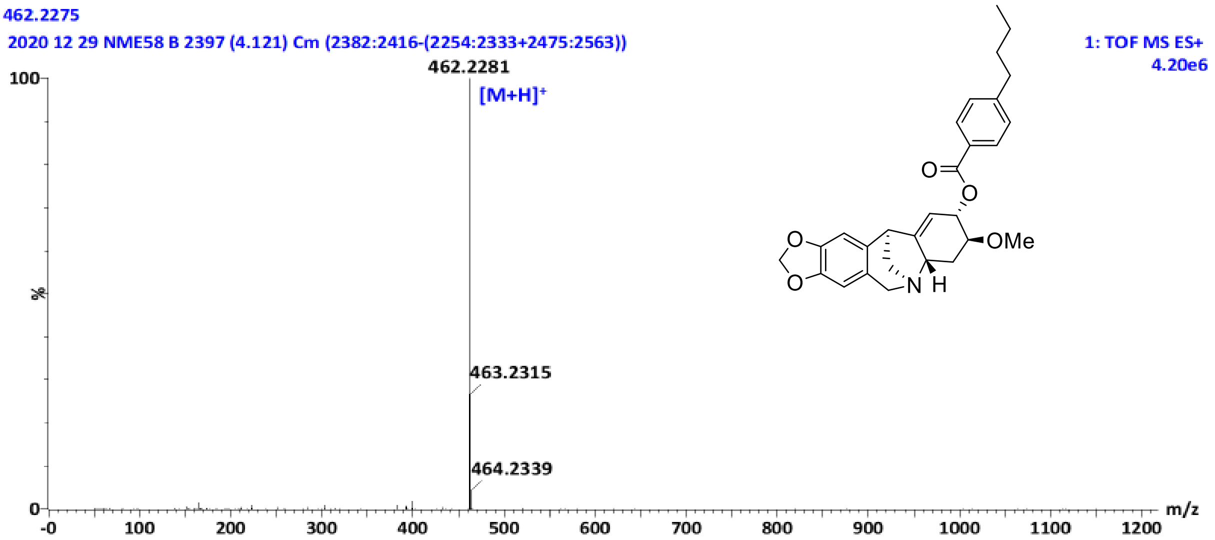

Figure S71. ^{1}H NMR spectrum of 2-*O*-(4-butylbenzoyl)-3-*O*-methylpancracine (**3d**) in $CDCl_3$

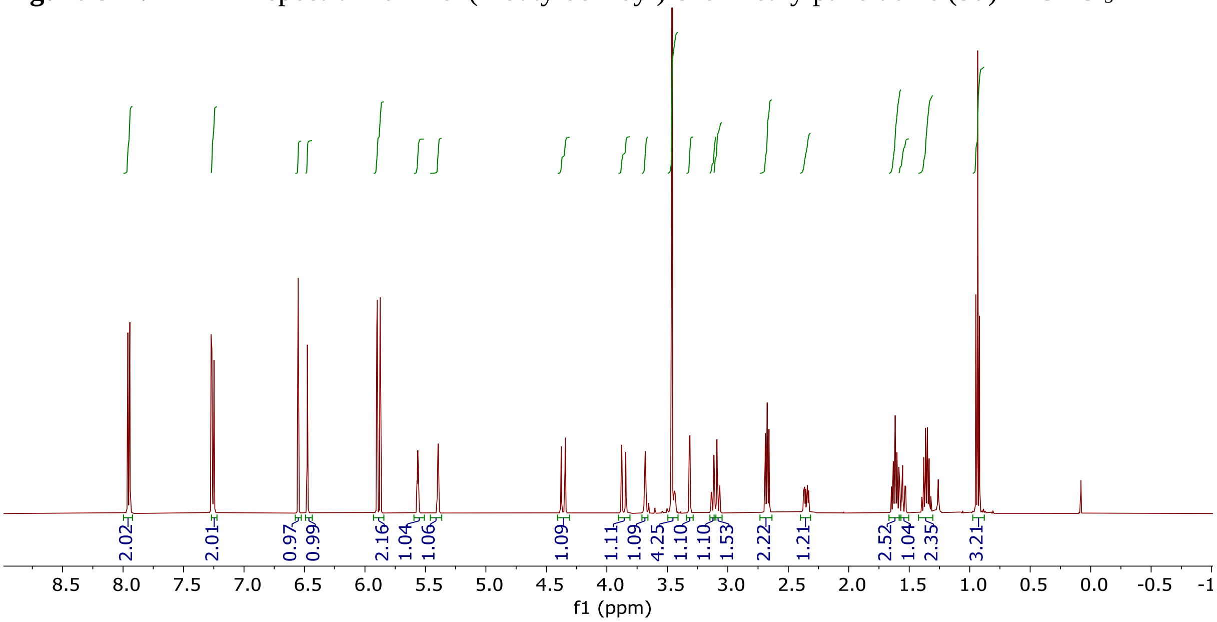

Figure S72. ^{13}C NMR spectrum of 2-*O*-(4-butylbenzoyl)-3-*O*-methylpancracine (**3d**) in $CDCl_3$

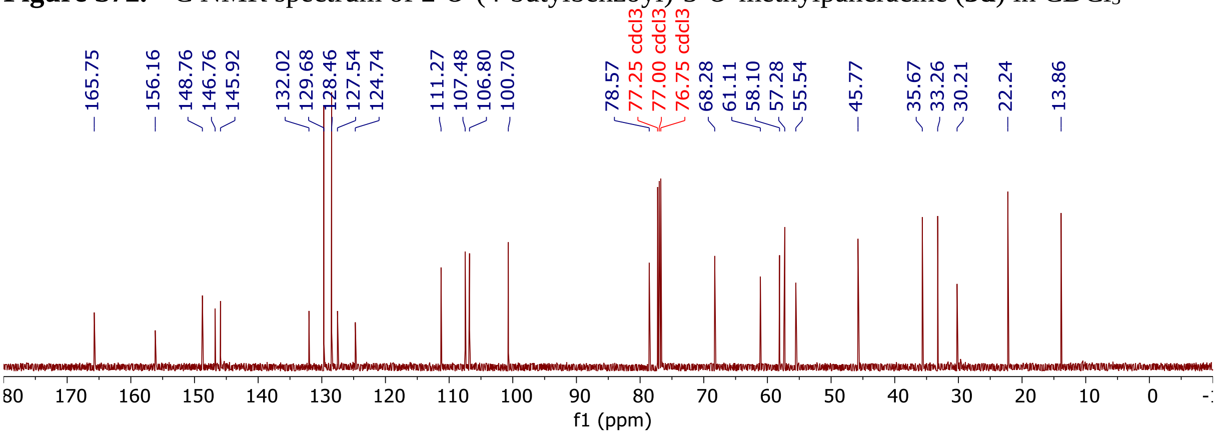

Figure S73. ESI-HRMS spectrum of 2-*O*-(3,5-diethoxybenzoyl)-3-*O*-methylpancracine (**3e**)

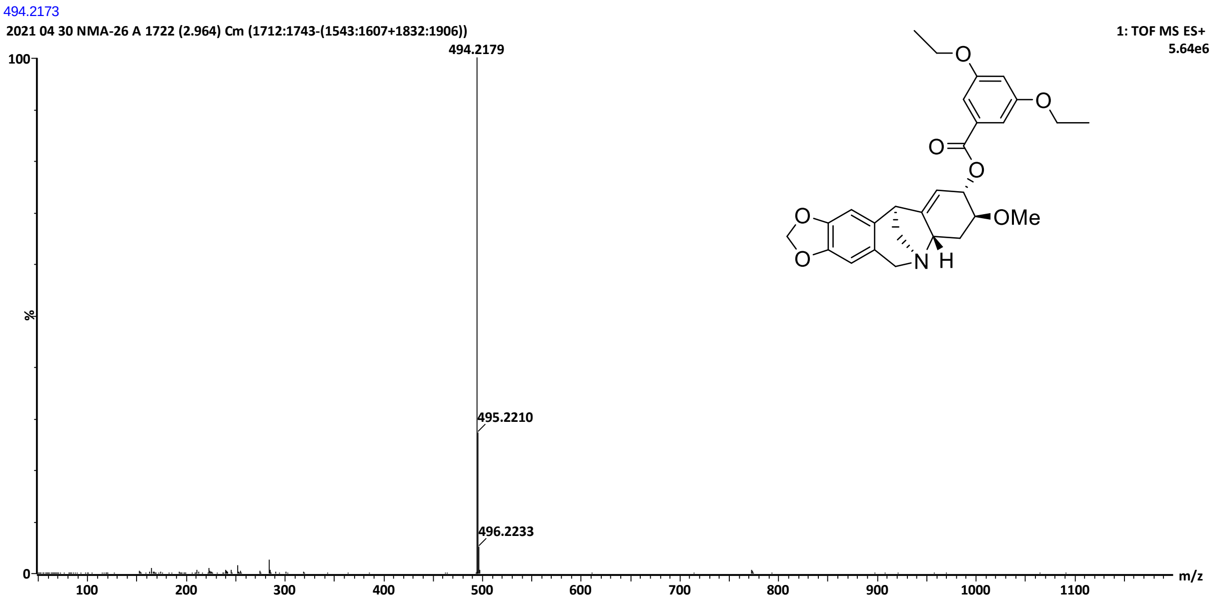

Figure S74. ^{1}H NMR spectrum of 2-*O*-(3,5-diethoxybenzoyl)-3-*O*-methylpancracine (**3e**) in $CDCl_3$

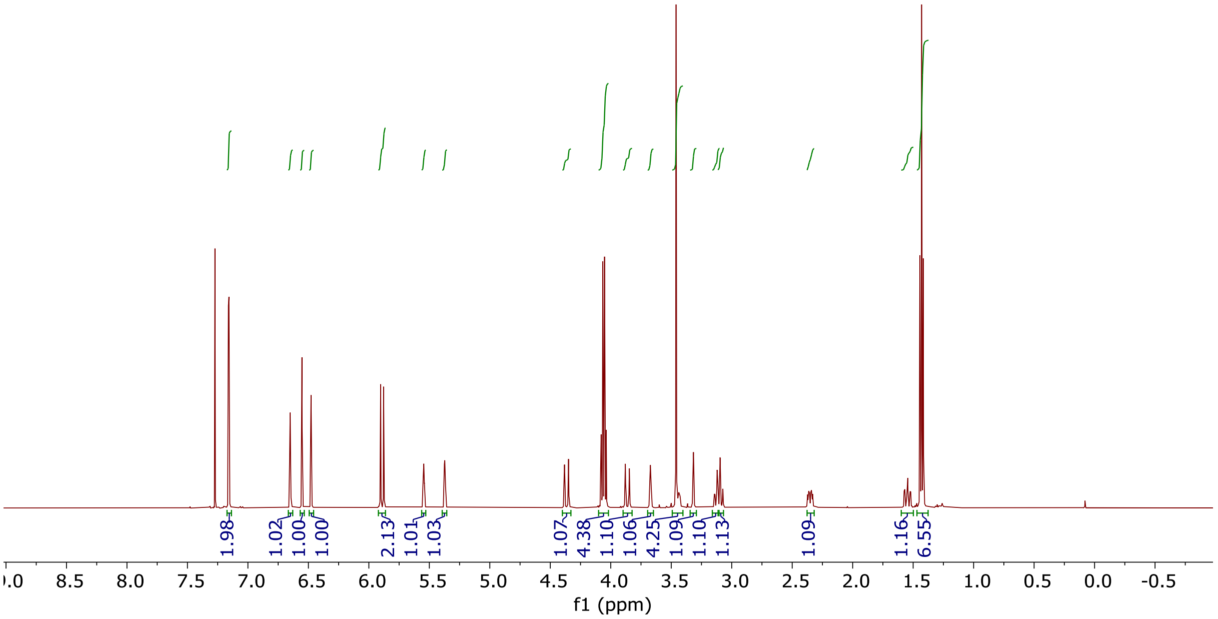

Figure S75. ^{13}C NMR spectrum of 2-*O*-(3,5-diethoxybenzoyl)-3-*O*-methylpancracine (**3e**) in $CDCl_3$

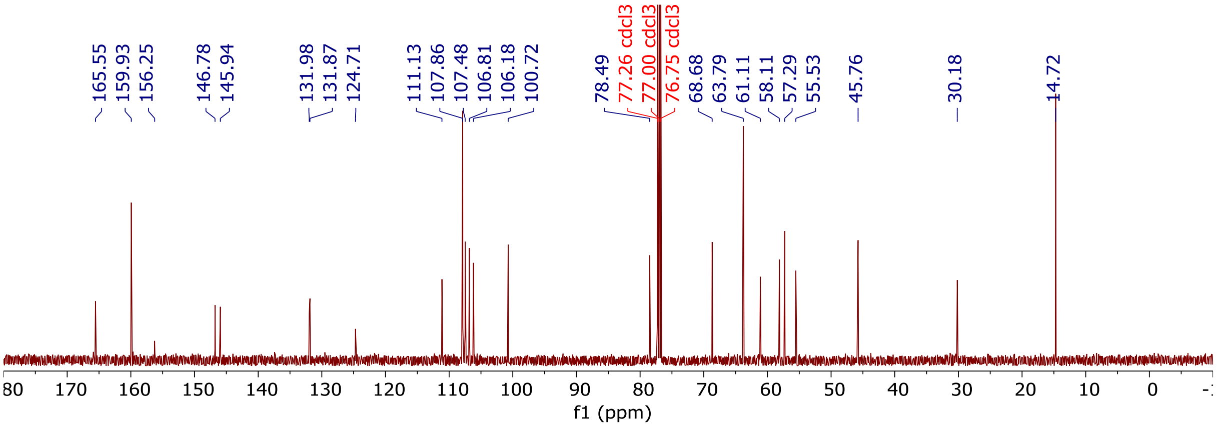

Figure S76. ESI-HRMS spectrum of 2-*O*-(1-naphtoyl)-3-*O*-methylpancracine (**3f**)

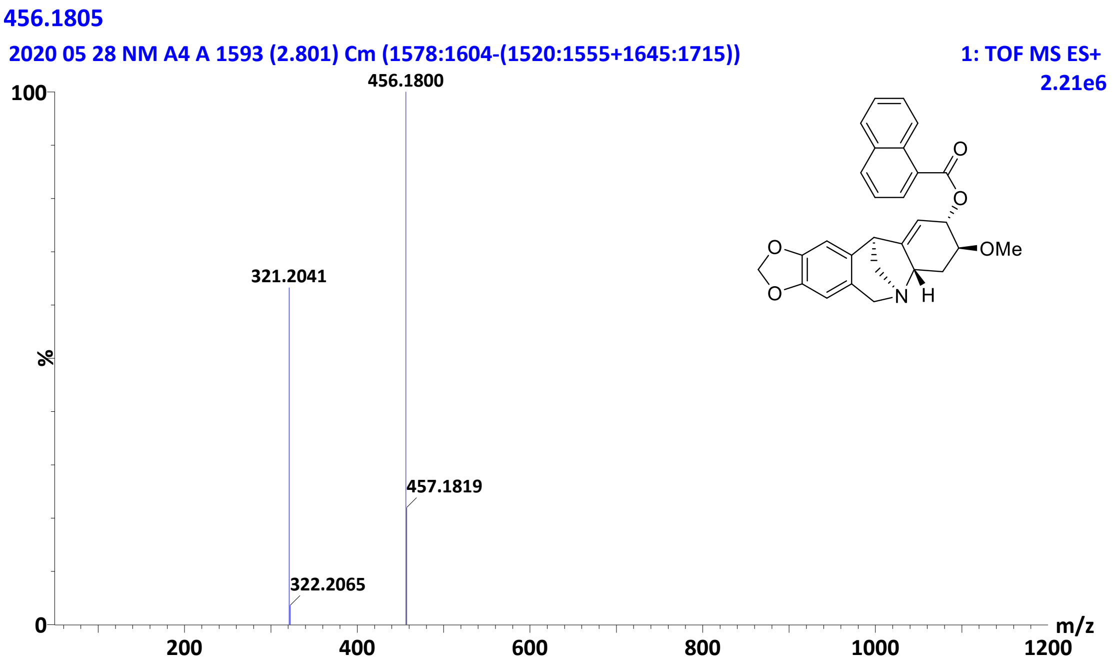

Figure S77. ^{1}H NMR spectrum of 2-*O*-(1-naphtoyl)-3-*O*-methylpancracine (**3f**) in $CDCl_3$

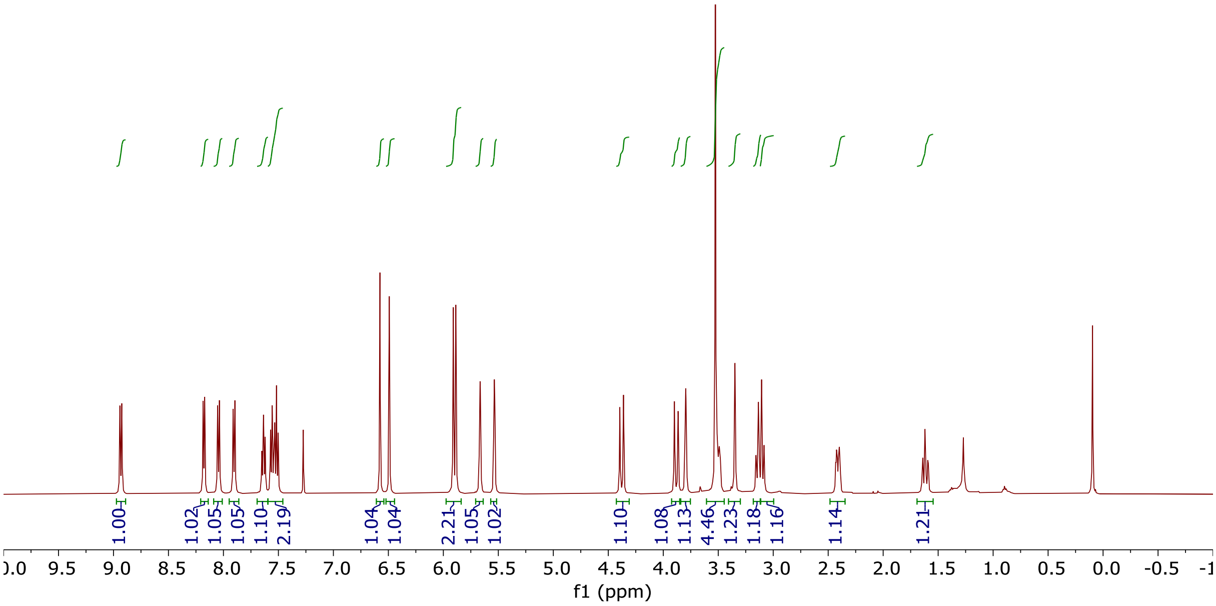

Figure S78. ^{13}C NMR spectrum of 2-*O*-(1-naphtoyl)-3-*O*-methylpancracine (**3f**) in $CDCl_3$

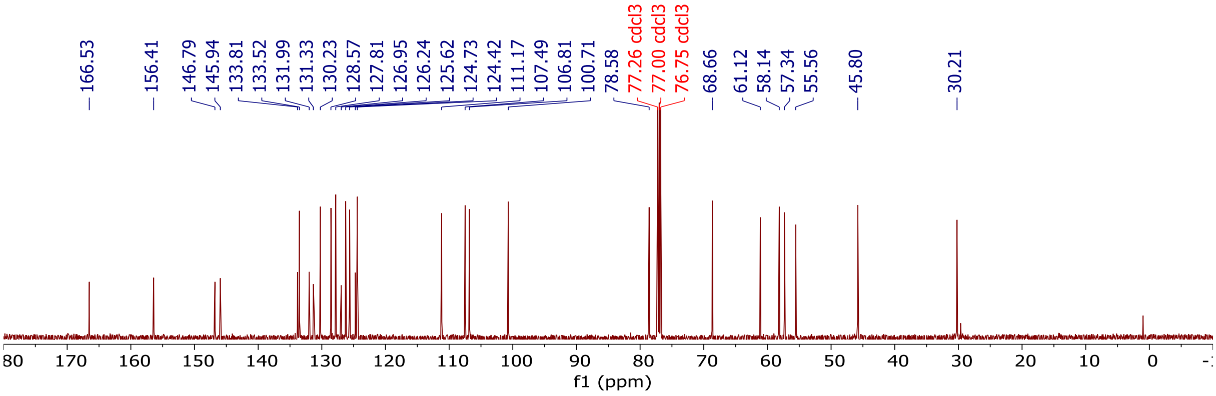

Figure S79. ESI-HRMS spectrum of 2-*O*-(2-naphtoyl)-3-*O*-methylpancracine (**3g**)

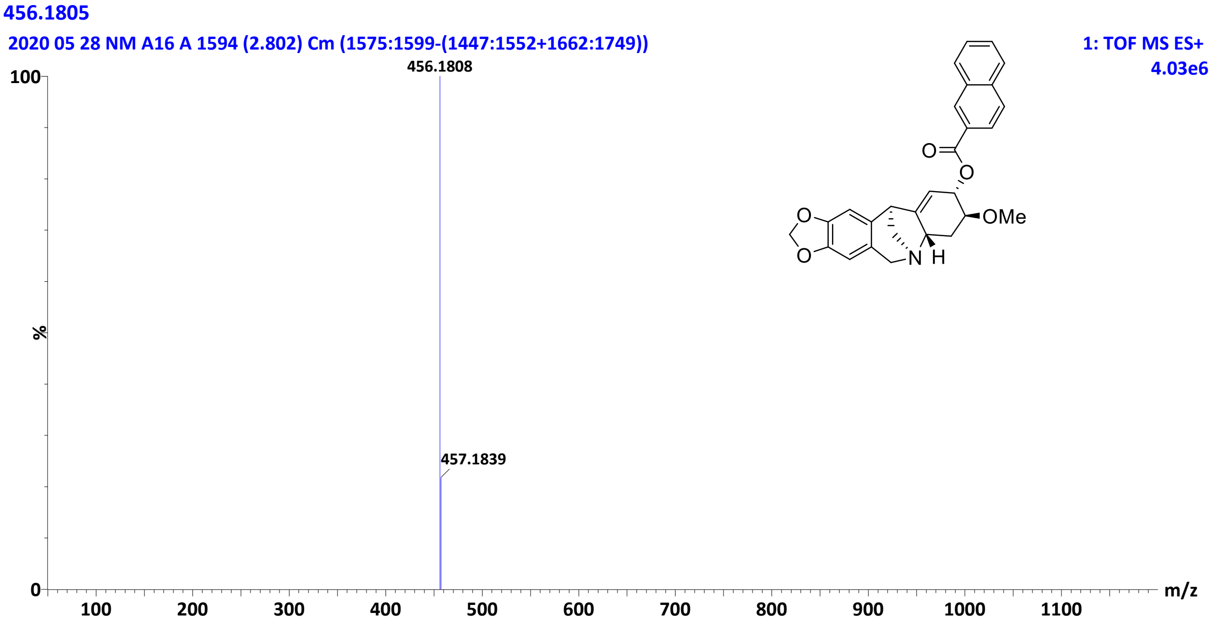

Figure S80. ^{1}H NMR spectrum of 2-*O*-(2-naphtoyl)-3-*O*-methylpancracine (**3g**) in $CDCl_3$

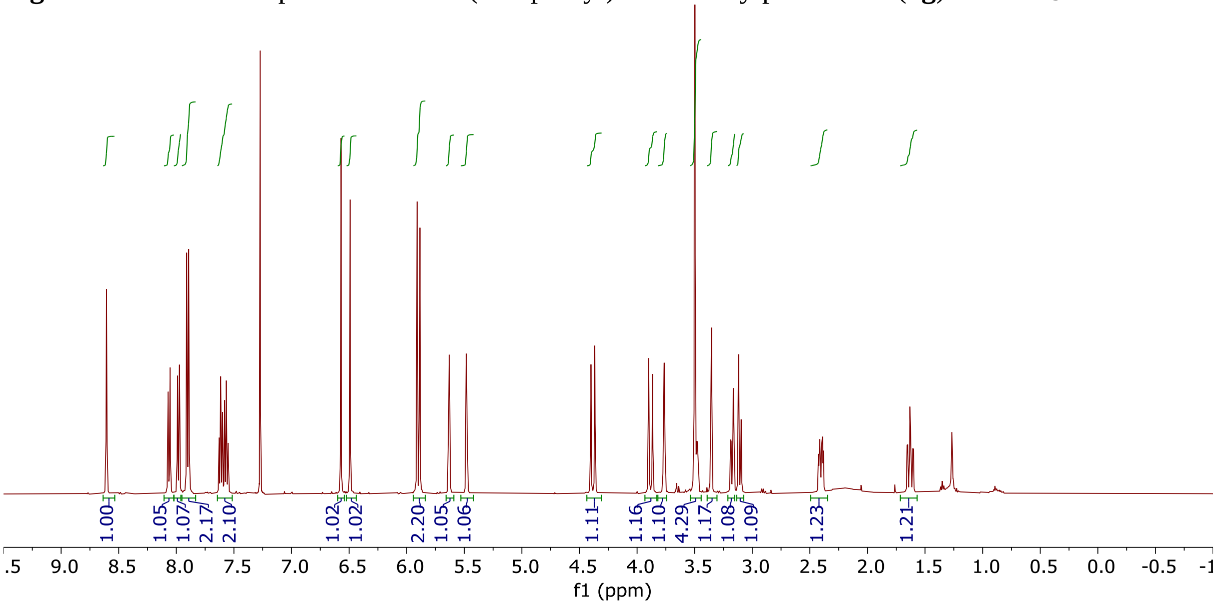

Figure S81. ^{13}C NMR spectrum of 2-*O*-(2-naphtoyl)-3-*O*-methylpancracine (**3g**) in $CDCl_3$

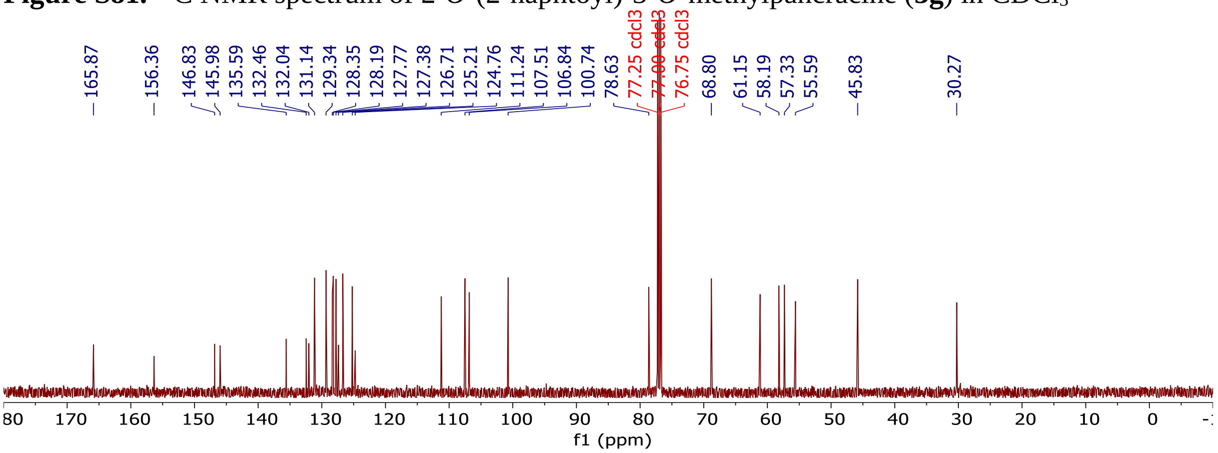

Figure S82. ESI-HRMS spectrum of 3-*O*-(1-naphtoyl)vittatine (**4a**)

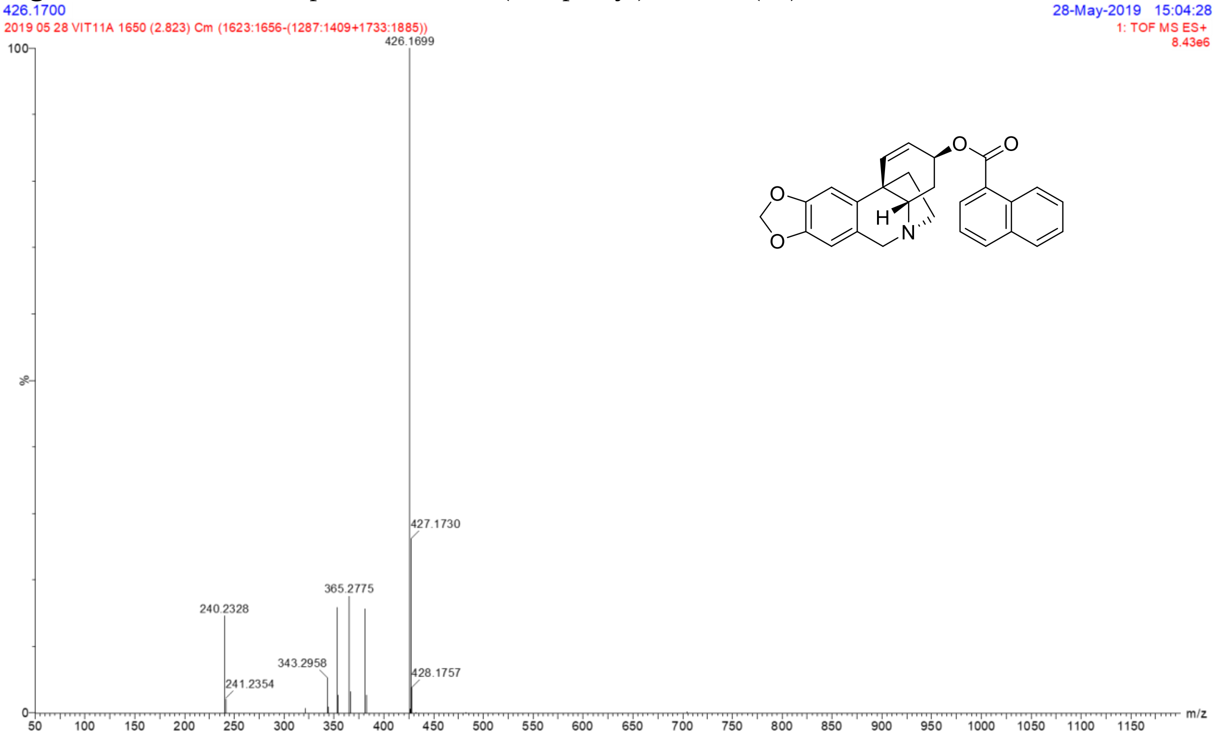

Figure S83. ^{1}H NMR spectrum of 3-*O*-(1-naphtoyl)vittatine (**4a**) in $CDCl_3$

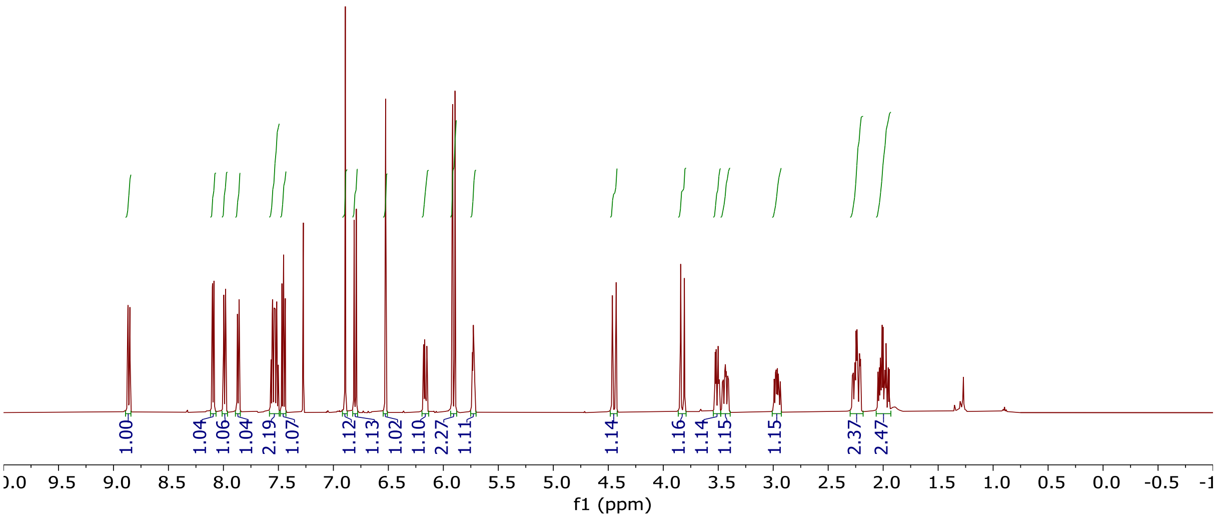

Figure S84. ^{13}C NMR spectrum of 3-*O*-(1-naphtoyl)vittatine (**4a**) in $CDCl_3$

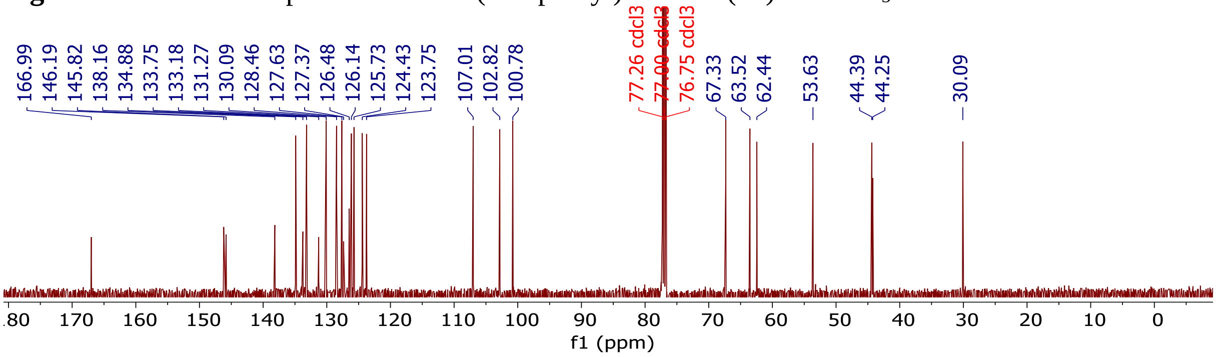

Figure S85. ESI-HRMS spectrum of 3-*O*-(2-naphtoyl)vittatine (**4b**)

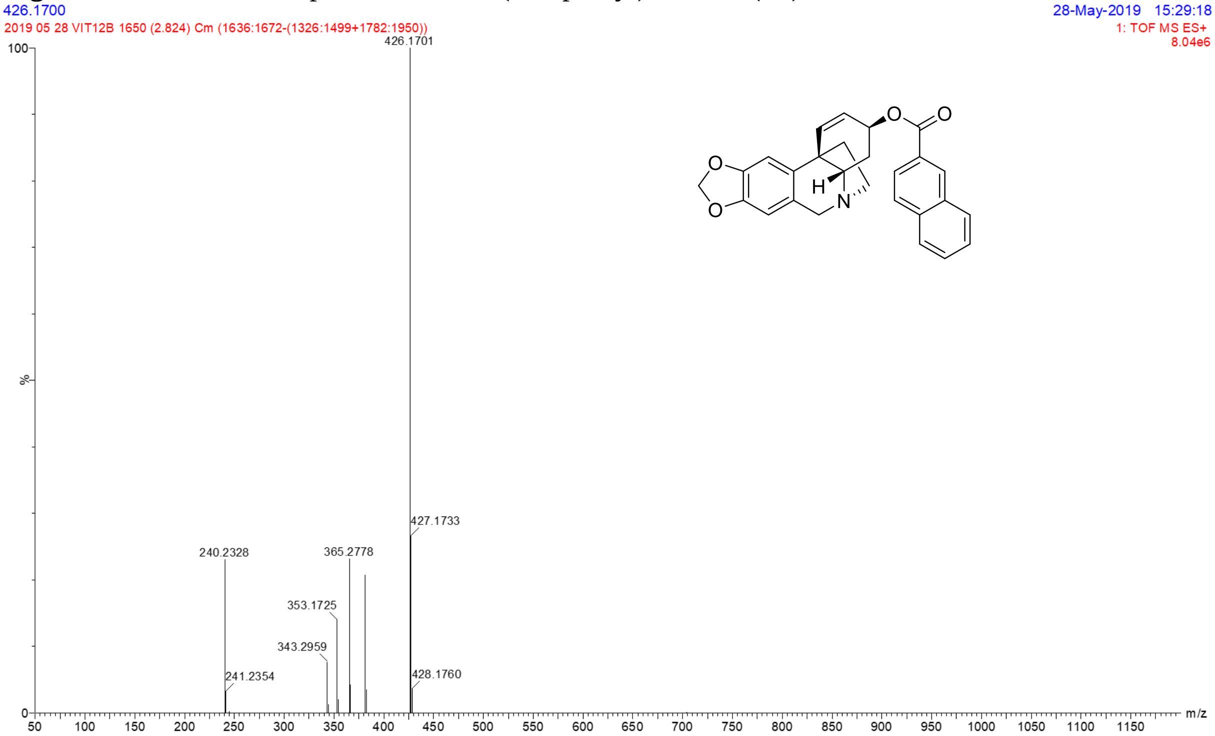

Figure S86. ^{1}H NMR spectrum of 3-*O*-(2-naphtoyl)vittatine (**4b**) in $CDCl_3$

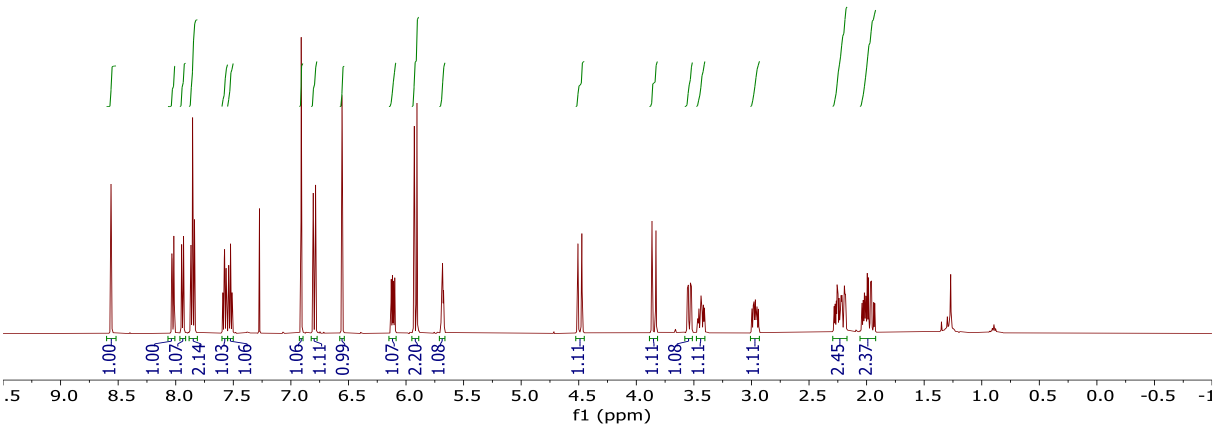

Figure S87. ^{13}C NMR spectrum of 3-*O*-(2-naphtoyl)vittatine (**4b**) in $CDCl_3$

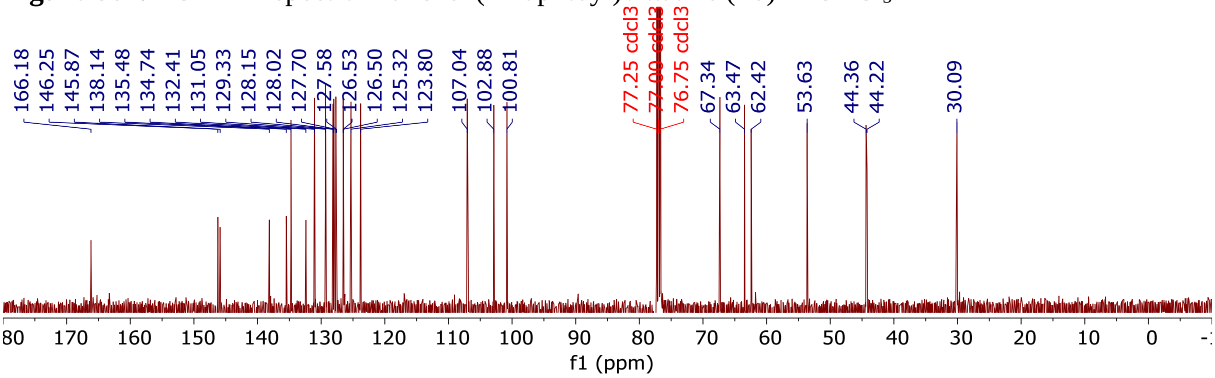

Figure S88. ESI-HRMS spectrum of 3-*O*-(3,5-dimethylbenzoyl)maritidine (**5a**)

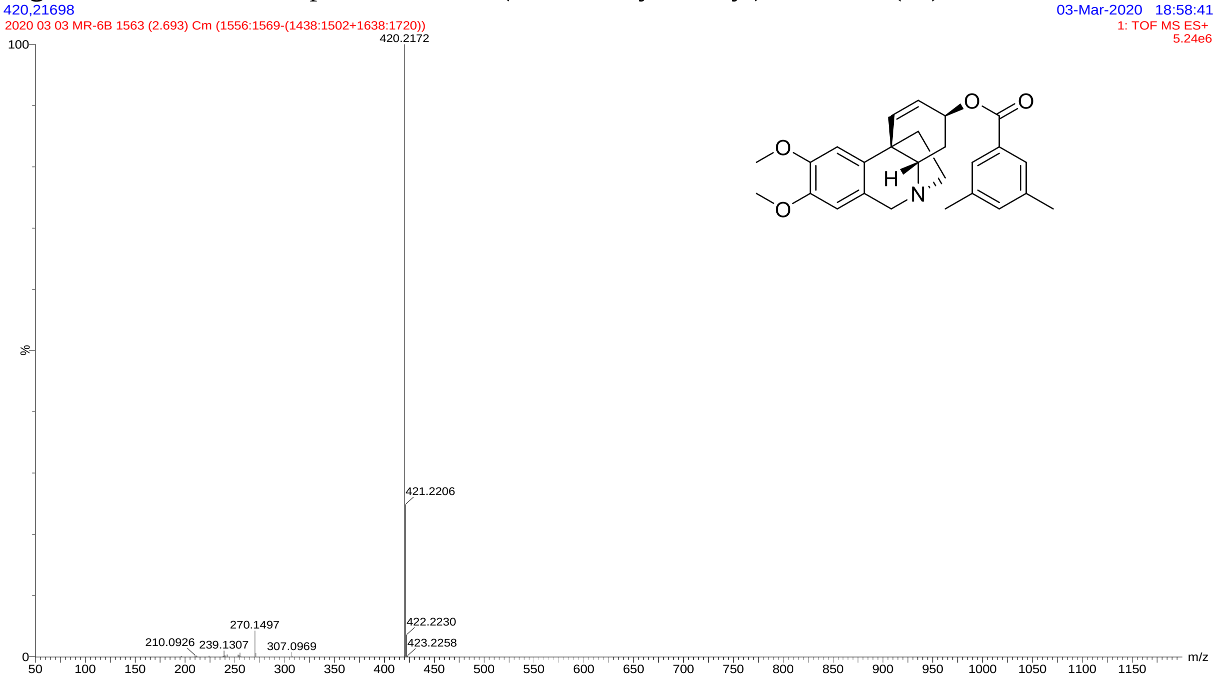

Figure S89. ^{1}H NMR spectrum of 3-*O*-(3,5-dimethylbenzoyl)maritidine (**5a**) in $CDCl_3$

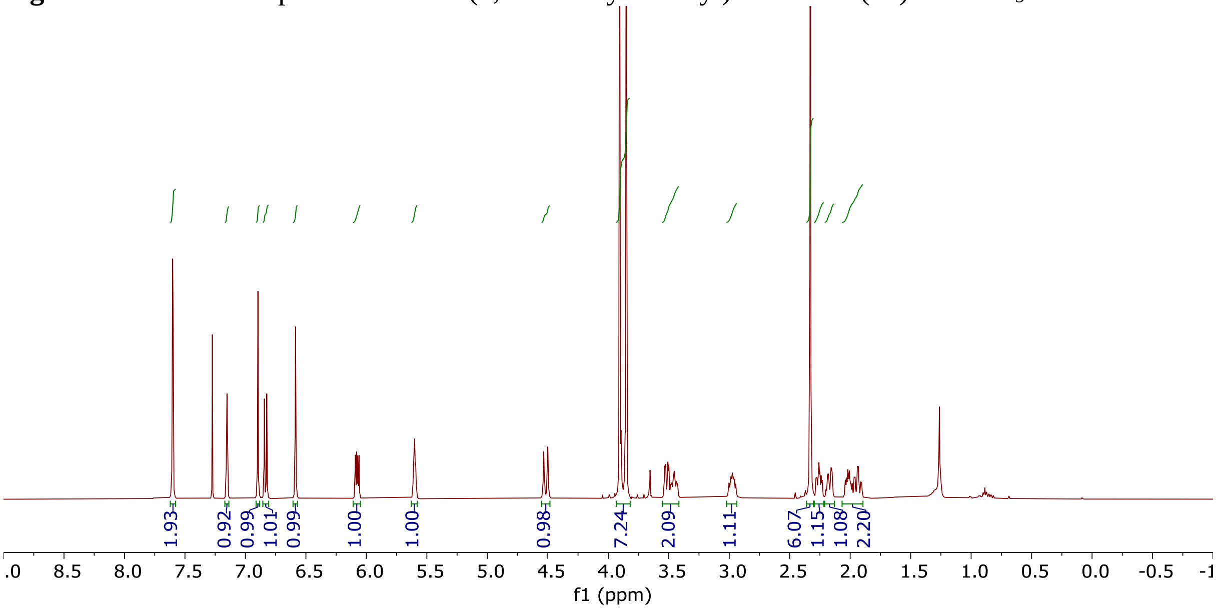

Figure S90. ^{13}C NMR spectrum of 3-*O*-(3,5-dimethylbenzoyl)maritidine (**5a**) in $CDCl_3$

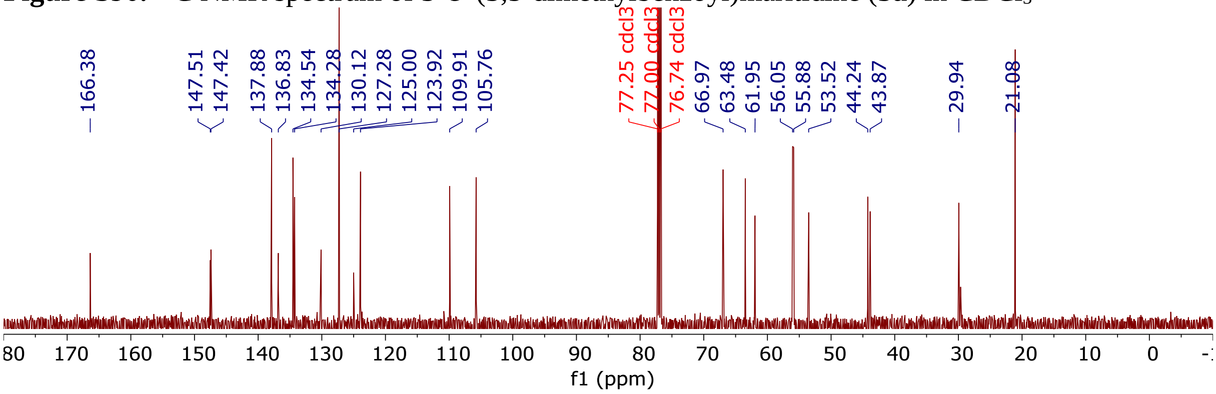

Figure S91. ESI-HRMS spectrum of 3-*O*-(1-naphtoyl)maritidine (**5b**)

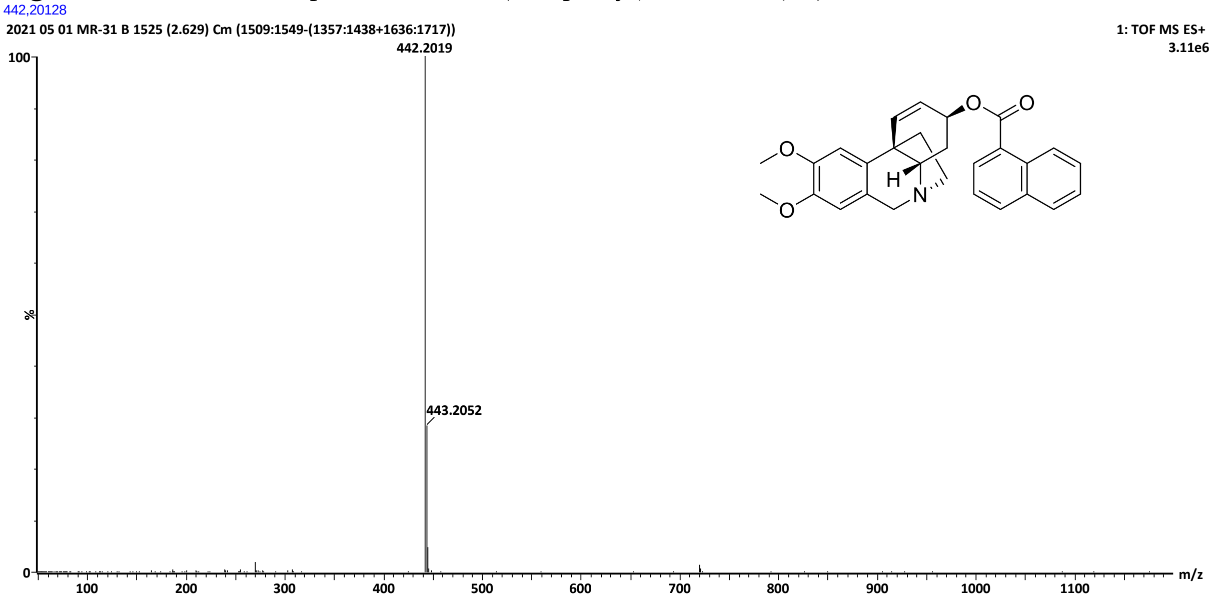

Figure S92. ^{1}H NMR spectrum of 3-*O*-(1-naphtoyl)maritidine (**5b**) in $CDCl_3$

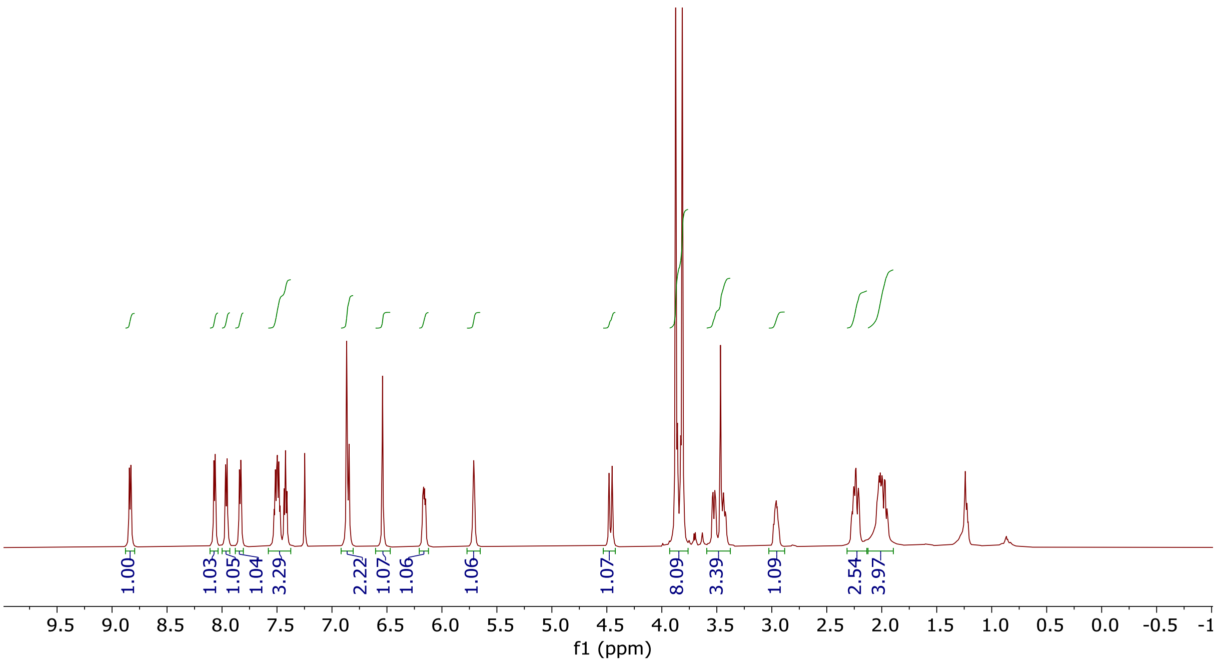

Figure S93. ^{13}C NMR spectrum of 3-*O*-(1-naphtoyl)maritidine (**5b**) in $CDCl_3$

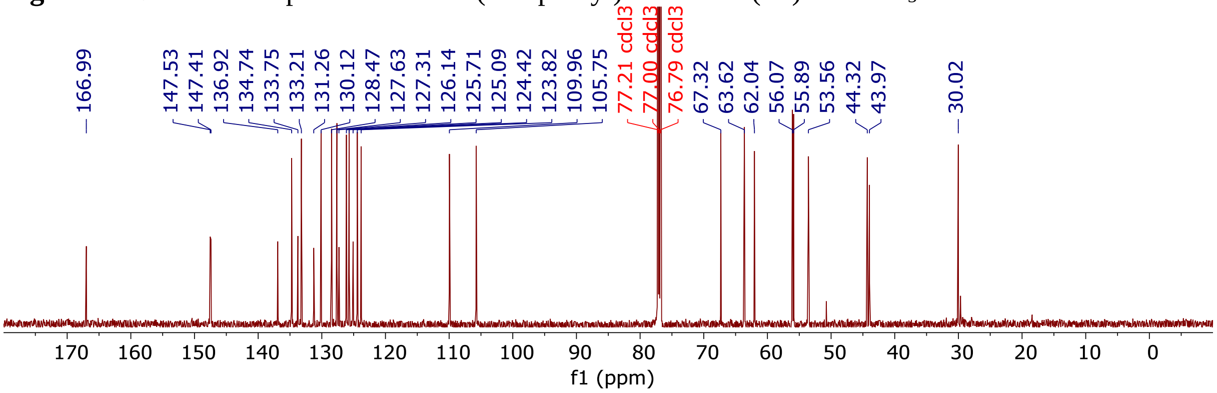

Supplement: Supplementary file 1 [file molecules-26-06023-s001.zip › molecules-1392146-supplementary.pdf]
